# Regulatory Science to 2025: An Analysis of Stakeholder Responses to the European Medicines Agency's Strategy

**DOI:** 10.3389/fmed.2020.00508

**Published:** 2020-09-23

**Authors:** Philip A. Hines, Rosa Gonzalez-Quevedo, Apolline I. O. M. Lambert, Rosanne Janssens, Barbara Freischem, Jordi Torren Edo, Ivo J. T. M. Claassen, Anthony J. Humphreys

**Affiliations:** ^1^European Medicines Agency, Amsterdam, Netherlands; ^2^United Nations University—Maastricht Economic and Social Research Institute on Innovation & Technology (UNU-MERIT), Maastricht University, Maastricht, Netherlands; ^3^Clinical Pharmacology and Pharmacotherapy, KU Leuven, Leuven, Belgium

**Keywords:** regulatory science, regulation, innovation, stakeholder engagement, patient access, benefit-risk assessment

## Abstract

The pace of innovation is accelerating, and so medicines regulators need to actively innovate regulatory science to protect human and animal health. This requires consideration and consultation across all stakeholder groups. To this end, the European Medicines Agency worked with stakeholders to draft its *Regulatory Science Strategy to 2025* and launched it for public consultation. The responses to this consultation were analyzed qualitatively, using framework analysis and quantitatively, to derive stakeholders' aggregate scores for the proposed recommendations. This paper provides a comprehensive resource of stakeholder positions on key regulatory science topics of the coming 5 years. These stakeholder positions have implications for the development and regulatory approval of both human and veterinary medicines.

## Introduction

The pace of innovation has accelerated, at the same time as medicines become more complex. This innovation is occurring across the entire medicine lifecycle, from candidate screening and characterization to pharmacovigilance and repurposing medicine. To remain capable of guiding and regulating these emerging science and technologies—and thus protecting human and animal health—regulators must remain abreast of all of them (1). Business as usual is not sufficient to do this; regulators need to actively innovate regulatory science and processes themselves (2–4) in line with medicine users' needs.

In view of the breadth of emerging regulatory science topics, future-proofing regulatory science requires strategic direction. A comprehensive strategy including scientific, regulatory, operational, and resourcing is required to regulate the growing ecosystem of innovation in the development of human and veterinary medicines. This strategic response must also leverage and advance collaborative approaches to evolve evidence generation and medicines development, such as new methods to replace, reduce and refine animal models; systematic patient engagement; use of digital and real-world data in clinical settings for pre and post authorisation benefit-risk. In the European context, this collaborative approach can be coupled with a greater integration with downstream decision makers, such as health technology assessment (HTA) bodies and payers, to expedite patient-centered access to innovative human medicine.

Beyond science and technological innovation—and as shown by the COVID-19 pandemic—regulators must also continually advance preparedness for emerging health threats such as in antimicrobial resistance (AMR) and medicine supply and seize opportunities to mitigate them, such as repurposing medicines.

As a public institution that is part of a network with national competent authorities and involves in its work stakeholders, any such strategy must be made in consultation of this network and its stakeholders. To this end, EMA undertook a highly collaborative approach, building upon previous methods used by the agency, including interviews, workshops, and stakeholder consultations. However, here the agency has sought to maximize its effectiveness through applying social science methods including qualitative semi-structured interviews and quantitative preference elucidation through Likert scales (5–8). These consultative methods were firstly used to draft a strategy which was put out for public consultation. The results of this consultation fed into the final strategy: EMA Regulatory Science to 2025 (RSS) (9).

The aim of this paper is to disseminate the stakeholders' views collected during the public consultation on the broad range of regulatory science topics in the draft regulatory science strategy, from the well-established to the emerging areas. This paper provides a comprehensive resource for readers to understand stakeholders' positions on topics for the coming 5 years and increases transparency on the views incorporated into EMA's strategy.

## Materials and Methods

### Strategy and Survey Development

First, a baseline literature review and horizon scanning were conducted across 60 areas of science, technology, and health to map the anticipated challenges and opportunities for the next 10 years. The findings were then validated and supplemented by 70 interviews with a range of European Medicines Regulatory Network (EMRN) stakeholders. The information gathered was used to draw up the first draft of the agency's regulatory science strategy (10). Stakeholders were invited to workshops to provide initial feedback on the draft strategy. Subsequently, stakeholders were also invited to participate in a public consultation of the draft strategy, launched online for a period of 6 months, to express their specific views on an updated version of draft strategy.

The online survey tool, EUSurvey (11), was used to gather stakeholder views on the regulatory science topics in the RSS. The survey was designed by means of (a) discussions within EMA to identify what information we want from stakeholders and the phrasing of the respective questions, (b) alignment of these questions with the overarching goals of the RSS, and (c) trialing the survey with colleagues for further refinement.

The survey comprised 14 questions: 7 questions on the human side and 7 equivalent questions on the veterinary side of the RSS (Supplementary Material). Qualitative information was gathered through free text boxes and quantitative preference elucidation by ranking and Likert scales. A 5-point Likert scale (not important; less important; moderately important; important; very important) was used for Question 7, to which responders could provide more detailed feedback on their prioritization.

The survey was shared with the public through a press release and announcements on EMA's website, and key stakeholder groups were targeted via email and a multi-stakeholder launch workshop. The survey opened on December 19, 2018, and ran to June 30, 2019. During this time, the frequency of responses from stakeholder groups was monitored, and those with low response numbers were further targeted via email and at other stakeholder meetings.

After the public consultation, there were follow-up workshops in 2019 to review the preliminary analysis and their implementation.

### Analysis of Survey Responses

**Figure 1:**
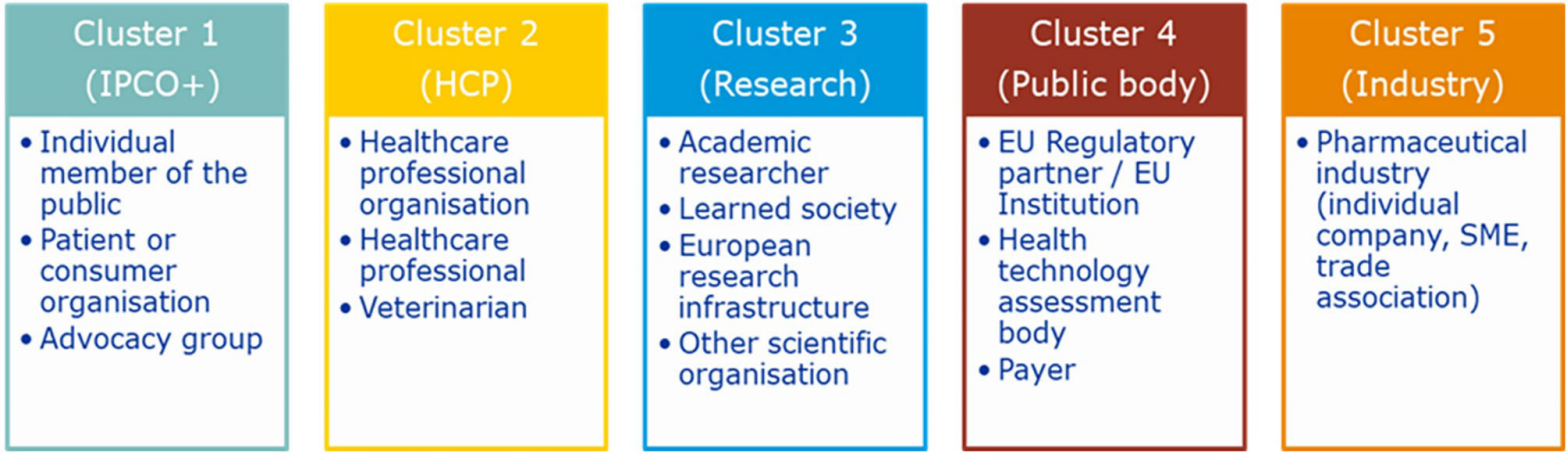
Clustering of survey respondents. The survey respondents were clustered into 5 groups: cluster 1 (IPCO+) contained “individual members of the public,” “patient or consumer organizations,” and “advocacy groups”; cluster 2 (HCP) contained “health care professional organizations,” “health care professionals,” and “veterinarians”; cluster 3 (Research) contained “academic researchers” “learned society,” “European research infrastructures,” and “other scientific organizations”; cluster 4 (public bodies), contained “EU regulatory partners/EU institutions,” “health technology assessment bodies,” and “payers”; cluster 5 (Industry) contained “pharmaceutical industry” (“individual companies,” “SMEs,” and “trade associations”).

**Figure 2:**
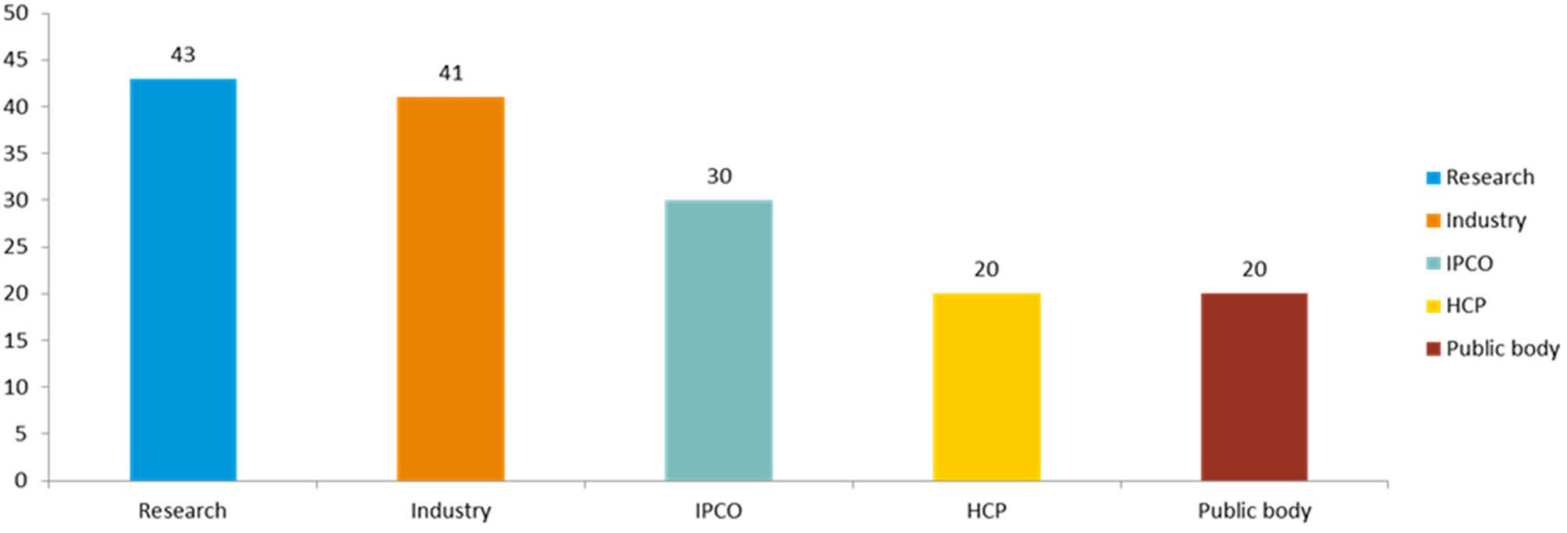
Number of responses received to the public consultation by stakeholder type.

The stakeholders were clustered into groups (Figures 1, 2). All results were subject to a partially blind analysis: the information about the respondents was separated from the responses, and the order of responses was mixed. Due to the partially blind nature of the analysis, the responses were weighted neutrally, meaning that each response was counted as one participant, regardless of the size of the stakeholder, or where there were combined responses of several individuals, e.g., association vs. individual. To adjust for any potential sample size bias this may create, the unblinded responses, which contain the stakeholder size and whether the response was a combined one, were fed into the finalization of the RSS.

#### Quantitative Analysis

Responses to the survey's questions 5 and 7 were analyzed descriptively in Microsoft Excel (the survey questions can be found in the Supplementary Material). For question 5, stakeholders had to identify, in order of importance, their top three Core Recommendations that they believed would deliver the most significant change in the regulatory system over the next 5 years. The analysis looked at the total number of times a Core Recommendation was selected as first, second, and third choice across the stakeholder clusters.

The 5-point Likert scale was used for question 7. Quantifiable values from 1 to 5 were assigned to the Likert scale to weigh the responses: not important = 1; less important = 2; moderately important = 3; important = 4; very important = 5. The overall mean score for each Core Recommendation was calculated, and the mean scores for all Core Recommendations were compared. In addition, a sub-analysis calculated the mean score by Core Recommendation for each stakeholder cluster. Stakeholders were asked not to provide feedback to areas outside of their interest or experience.

#### Qualitative Data Analysis

Responses to the survey's open-ended questions 3, 5, 6, and 7 were analyzed thematically by four researchers. Questions 1 and 2 gathered information about the respondents, and responses to question 4 were further developed in questions 5, 6, and 7. Questions 3, 5, 6, and 7 asked about stakeholders' overall views of the RSS (question 3); whether and which elements they considered missing in the strategy (question 6) and feedback on the Core Recommendations and underlying actions (questions 5 and 7). The framework method was chosen for thematic analysis as it enables multiple researchers to independently analyze one large dataset (7, 8). Table 1 explains the implementation of the five iterative stages of the framework method as explained by Lacey and Luff, 2007: (1) familiarization, (2) identifying a thematic framework, (3) coding, (4) summarizing, and (5) mapping and interpretation.

**Table 1:** Iterative stages of the framework method.

**Stages**	**Description**
Familiarization	The answers to the open-ended questions 3, 5, 6, and 7 of the survey were thoroughly read and reread by the researchers involved in the analysis. The researchers discussed among each other to clarify and better understand those answers that were less clear or confusing
Identifying a thematic framework	The researchers independently assigned a label to participants' answers (“coding,” see step 3) before meeting to develop the initial list of codes, i.e., the initial thematic framework. During these meetings, the researchers discussed why they coded a certain piece of text, i.e., why they perceived it to be meaningful. The thematic framework was further developed and refined during the subsequent stages
Coding	The coding was both guided by the structure of the regulatory science strategy (the Core Recommendations) and what was in the responses (“open coding”). The researchers coded the text using paper and pen, Microsoft Word, or Excel
Summarizing	Responses were summarized by question, by stakeholder group, and/or by recommendation in Microsoft Word. In the veterinary summaries and in some human ones, a limited number of responses necessitated their pooling across stakeholders to create a summary. Only themes identified by two or more responses could enter a summary, as otherwise it would not be a summary. In a stepwise manner, the researchers (i) drafted a summary for each question, stakeholder group, and/or Core Recommendation (ii) convened to discuss and reach consensus about these summaries
Mapping and interpretation	Using the summaries created in stage 4, the researchers searched for themes in the data. This process was guided by the survey questions (“deductively”) and a careful analysis of what was in the data (“inductively”). Interpretations were made by discussing and reviewing the summaries and by making associations within and across stakeholder groups. Whenever the data were rich enough, the interpretations generated in this stage went beyond the description of particular responses to the explanation of potential reasons or beliefs expressed by participants.

##### Characterization of survey respondents

Following the partially blinded qualitative analysis, authors performed a basic characterization of survey respondents within each stakeholder cluster to check for representativeness of the results (see “Overall number of responses to human and veterinary” and Figure 2).

## Results

### Overall Number of Responses to the Human and Veterinary Questions

A total of 154 responses to the survey were received. Of these, 130 replied only to the human part, 7 only to the veterinary part and 17 to both sections.

There were 7 responses from individual members of the public (6 responding to the human side and 1 to the veterinary part) and 23 from patients and consumer organization (responding only to the human section). The latter included the major European consumers' and patients' organizations, covering different therapeutic areas, including neurological conditions, HIV/AIDS, cancer, and immunological and rare diseases. In addition, feedback was received from other respondents belonging to the non-governmental sector or well-established advocacy groups.

A total of 20 responses were from health care professionals (6 of which responded to the veterinary questionnaire): 6 individual health care professionals, 2 veterinarians, and 12 responses from organizations representing national medical and learned societies across Europe, including the major pan-European organizations.

From the research sector there were 43 responses (6 of which responded to the veterinary part), including 4 responses from European research infrastructures; 1 learned society; 1 farming and animal owner organizations; 13 individual researchers; and 24 scientific organizations.

From public bodies, a total of 20 responses were received (6 of which responded to the veterinary part), including 6 from medicines regulatory agencies, 4 from other EU public bodies and 1 from the European Commission. There were 9 responses from downstream decision makers, including 6 HTA bodies and 3 payer organizations.

From industry, 41 responses were received (5 of which responded to the veterinary part), including 11 from the main trade associations spanning all industry types (originator pharmaceuticals and biologicals, generics and biosimilars, vaccines, non-prescription medicines and veterinary medicines). The remainder were from individual companies, including many of the major pharmaceutical companies. Five out of the 41 respondents identified themselves as small and medium enterprises (SMEs).

### Responses to the Human Section of the RSS

#### Quantitative Analysis

**Figure 3:**
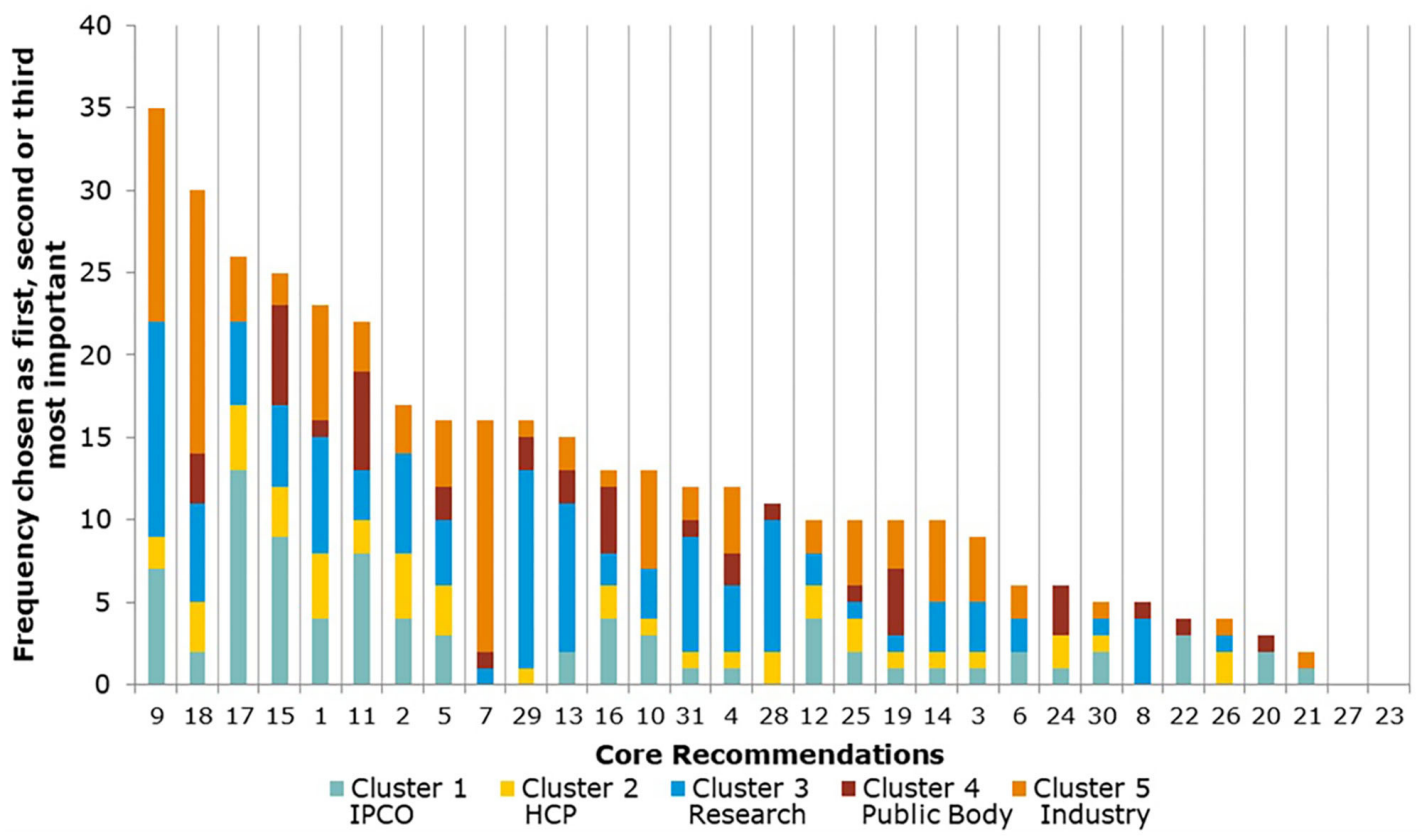
Aggregate ranking of Core Recommendations (question 5, human).

The Core Recommendations were ranked by the frequency with which stakeholders selected them as among their top three choices to deliver “the most significant change in the regulatory system over the next 5 years” (question 5), as shown in Figure 3 and Table 2. Figure 3 also illustrates the overall total scores per cluster group for each Core Recommendation. For example, Core Recommendation 9 (foster innovation in clinical trials) was only the top choice for cluster 3 (the research cluster).

**Table 2:** Aggregate ranking of Core Recommendations (question 5, Human).

**Number**	**Core Recommendations**	**Total^a^**
9	Foster innovation in clinical trials	35
18	Promote use of high-quality real-world data (RWD) in decision making	30
17	Reinforce patient relevance in evidence generation	26
15	Contribute to HTAs' preparedness and downstream decision making for innovative medicines	25
1	Support developments in precision medicine, biomarkers, and “omics”	23
11	Expand benefit-risk assessment and communication	22
2	Support translation of Advanced Therapy Medicinal Products cell, genes, and tissue-based products into patient treatments	17
5	Create an integrated evaluation pathway for the assessment of medical devices, *in vitro* diagnostics, and borderline products	16
7	Diversify and integrate the provision of regulatory advice along the development continuum	16
29	Leverage collaborations between academia and network scientists to address rapidly emerging regulatory science research questions	16
13	Optimize capabilities in modeling and simulation and extrapolation	15
16	Bridge from evaluation to access through collaboration with Payers	13
10	Develop the regulatory framework for emerging digital clinical data generation	13
31	Disseminate and share knowledge, expertise, and innovation across the regulatory network and to its stakeholders	12
4	Facilitate the implementation of novel manufacturing technologies	12
28	Develop network-led partnerships with academia to undertake fundamental research in strategic areas of regulatory science	11
12	Invest in special population initiatives	10
25	Promote global cooperation to anticipate and address supply challenges	10
19	Develop network competence and specialist collaborations to engage with big data	10
14	Exploit digital technology and artificial intelligence in decision making	10
3	Promote and invest in the Priority Medicines scheme (PRIME)	9
6	Develop understanding of and regulatory response to nanotechnology and new materials' utilization in pharmaceuticals	6
24	Continue to support development of new antimicrobials and their alternatives	6
30	Identify and enable access to the best expertise across Europe and internationally	5
8	Leverage novel non-clinical models and 3Rs	5
22	Further develop external communications to promote trust and confidence in the EU regulatory system	4
26	Support innovative approaches to the development and post-authorization monitoring of vaccines	4
20	Deliver real-time electronic Product Information (ePI)	3
21	Promote the availability and uptake of biosimilars in health care systems	2
27	Support the development and implementation of a repurposing framework	0
23	Implement EMA's health threats plan, ring-fence resources and refine preparedness approaches	0

a *Total times chosen as first, second, or third most important*.

**Figure 4:**
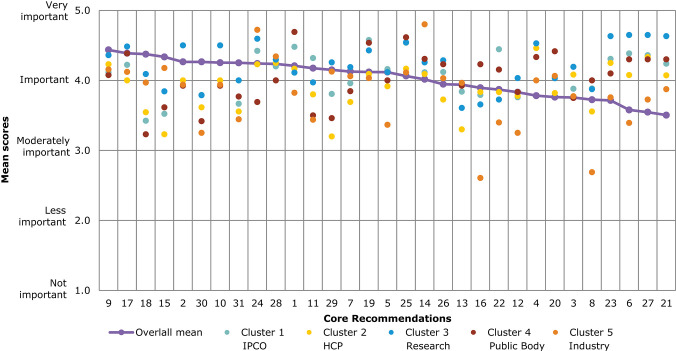
Overall comparison of all Core Recommendations by mean score across stakeholder clusters (question 7). Overall means were calculated based on the individual scores given by participants who rated each Core Recommendation on a scale from 1 to 5: (1) not important; (2) less important; (3) moderately important; (4) important; and (5) very important.

A comparison of overall mean scores per Core Recommendation shows those that were considered the most important (Figure 4 and Table 3). As seen from Table 3, no recommendation scored an overall mean below 3 (Moderately important). A subtle ranking can be observed in the overall mean, with Core Recommendations 9, 17, and 18 all with the highest overall mean score of 4.4. Each Core Recommendation had an average of 115 ratings.

**Table 3:** Overall comparison of all Core Recommendations by mean score (question 7).

**Number**	**Core Recommendations**	**Mean score**
9	Foster innovation in clinical trials	4.4
17	Reinforce patient relevance in evidence generation	4.4
18	Promote use of high-quality real world data (RWD) in decision making	4.4
15	Contribute to HTAs' preparedness and downstream decision making for innovative medicines	4.3
2	Support translation of Advanced Therapy Medicinal Products cell, genes, and tissue-based products into patient treatments	4.3
30	Identify and enable access to the best expertise across Europe and internationally	4.3
10	Develop the regulatory framework for emerging digital clinical data generation	4.3
31	Disseminate and share knowledge, expertise, and innovation across the regulatory network and to its stakeholders	4.3
24	Continue to support development of new antimicrobials and their alternatives	4.2
28	Develop network-led partnerships with academia to undertake fundamental research in strategic areas of regulatory science	4.2
1	Support developments in precision medicine, biomarkers, and “omics”	4.2
11	Expand benefit-risk assessment and communication	4.2
29	Leverage collaborations between academia and network scientists to address rapidly emerging regulatory science research questions	4.2
7	Diversify and integrate the provision of regulatory advice along the development continuum	4.1
19	Develop network competence and specialist collaborations to engage with big data	4.1
5	Create an integrated evaluation pathway for the assessment of medical devices, *in vitro* diagnostics, and borderline products	4.1
25	Promote global cooperation to anticipate and address supply challenges	4.1
14	Exploit digital technology and artificial intelligence in decision making	4.0
26	Support innovative approaches to the development and post-authorization monitoring of vaccines	3.9
13	Optimize capabilities in modeling and simulation and extrapolation	3.9
16	Bridge from evaluation to access through collaboration with payers	3.9
22	Further develop external communications to promote trust and confidence in the EU regulatory system	3.9
12	Invest in special populations initiatives	3.8
4	Facilitate the implementation of novel manufacturing technologies	3.8
20	Deliver real-time electronic Product Information (ePI)	3.8
3	Promote and invest in the Priority Medicines scheme (PRIME)	3.8
8	Leverage novel nonclinical models and 3Rs	3.7
23	Implement EMA's health threats plan, ring-fence resources, and refine preparedness approaches	3.7
6	Develop understanding of and regulatory response to nanotechnology and new materials' utilization in pharmaceuticals	3.6
27	Support the development and implementation of a repurposing framework	3.5
21	Promote the availability and uptake of biosimilars in healthcare systems	3.5

*Overall mean scores were calculated based on the individual scores given by participants who rated each Core Recommendation on a scale from 1 to 5: (1) not important; (2) less important; (3) moderately important; (4) important; (5) very important*.

Figure 4 illustrates the different views of each stakeholder cluster per Core Recommendation. The biggest differences across stakeholder clusters were observed for the Core Recommendations with the lowest overall mean scores, namely, recommendations 21 and 27. For 21 (“Promote availability and uptake of biosimilars in healthcare systems”), “Industry” received the lowest mean score of 2, and “public bodies” gave the highest mean score of 4.2. The smallest differences across stakeholder clusters in mean score was given for recommendations 1 and 10. These scores ranged between 4.1 and 4.0, respectively, for “IPCO” and “Public bodies,” and 4.4 and 4.3 for “HCP” and “Research.”

Several of the Core Recommendations score highly for both questions 5 and 7 (as seen in Figures 3, 4). A comparison of the top 10 ranked core recommendations for questions 5 and 7 was undertaken to identify the overall top Core Recommendations. Five Core Recommendations were found to be in the top 10 of both questions 5 and 7: 9, 18, 17, 2, and 15.

#### Qualitative Analysis of Stakeholders' Responses

This section describes the analysis of stakeholders' responses to the Core Recommendations that were identified as most impactful by stakeholders and those the authors considered of greatest relevance to public health and readers of this journal. The analysis is derived from responses to questions 5 and 7. The analysis for the remainder of the recommendations can be found in the Supplementary Material, along with analysis of question 3 and 6.

Question 5: “Please identify the top three Core Recommendations (in order of importance) that you believe will deliver the most significant change in the regulatory system over the next 5 years and why.”

Question 7: “The following is to allow more detailed feedback on prioritization, which will also help shape the future application of resources. Your further input is therefore highly appreciated. Please choose for each row the option which most closely reflects your opinion. For areas outside your interest or experience, please leave blank. Should you wish to comment on any of the Core Recommendations (and their underlying actions) there is an option to do so.”

The results are described below in subsections per chosen Core Recommendation and, within these, by different stakeholder groups.

##### Support translation of advanced therapy medicinal products (ATMPs) into patient treatments

*Individual members of the public.* Two responses were received from individual members of the public. These held opposing views on the level of support that should be offered to ATMP development.

*Patient or consumer organizations.* Three responses received few additional proposed actions. However, it was suggested that EMA should make it a priority to ensure collaboration with patients, health care professionals, academia, and international partners in order to support translation of ATMPs into treatments. In addition to the development of payment models for ATMPs, EMA should highlight the issue of high prices for ATMPs, which hinder accessibility for patients.

*Health care professionals.* Both health care professionals who responded considered the ATMP field an essential part of innovation and one that offers ground-breaking new treatment opportunities.

*Other scientific organizations.* The four other scientific organizations that responded largely agreed that ATMPs have the potential to change the therapeutic landscape to the benefit of patients and supported the underlying actions proposed. Some believed that EMA should engage with other European authorities (heads of Medicines Agencies and EU-Innovation Network) and international regulatory agencies to foster global convergence on the regulatory requirements for ATMPs, as several aspects of the requirements remain the responsibility of EU national authorities. A number stated the need for appropriate regulatory tools and underscored that new or adapted regulatory paradigms should be able to deal with ATMPs.

*Health technology assessment bodies.* The four responses broadly supported the recommendation, and there was general support for most actions.

*Payers.* The two very similar responses from payers indicated that there was a need for a clear and universal definition of “unmet medical need” based on the public health perspective. It was suggested that EMA puts procedures in place to allow stakeholders' involvement throughout the ATMP lifecycle but ensuring that their participation remains impartial and transparent. Furthermore, as with the HTA bodies, they considered that the agency should create relevant processes to reevaluate or withdraw products which do not meet requirements post-approval.

*Pharmaceutical industry.* **Individual companies**

The recommendation and underlying actions were seen as valuable and important in the responses (*N* = 7). A few individual companies noted that the action to identify therapies that address unmet medical needs will require collaboration with multiple stakeholders, including industry, in order to provide a wide range of perspectives, specifically with regards to evidence generation. It was also suggested that HTA bodies, payers, and patients should be involved during the development of new treatments. It was anticipated that the EMA will play a key role in coordinating this recommendation and that EMA will further build on its previous work with other stakeholders in order to meet the needs of patients.

Furthermore, some noted the need for global convergence in the development of the ATMP regulatory framework as there are currently inconsistencies between national and European standards. Some felt that regulatory requirements should be flexible in order to be adaptable to more advanced technology and allow improvements in the manufacturing process of ATMPs.

**Trade associations**

Of the trade association's responses (*N* = 6), most supported the recommendation and some specifically focused on its underlying actions.

Several suggested that EMA should consider creating a regulatory framework supporting a multisource environment for ATMPs that will lead to a competitive market and thus more affordable therapies in the future in the EU. These regulatory pathways would be designed to stimulate evolution of the innovation life cycle and increase patient access to such therapies. Furthermore, for innovation to be converted into patient treatments, a more unified system was seen as vital with cross-fertilization between advancing developments and the regulatory environment.

A few responses highlighted the need for HTA involvement with respect to data requirements to avoid the inconsistent understanding of the requirements for market access for ATMPs. However, some believed multi-stakeholder discussions would also further improve how these products were assessed for efficacy/effectiveness compared with other treatments.

Responses indicated a need to ensure better cooperation between the European Commission and national agencies and authorities dealing with genetically modified organisms in member states and to ensure better alignment between EMA and national regulators. Continuous and effective dialogue with stakeholders would also allow for a more efficient development of products. A few contributions suggested that a specific platform for knowledge sharing could be developed and would facilitate the development of specific expertise and increase capacity across the EU regulatory network to assess ATMPs.

Some responses noted the interdependence with other recommendations in the strategy:

- Diversify and integrate the provision of regulatory advice along the development continuum;
- Contribute to HTA's preparedness and downstream decision making for innovative medicines.

Some responses also noted that ATMPs will eventually become off-patent. This would require the development of appropriate regulatory pathways for the future development and registration of ATMPs for which patents have expired, since the regulatory paradigm is different from that of new chemical or biological entities.

##### Foster innovation in clinical trials

*Patient or consumer organizations.* Patient or consumer organizations (*N* = 9) were consistent in advocating a focus on the strength, rigor, and representativeness of randomized controlled trials (RCTs) rather than innovative clinical trial design.

They requested that EMA ensure these RCTs include representative subgroups; demand comparative RCTs where possible; require that one of the two RCTs for approval be done by an independent party; pool resources across member states to allow meaningful, pragmatic RCTs responding to questions relevant to clinical practice, discourage surrogate end points where final outcomes are achievable within a reasonable time frame and without harm for trial participants.

They also expressed caution with regards to the use of Real World Data (RWD). They asked that EMA reflect on scope, checks and balances, and quality criteria for RWD. EMA should be cautious about using such data to establish clinical effectiveness due to a high level of confounding factors. Post-marketing evidence generation should focus on adverse drug reactions.

*Health care professionals organizations.* Four health care professionals organizations responded. There were no common themes which could be summarized.

*Academic researchers, European research infrastructures, other scientific organizations, and health care professionals.* Academia (academic researchers, *N* = 2; European research infrastructures *N* = 2, Other scientific organizations *N* = 9) were generally in favor of innovation in clinical trials, particularly regarding modeling, simulation, and extrapolation, and in trials on rare diseases. They felt scientific advice and *in silico* trials would facilitate such innovation. *In silico* trials, in particular, could help to reduce, refine, and partially replace real clinical trials. They could reduce the size and duration of trials by adding simulated patients that might fill gaps in the individual variability seen in “real” patients. They might also be able to determine and remove those patients who will not respond to the candidate biomedical product or improve safety through pharmacokinetic/pharmacodynamic multimorbidity modeling. Finally, RWD was recommended as a supportive data source for both *in silico* trials and RCTs. Several responses highlighted the interdependence of this Core Recommendation with others such as:

- Support developments in precision medicine, biomarkers, and “omics”
- Create an integrated evaluation pathway for the assessment of medical devices, *in vitro* diagnostics and borderline products.

*EU Regulatory partners/EU Institutions.* EU Regulatory partners (*N* = 6) requested that EMA monitors medicines continually, integrating additional information in clinical trials: from administration through pharmacological activity and pathophysiological modification to impact on disease/symptoms.

*Health technology assessment bodies and payers.* The HTA responses (*N* = 4) strongly supported recommendations to ensure that novel practices and procedures facilitate HTA acceptance and patient access.

Two payers considered that many modern trial designs are too flawed or at risk of bias to use in anything beyond exploratory clinical trials. They also requested that surrogate end points be allowed only when they have been validated as impacting clinically meaningful end points.

*Pharmaceutical industry.* Pharmaceutical companies (*N* = 17) and their trade associations (*N* = 7) supported innovation in clinical trials. They repeatedly requested that EMA organize dedicated multi-stakeholder collaborations (e.g., workshops, demonstration projects and pilot schemes) to raise awareness, share case studies, and identify best practice in innovative clinical trials.

On top of this, they requested that EMA create a forum to resolve alignment issues across National Competent Authorities, ethics committees, HTA bodies, and patients' organizations when considering acceptance of complex and/or seamless clinical trials. This could be complemented by a complex clinical trial strategic initiative including multiple stakeholders, to agree on standards that can be used as a basis for international harmonization, through the International Council for Harmonization (ICH). They stressed that the EU will lose attractiveness as a place to conduct clinical research if innovative clinical trials are not encouraged and EU stakeholders do not better align with the clinical trial pathway.

Some specific actions came up, for example, to further develop the EU's CT Information System (CTIS) to best accommodate complex clinical trials. The CTIS should be able to efficiently manage applications for, and the datasets arising from, complex clinical trials. Earlier timing of advice would facilitate parallel scientific advice from EMA and the U.S.'s Food and Drug Administration (FDA). Responses proposed that EMA consider harmonizing and developing guidance in biomarkers and end points, particularly digital biomarkers and patient reported outcomes, and looking into how the orphan medicines regulation will be adapted to tissue agnostic indications.

The responses linked this Core Recommendation with the implementation of these others:

- Develop the regulatory framework for emerging clinical data generation
- Support developments in precision medicine, biomarkers, and “omics”
- Create an integrated evaluation pathway for the assessment of medical devices, *in vitro* diagnostics and borderline products
- Reinforce patient relevance in evidence generation
- Optimize capabilities in modeling, simulation, and extrapolation
- Promote use of high-quality real-world data (RWD) in decision making

##### Reinforce patient relevance in evidence generation

*Patient or consumer organizations.* There was a unanimous call for greater and systematic patient engagement. With responses (*N* = 12) highlighting the added insights patients bring through living with disease and taking medicine. This involvement was requested to span the medicine development lifecycle, including clinical trial design with meaningful end points such as always incorporating quality of life (QoL) outcomes and patient-reported outcomes (PROs), as well as in the development of new and existing guidelines, where, again, PROs should be incorporated. It was stressed that these methodologies should be scientifically robust. They also requested EMA to develop a regulatory framework for digital clinical data generation and promote the use of high-quality RWD that includes patient data. Patients highlighted that implementing these measures would aid downstream decision makers such as HTAs; health care professionals and, ultimately, patients.

*Health care professionals' organizations.* Health care professionals (*N* = 6) viewed patient involvement as a priority, including in evidence generation. They advocated for ensuring that end points are patient relevant.

*Academic researcher, European research infrastructure and other scientific organizations.* Academic researchers (*N* = 2), a European research infrastructure, and other scientific organizations (*N* = 4) expressed a strong desire to reinforce patient engagement throughout the lifecycle of medicine development. In particular, they favored including PROs in evidence generation.

*Health technology assessment bodies and payers.* HTAs (*N* = 3) and payers (*N* = 2) welcomed the proposal for systematic inclusion of PROs and a health-related quality of life PRO measure (HRQoL) to implement in trials and bridge the gap with comparative assessment, so long as it is done with a common understanding between regulators, HTAs, and payers. Two suggested reviewing existing HRQoL measures before developing a new one and urged mindfulness regarding conflict of interest in patient engagement.

*Pharmaceutical industry.* **Individual companies**

Individual companies (*N* = 9) welcomed reinforcing patient engagement, seeing it as a reflection of their own efforts to do the same. They welcomed the inclusion of PROs into the benefit-risk assessment and requested that such data be included in the labeling. They also requested that the rigor and methods of inclusion of PROs should be collaborative, transparent, and harmonized across decision makers. The framework for digital data generation was seen as an enabler for this Core Recommendation.

**Trade associations**

Trade associations (*N* = 4) advocated that the agency go further with in-patient input, particularly for PROs. They suggested a systematic, whole-life cycle approach, with alignment across stakeholders, Europe-wide and globally. In developing tools for gathering patient input, they recommend a collaborative, multi-stakeholder approach, with clearly defined requirements and guidelines.

##### Promote use of high-quality real-world data (RWD) in decision making

*Patient or consumer organizations.* Patient or consumer organizations (*N* = 4) broadly acknowledged the added value of real-world data but sounded a note of caution about its use. They wanted clarity as to what could be considered high-quality real world data and when its use would be acceptable, advocating that it be seen as complementary to clinical trials.

*Health care professionals' organization.* Health care professionals (*N* = 4) viewed RWD as important for medicine evaluation, particularly post-approval. They stressed the need for an appropriate regulatory framework and platform to support collection and analysis of robust and relevant data, and to ensure appropriate governance and compliance with data protection requirements.

*Other scientific organizations.* Overall scientific organizations (*N* = 10) were positive toward RWD. They recognized its value in both pre- and post-approval settings for more closely reflecting real life and enabling the continuous review of the efficacy and safety of approved products. They requested that EMA produce guidance on what and when RWD is acceptable. They also requested that EMA demand transparency in observational studies.

*EU Regulatory partners/EU Institutions.* EU regulatory partners (*N* = 5) supported the use of RWD, so long as proper evaluation of its use was undertaken, and robust methodologies were developed. RWD should be able to be used for the full range of regulatory procedures and assessments, pre- and post-marketing.

*Health technology assessment bodies and payers.* HTA responses (*N* = 6) and payers (*N* = 2) had mixed views on RWD. Most welcomed its use post-approval; however, there was considerable doubt about its suitability for evidence generation pre-approval. They requested clarity over the regulatory acceptability of RWD methods and how these would impact on marketing authorizations: it should be made clear when evidence generation can be moved to the post-marketing phase and what the justification would be. They also requested the following be addressed:

- data standardization
- data quality
- registration in publicly accessible databases
- reproducibility
- data ownership
- validated statistical analyses
- transparency on conflicts of interests of interested parties
- data protection

Two responses requested that EMA refrain from using the term “real-world data,” preferring “observational data” and stating that RCTs should remain the gold standard.

*Pharmaceutical industry.* Individual companies (*N* = 23) and trade associations (*N* = 6) were near unanimous in their support for promoting high-quality RWD in decision making. They noted the growing potential of RWD, driven by the digitization of health care information and new analytical methods such as AI and modeling.

The responses explained that RWD will streamline evidence generation and assessment, particularly in rare diseases. They requested that EMA launch a strategic initiative to integrate RWD into medicines development. The initiative would involve pilots, capability building exercises, stakeholder engagement via workshops, and guidance. It could include both retrospective and prospective case studies and lead to the development of a regulatory training curriculum for RWD to build knowledge and capacity to regulate.

This initiative should provide clarity on the scope and regulatory uses of RWD and involve all relevant stakeholders, including at an international level. They requested the initiative clarify regulatory acceptability of RWD in areas such as label changes and the collection, quality, validation, transparency, security and privacy (including GDPR), analysis, financing, governance, and audit of sources of RWD.

There were also suggestions to develop or ensure the use of a common platform for RWD and for EMA, the European Commission, and HMA to link electronic health records into a resource. This initiative should build on ongoing work internationally, particularly from the FDA, and on EMA's own work on patient registries: the HMA/EMA Task Force on Big Data and the EMA's recent publication on “Use of patient disease registries for regulatory purposes—methodological and operational considerations” and include publicly available conclusions based on commercially confidential information. As an additional outcome, it would build regulatory experience in the area and so engender trust in RWD, and this would then permit international harmonization.

The responses suggested that the recommendation was interlinked with delivering the following Core Recommendations:

- Develop network competence and specialist collaborations to engage with big data
- Contribute to HTA's preparedness and downstream decision making for innovative medicines
- Reinforce patient relevance in evidence generation
- Exploit digital technology and artificial intelligence in decision making
- Foster innovation in clinical trials

##### Expand benefit-risk assessment and communication

*Patient or consumer organizations.* Responses from patient and consumer organizations (*N* = 11) reflected strong support for EMA to improve benefit-risk decisions and the way they are communicated; this should remain at the core of the EMA strategy. Regarding benefit-risk decision making, several stressed that EMA should carefully consider the use of accelerated and conditional approvals; the use of these approval types was described as justified in some situations but needed to be the exception as postponing reassurance about clinical value led to concern about putting patient safety at risk. Several felt that EMA should request comparative RCTs vs. standard therapy, using patient-relevant outcome end points whenever possible. This would reassure patients, HTA, and payers that a new treatment works better in comparison with alternative options (if any).

Several responses mentioned that benefit-risk assessment should include the evaluation of PROs, patient reported outcome measures, patient preferences, and individual patient data to reflect patient's actual needs and expectations. Several also stressed that EMA should ensure that submitted data answer clinically relevant questions and that regulatory decisions are guided by clearly defined, unmet public health needs. Responses highlighted that EMA should request high-quality post-marketing studies to confirm benefit and resolve uncertainties raised during the initial authorization, which was seen as especially important when medicines are authorized on less comprehensive data. Some responses also expressed the view that post-marketing evidence that does not confirm any benefit should inform the withdrawal of medicines. Similarly, several stressed that EMA should ensure pharmacovigilance activities remain a priority, again, especially in view of medicines arriving on the market before comprehensive data on their safety and efficacy can be gathered.

Regarding benefit-risk communication, EMA should ensure clear, sufficient, and transparent communication of the benefits and risks, specifically of products approved via accelerated procedures and conditional marketing authorization; patients, HCPs, and prescribers should be fully aware of the benefits and risks and how they compare in order to make informed treatment decisions.

*Other scientific organizations.* All three responses were very supportive and suggested some specific actions. They emphasized the need to build on existing good practice guidance (such as that issued by ISPOR and the FDA, as well as the results of IMI PREFER).

*EU Regulatory partners/EU Institutions.* All responses (*N* = 10) stressed the importance of these actions, stating that benefit-risk assessment is the core competency in regulation. Improving the consistency, transparency, and predictability of benefit-risk decisions was found to be necessary to ensure that they do not become less meaningful for subsequent decision makers and patients. Many recommended establishing a process for continuous monitoring of benefits and risks after initial approval incorporating RWE, thereby moving away from single-time point approval.

*Health technology assessment bodies.* Of the five responses, many welcomed better communication and/or collaboration with payers and specifically welcomed deepened discussions on unmet medical needs, severity of disease, existing treatment options, suitable comparators and outcomes comparison vs. placebo/active-control (including size in effectiveness in absolute terms), and patient perspectives.

*Payers.* Payers (*N* = 5) largely echoed HTA bodies' feedback. All payers strongly supported better communication and/or collaboration amongst payers and HTAs. They specifically welcomed deepened discussions on unmet medical needs, severity of disease, existing treatment options, suitable comparators and outcomes comparison vs. placebo/active-control (including size in effectiveness in absolute terms), and patient perspectives.

Regarding communication, several stressed that EMA should publicly explain its decisions, provide insights into the benefit-risk balance, and warn against possible harm, so that patients and downstream decision makers are clearly informed about the reasons behind decisions and side effects. Two stated the need (i) for more detailed descriptions of remaining uncertainties of the benefit-risk assessment and (ii) to clarify that HTA bodies/payers and EMA have different responsibilities and methodological requirements.

Regarding patient preferences, a number stated that the incorporation of patient preferences should happen in a methodologically sound, transparent and impartial way with clear rules for conflict of interest. Some responses mentioned that actions regarding preferences should consider the methodological challenges of eliciting patient preferences: for example, preference studies are too often misleading as preferences change with experience with illness and become less precise with increasing complexity of decisions, and such studies tend not to elucidate the whole picture.

*Pharmaceutical industry.* Individual company responses (*N* = 5) overall supported actions related toward expanding benefit-risk assessment and communication via the incorporation of patient preference data. They expressed support for improving communication, particularly with HTA bodies. However, two responses diverged as to whether the capability to analyze Individual Patient Data was the best use of regulatory resources.

Overall, trade associations (*N* = 5) supported the actions related to expanding benefit-risk assessment and communication. Two stressed the importance of having support and alignment among all concerned stakeholders on actions related to methods for structuring benefit-risk assessment, communication, and patient preferences.

##### Contribute to HTA's preparedness and downstream decision making for innovative medicines

*Patient or consumer organizations.* Of the ten responses, many agreed that EMA should engage in early discussions with HTA bodies to align (clinical) evidence requirements to close (clinical) evidence gap between HTA and regulatory requirements; several stated that a lack of alignment currently impedes or slows down patient access. They considered that this required close collaboration with payers and HTA bodies, as well as the involvement of patients and health care professionals. While recognizing the different roles of regulators, HTA bodies, and payers, a few responses proposed that regulatory requirements should be adapted to meet HTA, payer, and society requirements, e.g., by making it a regulatory requirement that added therapeutic value be demonstrated, with regulatory guidelines mandating the submission of comparative trial data against standard treatment.

A number of responses highlighted actions for cooperation with HTA bodies, for example, by inviting HTA experts to Committee for Medicinal Products for Human Use (CHMP) discussions, and the need to anticipate divergences and reimbursement challenges when regulatory concepts do not fit the reimbursement setting. Regulatory concepts for discussion mentioned in this context were surrogate end points (when the relationship between the end point and clinical outcome has not been completely established), conditional approval, the population to benefit, significant benefit for orphan medicinal products (mentioned multiple times), and unmet needs.

Various responses highlighted the idea that parallel EMA/HTA scientific advice should be strengthened to reduce the risk of inadequate information provided to EMA/HTA at time of evaluation. The European Network for Health Technology Assessment (EUnetHTA) could be used as a platform to exchange information between CHMP and HTA and HTA assessors allowed to have this information in parallel to CHMP evaluation.

*Health care professionals' organizations.* Health care professionals' organizations (*N* = 4) highlighted that EMA should enhance discussions with HTAs regarding HTA guidance and methodologies for evidence generation and review. Some also mentioned that EMA could contribute to identification of priorities for HTAs.

An additional suggestion was that a robust and effective framework was needed for collaborative EU-level HTA assessment in order to streamline regulatory procedures, avoid duplication, shorten time for decision making, and make the best use of public and private human and financial resources.

*Academic researchers.* Only two academics responded to this question. Their responses showed strong support for the proposed actions aiming to strengthen collaboration and alignment of evidence requirements between EMA and HTA bodies. The two researchers proposed various detailed actions toward this goal, notably:

- Contributing to the development a of core outcome sets (COS) together with HTA bodies for use “throughout the ecosystem” by regulatory and HTA reimbursement assessments and decisions; it was proposed that research using these outcomes can be compared and combined and that all studies provide usable information;
- Collaborating with HTA bodies on post-authorization evidence requirements and introduce EU clinical registries post-authorization in addition to existing managed entry agreements.
- Anticipating that registries will require significant investment in registry design, operating data systems, and training and licensing; the cost of running the registries should be factored in HTA evaluations and discussed on the distribution of costs between the payer and manufacturers.
- HTA bodies could stipulate a resource impact assessment applying the annuity and payment by performance models. This criterion would serve as a tool to predict future expenditure and identify the best reimbursement model early on.

*Health care professionals.* The responses (*N* = 2) suggested aligning this priority with the adoption and implementation of the legislative proposal on HTA collaboration.

*Other scientific organizations.* Responses (*N* = 3) pointed toward actions to collaborate with, and leverage knowledge and experience from, HTA bodies. Many noted the value of using an EU-based approach, e.g., via EUnetHTA.

*Health technology assessment bodies.* Responses (*N* = 5) supported actions to ensure collaboration between regulatory agencies and HTA bodies.

*Payers.* Payers (*N* = 4) asked that EMA ensure that requirements for HTA/payer processes are already integrated in the pre-authorization phase; trial designs should reflect the requirements of HTA assessments. Although incorporation of evidence needed by payers and HTA into development plans was described to be indispensable, some stated that fulfillment of HTA requirements should be essential for achieving marketing authorization while others commented that HTA and regulators have different responsibilities and therefore rightfully ask different questions.

Similarly, differences between HTA and EMA assessments were seen as justified and not hindering better cooperation. However, responses asked to better explain these differences in the public domain. Several also asked EMA to clarify what “contributing to HTA priority setting” is supposed to mean. It was highlighted that target parameters should be defined when monitoring the impact of decision makers' engagement. Furthermore, several underlined that while discussion often focuses on access alone, in reality, the triangle of access, affordability, and added benefit was stated to be relevant.

*Pharmaceutical industry.* **Individual companies**

Individual company responses (*N* = 9) all supported continuing collaboration with HTA bodies. Streamlining evidence requirements between regulators and HTA bodies was seen as necessary for ensuring timely access to medicines. Many stated that EMA should ensure broader stakeholder agreement and alignment early in medicines development on the data and evidence to be generated in order not to delay regulatory approval and patient access. Increased focus on opportunities for early dialogue/parallel consultation with all stakeholders was also welcomed. Two responses were more cautious.

**Trade associations**

Responses (*N* = 4) stated that actions should aim to ensure evidence pertinent to regulators, HTA, payer needs, and patients is defined early in medicine development and by incorporating input early from all stakeholders (including HTA, payers, and patients) in medicines development and evidence requirements. This was especially described to be necessary for ATMPs and other areas where innovation puts pressure on the EU system, e.g., personalized medicine and medicine for rare diseases. Multiple responses also underlined the necessity of actions needed to increase transparency; EMA should make documents publicly available explaining why and how decisions during the approval process were made, e.g., why the agency accepted the trial design, the end points for approval, why a given duration of trial was acceptable. This would assist HTAs and payers reviewing submissions at a later date. Two participants described that EMA should help ensure that any limitations in data presented at marketing authorization are recognized early, together with proposals to mitigate any limitations to enable access. This was stated to be important, specifically with respect to rare diseases that often have small and heterogeneous clinical trial populations; also, here, EMA should ensure input from all stakeholders is incorporated to determine how limited data in some patient populations can be managed to improve patient access.

##### Bridge from evaluation to access through collaboration with payers

*Patient or consumer organizations.* Of the responses (*N* = 8), many patient and consumer organizations were positive about adapting regulatory requirements for premarket evidence so as to meet the demands of HTA, payers, and society.

*Health care professional and health care professionals' organizations.* The two suggestions received from HCP organizations were to promote a transparent mechanism that allows payers to recognize the value of new therapies (it was noted these are often not recognized by payers and therefore not reimbursed or used), and to clarify the treatment-eligible patient population included in the labeling and its scientific rationale.

*Other scientific organizations.* Of the responses (*N* = 7), some expressed support for the continued development of collaboration with HTA bodies, payers, and other stakeholders across medicines' life cycle. Others, however, felt that EMA should consider the distinct roles of HTA and payers; payers consider not only the HTA evaluation, but also national, economic, political, and other public policy considerations (e.g., health priorities) in making their decision. Whereas, the added value of EMA-HTA collaboration was said to be clear as it focuses on data assessment, in which the expertise of EMA was welcome, the benefit of EMA-payer collaboration was seen as more limited (such as for horizon scanning).

*EU Regulatory partners/EU Institutions.* The two responses suggested specific actions related to the need for EMA to exchange more information with HTA/payers to increase their timely preparedness for evaluating medicines for reimbursement.

*Payers.* Payers (*N* = 4) supported the action of creating a single platform for interaction on evidence generation plans so that these could satisfy EMA and payer decision making; the needs of payers had to be reflected early on in the approval process. The EMRN was asked to reflect on establishing a permanent working structure between EMA and payers with relevant objectives, planning and responsibilities.

*Pharmaceutical industry.* **Individual companies**

Mirroring comments from other stakeholder groups, many of the seven responses from the pharmaceutical industry supported cooperation, including a single platform to enable one evidence generation plan and providing the rationale for authorizing a particular patient population. Responses supported actions to ensure that development plans consider all elements necessary not only to demonstrate efficacy and safety but also to comply with downstream requirements. However, echoing comments from scientific organizations, many considered collaboration with payers to be more complex than collaboration with HTA; although payer decisions are informed by HTA assessment, the criteria for payer decisions were described to be very different from the clinical assessment undertaken by the EMA.

It was felt that EMA-payer collaboration would be limited by the complexity of the payer infrastructure across Europe; streamlining with national payer decisions could potentially lead to complexity and delay in the regulatory system. Similarly, several responses suggested that initiatives related to payer decision making should be undertaken by other agencies at the EC and national level as opposed to EMA; it was reiterated that EMA should consider that these actions extend the remit of EMA beyond scientific evaluation into political decision making, which was described as a member state government competence.

**Trade associations**

Of the five responses, many stressed that EMA should leverage payer collaboration to gain insight into their perspectives on unmet needs and priorities; early engagement in turn helps to prepare payers for potential major impacts from breakthrough innovation.

However, in line with comments from other stakeholder groups, several stated that EMA should consider the need to maintain the distinctiveness of regulatory processes and pricing determinations, and for regulators to keep their scientific focus; initiatives to address pricing and reimbursement decision making should be undertaken by other agencies at the European Commission and national levels.

##### Implement EMA's health threats plan, ring-fence resources and refine preparedness approaches

This Core Recommendation received too few comments to generate detailed summaries.

*Payers.* The two payers supported the initiative.

*Pharmaceutical industry.* The two industry responses and one trade association provided different suggestions for EMA's health threat activities.

### Responses to the Veterinary Section of the RSS

#### Quantitative Analysis

Of the total of 154 survey responses, 7 replied to the veterinary part only and 17 to both, resulting in a total of 24 responses of veterinary relevance.

Only 1 response was received for the veterinary part from an individual member of the public, represented as cluster 1. For health care professionals, 6 responded to the veterinary questionnaire, of which 2 were veterinarians and 4 were responses from organizations representing health care professionals. From the research sector, there were 6 responses to the veterinary part, including 1 learned society; 1 farming and animal owner organization; 1 individual academic researcher; and 3 other scientific organization.

From public bodies, a total of 6 responses were received (6 of which responded to the veterinary part), including 3 from medicines regulatory agencies, 2 from other EU public bodies and 1 from the European Commission.

From industry, 5 responses were received for the veterinary part, including 2 from the main trade associations (spanning all industry types), 2 small and medium enterprises (SMEs), and 1 individual company (non-SME).

A limited number of responses were received for the veterinary questionnaire; therefore, the results should be interpreted with caution.

**Figure 5:**
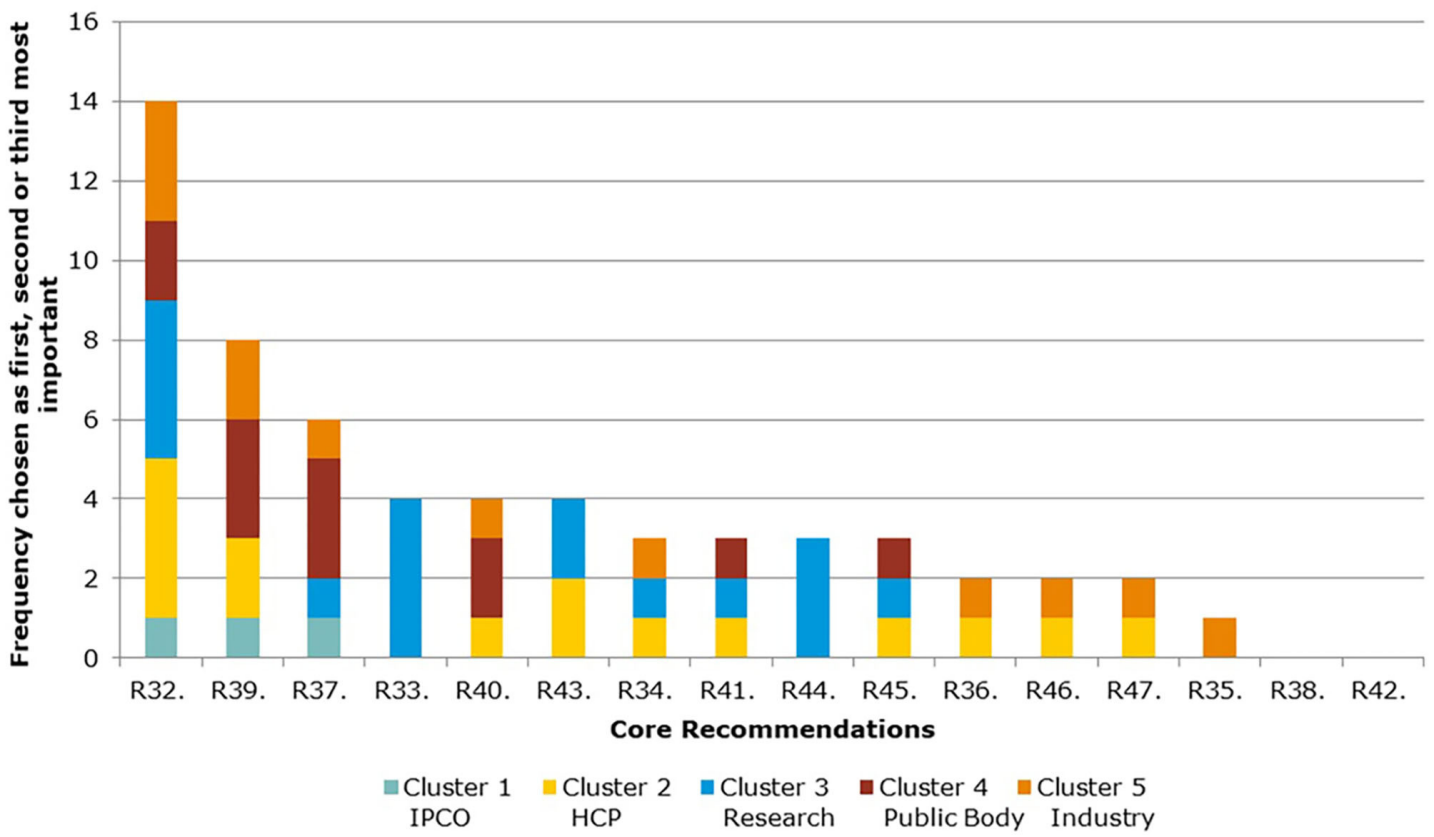
Aggregate ranking of Core Recommendations (question 5, Veterinary).

Figure 5 and Table 4 show the ranking of Core Recommendations by the frequency stakeholders identified them as one of their top three to deliver “the most significant change in the regulatory system over the next 5 years” (question 5). The figure also illustrates the total scores per stakeholder cluster for each Core Recommendation. Core Recommendation 32 received the highest score from 14 stakeholders. Two Core Recommendations, 38 and 42, were not chosen as a first, second, or third choice by any cluster. Core Recommendations 33 and 44 were only selected by cluster 3 and no other.

**Table 4:** Aggregate ranking of Core Recommendations (question 5, veterinary medicine).

**Number**	**Core Recommendations**	**Total^a^**
32	Transform the regulatory framework for innovative veterinary medicines	14
39	Develop new approaches to improve the benefit-risk assessment of veterinary medicinal products	8
37	Collaborate with stakeholders to modernize veterinary pharmacoepidemiology and pharmacovigilance	6
33	Reinforce and further embed application of the 3Rs principles	4
40	Continue to promote the responsible use of antimicrobials and their alternatives	4
43	Promote and support development of veterinary vaccines	4
34	Facilitate implementation of novel manufacturing models	3
41	Coordinate Network activities to improve data collection on antimicrobial use in animals	3
44	Develop network-led partnerships with academia to undertake fundamental research in strategic areas of regulatory science	3
45	Leverage collaborations between academia and network scientists to address rapidly emerging regulatory science research questions	3
36	Apply the latest scientific principles to the assessment of the safety of residues of veterinary medicines	2
46	Identify and enable access to the best expertise across Europe and internationally	2
47	Disseminate and exchange knowledge, expertise, and innovation across the network and to its stakeholders	2
35	Update Environmental Risk Assessments in line with the latest scientific knowledge	1
38	Develop new and improved communication and engagement channels and methods to reach out to stakeholders	0
42	Engage with stakeholders to minimize the risks of anti-parasitic resistance	0

a *Total times chosen as first, second, or third most important*.

**Figure 6:**
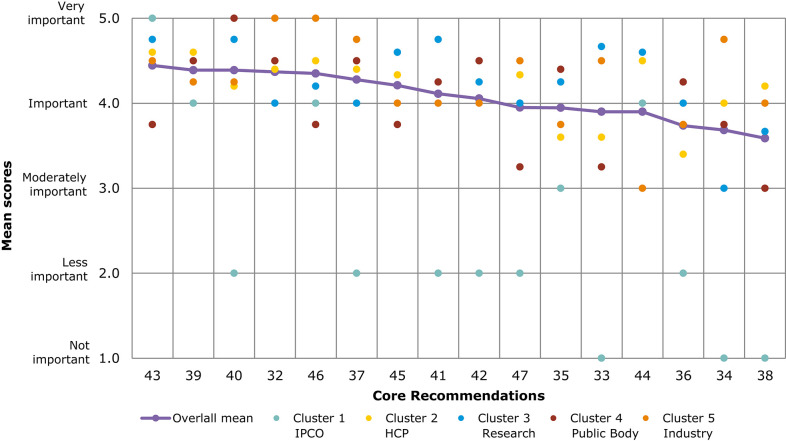
Overall comparison of all Core Recommendations by mean score across stakeholder clusters (question 7, Veterinary). Overall means were calculated based on the individual scores given by participants who rated each Core Recommendation on a scale from 1 to 5: (1) not important; (2) less important; (3) moderately important; (4) important; and (5) very important.

Figure 6 and Table 5 present a comparison of overall mean scores per Core Recommendation. Due to the limited response number for the veterinary questionnaire, with an average of 17 ratings per Core Recommendation, the results should be interpreted with caution. The overall mean score per Core Recommendation does not fall below 3 (moderately important). Figure 6 shows the mean scores per stakeholder cluster for each Core Recommendation. The mean scores of cluster 1 (IPCO) differs significantly compared with the mean scores of other clusters; it is based only on one respondent. Five Core Recommendations received the highest overall mean score of 4.4 (32, 43, 39, 40, and 46). “IPCO” did not provide feedback for recommendation 45 as it was seen outside of their area of interest or experience. In comparison, cluster 3 ranked the recommendation highly with a mean score of 4.6 (important and very important).

**Table 5:** Overall comparison of all Core Recommendations by mean score (question 7, veterinary medicine).

**Number**	**Core Recommendations**	**Mean score**
43	Promote and support development of veterinary vaccines	4.4
39	Develop new approaches to improve the benefit-risk assessment of veterinary medicinal products	4.4
40	Continue to promote the responsible use of antimicrobials and their alternatives	4.4
32	Transform the regulatory framework for innovative veterinary medicines	4.4
46	Identify and enable access to the best expertise across Europe and internationally	4.4
37	Collaborate with stakeholders to modernize veterinary pharmacoepidemiology and pharmacovigilance	4.3
45	Leverage collaborations between academia and network scientists to address rapidly emerging regulatory science research questions	4.2
41	Coordinate Network activities to improve data collection on antimicrobial use in animals	4.1
42	Engage with stakeholders to minimize the risks of anti-parasitic resistance	4.1
47	Disseminate and exchange knowledge, expertise, and innovation across the network and to its stakeholders	4.0
35	Update Environmental Risk Assessments in line with the latest scientific knowledge	3.9
33	Reinforce and further embed application of the 3Rs principles	3.9
44	Develop network-led partnerships with academia to undertake fundamental research in strategic areas of regulatory science	3.9
36	Apply the latest scientific principles to the assessment of the safety of residues of veterinary medicines	3.7
34	Facilitate implementation of novel manufacturing models	3.7
38	Develop new and improved communication and engagement channels and methods to reach out to stakeholders	3.6

*Overall means were calculated based on the individual scores given by participants who rated each Core Recommendation on a scale from 1 to 5: (1) not important; (2) less important; (3) moderately important; (4) important; (5) very important*.

As seen in Figures 5, 6, several of the Core Recommendations are highly scored for both Question 5 and Question 7. By comparing the top 10 ranked Core Recommendations for Question 5 and Question 7, seven Core Recommendations (numbers 32, 39, 40, 43, 45, 37, and 41) were found to be in the top 10 for both questions.

#### Qualitative Analysis

The qualitative analysis section focuses on the Core Recommendations that were identified as most impactful by the authors or which the authors considered of greatest relevance for animal health and that of readers. A more comprehensive analysis for all recommendations can be found in the Supplementary Material. The analysis is derived from responses to questions 5 and 7.

The qualitative analysis section looks into all the input received from stakeholders. Due to the limited overall number of responses to the veterinary questionnaire, the summaries are reported for each Core Recommendation, rather than for each stakeholder group. The results are ordered into subsections for each of the chosen Core Recommendations.

##### Transform the regulatory framework for innovative veterinary medicines

The responses (1 individual member of the public, 1 veterinarian, 1 farming and animal owner organization, 2 health care professional organizations, 2 regulatory partners/EU institutions, 2 other scientific organizations, 1 SME and 4 trade associations) were supportive of the Core Recommendation. Respondents saw opportunities for immunotherapies. However, they cautioned that the veterinary sector is different from the human medicines sector, and these differences need to be considered. They were also at pains to emphasize the need for a flexible and rapid system to avoid innovation being dependent on changes in the core regulations.

##### Reinforce and further embed application of the 3Rs principles

The responses (1 academic researcher, 2 veterinarians, 2 EU regulatory partners/EU institutions, 2 other scientific organizations and 1 trade association) were generally supportive of the 3R principles: replacement, reduction, and refinement of animal testing. They stressed the need to internationally harmonize standards.

##### Develop new approaches to improve the benefit-risk assessment of veterinary medicinal products

The responses (1 veterinarian, 2 health care professional organizations, 4 EU regulatory partners/EU institutions, 1 SME and 2 trade associations) generally supported the Core Recommendation, particularly when considering veterinary vaccines and residues. Responses also requested that new methods for benefit-risk assessment should be flexible and should avoid being too risk averse.

##### Collaborate with stakeholders to modernize veterinary pharmacoepidemiology and pharmacovigilance

The responses (1 individual member of the public, 1 veterinarian, 3 EU regulatory partners/EU institutions and 2 trade associations) mainly viewed the modernization of veterinary pharmacoepidemiology and pharmacovigilance as important, welcoming a new system for pharmacovigilance.

##### Continue to promote the responsible use of antimicrobials and their alternatives

The responses (1 veterinarian, 4 health care professional organizations, 3 EU Regulatory partners/EU Institutions and 3 Trade associations) were generally supportive of the Core Recommendation and taking a strong One Health approach, which is the principle that recognizes that human and animal health are interconnected, that diseases are transmitted from humans to animals and vice versa and must therefore be tackled in both (12). It also encompasses the environment, another link between humans and animals and likewise a potential source of new resistant microorganisms. They requested the recommendation include examples of alternatives to antimicrobials. However, they cautioned that enough treatment options should remain available.

##### Promote and support development of veterinary vaccines

The responses (1 EU Regulatory partner / EU Institution, 1 Other scientific organization and 3 Trade associations) supported the Core Recommendation, in particular highlighting its importance for reducing use of antimicrobials in animals.

## Overall Results

The qualitative and quantitative analysis for all the survey questions (see Supplementary Material for full results) indicate that participants recognized the relevance of the regulatory science areas and the recommendations proposed in EMA's strategy. As seen from the quantitative analysis, nearly all Core Recommendations proposed were deemed important and of high priority (Figures 4, 6). Stakeholders were generally supportive of innovation in regulatory science in these areas, so long as they proceed through multi-stakeholder consultation, clear communication, and efforts toward harmonization across Europe and internationally. To this end, respondents stated that consultation should be less *ad hoc* and more systematic: co-decision over consultations. Similarly, there were overarching requests for greater transparency and “Open Science.”

Although, most areas were considered relevant and important, some received mixed views. The perceived expansion of EMA's role from a simple gatekeeper to enabling access was questioned by some in relation to early access schemes and the use of RWD. Mixed views were also expressed on the ambitious nature of the strategy, with respondents requesting more information on how these recommendations will be implemented, particularly regarding the implications and role for the wider EMRN and resources.

The two areas repeatedly identified as underrepresented or missing were pharmacovigilance on the human side and the need for a “One Health” approach, interlinking the human and veterinary fields. This interlinkage was seen as particularly important for 3Rs (Replacement, Reduction and Refinement of animal testing) and emerging health threats. On the veterinary side, respondents felt that more could be done in general for emerging health threats, in particular for Antimicrobial resistance (AMR), as well as for 3Rs.

## Discussion

### Overall Views

The recognition by stakeholders that the regulatory science areas and the recommendations proposed in EMA's strategy are relevant is in part a testament to stakeholders' own input throughout the drafting process. The support given to innovation in many of these regulatory science areas was contingent on regulators' providing direction and clarity. This direction was in turn shaped by multi-stakeholder consultation, alongside efforts toward harmonization across Europe and internationally. These requests for multi-stakeholder consultation are well-corroborated by the positive feedback received on the consultative nature of this process. Similarly, the consultative approach by EMA echoes the overarching requests for greater transparency and “Open Science,” as seen in other Core Recommendations, along with further patient engagement. From an agency perspective, the importance of consultation is shared, and this has ensured cycles of input and thorough peer review that has reduced bias and improved the quality of the RSS.

The areas that received mixed views related in particular to those where there was a perceived shift in EMA's role from gatekeeper to enabling access to medicine. Indeed, EMA's vision, as laid down in the RSS, relies on enabling innovation as a cornerstone of its activities. This is key for success in anticipating regulatory challenges to achieve a reasonable balance between translating scientific and technological progress into medicines as well as a rigorous scientific evaluation. In our view, the fact that regulators need to be at the forefront of innovation does not preclude, but rather strengthens, the essential public-health safeguarding role of gatekeeper.

The mixed views across stakeholders mainly arose in areas of regulatory science where there may be more uncertainty, such as conditional marketing authorization and the use of RWD. This highlights the need for reflection on further engagement and communication activities to stakeholders to convey that adoption of novel methodologies or regulatory tools does not imply a relaxation in scientific and regulatory standards. On the contrary, they will add to existing tools and stringent approaches currently used. RWD, for example, offers the potential to complement knowledge of a medicine's safety profile in the post-marketing phase, during clinical use (3). On the other hand, for certain unmet medical needs or during public health emergencies, conditional marketing authorization provides access to new medicines on the basis of less information than normally expected—but always on the basis of a positive benefit-risk evaluation.

The calls for a more “One Health” approach to the strategy reflect the opinion of stakeholders that regulatory science issues should be considered interlinked at a strategic level, rather than left to be developed in silos. This point can be extrapolated to the international level where stakeholders bemoan the lack of harmonization (yet expound the best practice of various regulators). On the lack of a dedicated pharmacovigilance section, the activities relevant to safety and risk management of medicines are incorporated across the document. Although the updated strategy does make better reference to pharmacovigilance—as a core activity of EMA to deliver its mission—pharmacovigilance is better reflected in the upcoming EU Network Strategy (EMRN), the overarching scientific, administrative, and legal priority areas, goals, and actions to advance the public health mission of the regulatory network in the EU.

Despite mixed views, the fact that there were very few areas identified as missing suggests that, in the eyes of stakeholders at least, this presents a reasonable scenario for developments in the coming 5 years.

### Core Recommendations in the Human Section

#### Support Translation of Advanced Therapy Medicinal Products (Cell, Genes, and Tissue-Based Products) Into Patient Treatments

Stakeholders welcomed further regulatory support for ATMPs. There was agreement among stakeholders, including industry, on the need for enhanced collaboration and input into ATMP development from patients and HCPs, and regulators, HTAs, and payers. They saw EMA as an organizer for this collaboration, building on its experience of bringing stakeholders together. This collaboration should aim toward global harmonization, which is understandable given the novelty and therefore limited agreed standards. However, a trade-off exists between harmonizing standards and including flexibility in these fast-moving areas, and both were requested.

Responses requested a clearer definition, across stakeholders, of unmet medical need. Currently, definitions vary in their inclusion of individual disease severity, available treatments, and patient population size. To alleviate this unmet medical need, the development of a multisource ATMP regulatory framework was suggested as this could enhance the number of providers, improve competition, and, therefore, improve patient access.

Such a strong call for collaboration among stakeholders may reflect the fragmentation of European medicines development landscape, particularly in novel fields such as ATMPs.

#### Foster Innovation in Clinical Trials

Patients, HTAs, and Payers were hesitant to endorse the recommendation for innovation in clinical trials. Instead, they favored a strengthening of the rigor of existing RCT designs. Conversely, responses from academic and industry stakeholders were in favor of innovation such as *in silico* modeling simulation and extrapolation. Again, in this novel area, it is understandable that patients and decision makers are rightly cautious about supposed risks, while those conducting research may be attracted by the supposed benefits of innovation in clinical trials. It therefore makes sense to recommend a platform for multi-stakeholder dialogue about their use; for example, on approaches to trial conduct and analysis that are fully validated and meet the needs of all patients, including neglected populations, such as pregnant women, the elderly, and those of diverse ethnicity.

#### Reinforce Patient Relevance in Evidence Generation

Patient relevance was an area of remarkable harmony, with all stakeholder groups calling for greater and systematic patient engagement. In particular, the systematic inclusion of PROs and other patient relevant end points was advocated for, and industry and patients were aligned in calling for a cross–life cycle, multi-stakeholder approach. This can be interpreted as a sign that the idea of patient relevance has spread across stakeholder groups and that moves toward a systematic inclusion of relevant patient end points in medicines development would be welcomed and should be pursued by all stakeholders, including drug developers, HTA, and regulatory decision makers.

#### Promote Use of High-Quality Real World Data (RWD) in Decision Making

While there were differing views among stakeholder groups about the way RWD should be incorporated into decision making, they were limited to the use of RWD in replacement to clinical trials. Patient or consumer organizations and HTAs' and payers' responses were wary of RWD's suitability for routine use as pre-approval evidence. This was in contrast to industry stakeholders, who were strongly in favor and went much further in discussing how to clarify requirements for RWD use. This difference in views may be due to the relative lack of experience and standards for using RWD pre-approval, hence the hesitancy from patients and HTAs and payers. It could, therefore, be seen as reassuring that such standards were requested across all stakeholder types. Indeed, RWD uptake will be dependent on whether these standards satisfy the concerns and needs of stakeholders. The above platform on innovative clinical trial design would be well placed to build consensus for these standards.

#### Expand Benefit-Risk Assessment and Communication

Despite all stakeholder groups welcoming the recommendation as a whole, there were differing views on the actions necessary to implement this recommendation. Patient or consumer organizations and other EU regulatory partners suggested expanding the benefit-risk assessment into continuous monitoring through enhancing the use of post-licensing evidence generation. Such a suggestion would require a trade-off between the validation of positive benefit-risk decisions and clinical relevance in real world settings, with the additional burden on developers and health care systems to generate this evidence. Patient or consumer organizations also strongly advocated for the inclusion of patient data, to enhance patient relevance. This was a view also shared by payers, who, stressing the need for rigor, could use this to inform pricing priorities and negotiations.

Payers, along with industry, welcomed increased communication of the rationale behind benefit-risk assessment to better inform and harmonize decisions downstream. This could reduce duplication of assessment and increase predictability for developers as well as better inform HCPs and patients. Payers and patient or consumer organizations' responses reflected a clear need to improve the communication of EMA's benefit-risk assessment.

#### Contribute to HTAs' Preparedness and Downstream Decision Making for Innovative Medicines; Bridge From Evaluation to Access Through Collaboration With Payers

The need for increased interaction between EMA, HTAs, and payers was shared across stakeholder groups, but there were differences on the extent and methods of this interaction. Payers and HTA bodies, along with patients, expressed the greatest desire for increased collaboration. Patients and payers highlighted the idea that, in order to improve access, both affordability and added benefit need to be considered in early discussions with developers. On the other hand, other scientific organizations said that EMA should not be involved in discussions regarding national criteria for reimbursement, such as price and budget impact. For this reason, some were hesitant regarding cooperation with payers. An area of cooperation, highlighted not only by payers and HTA bodies but by all stakeholder groups was in the alignment of evidence requirements. It was suggested by industry and payers to create a single platform for interaction on evidence generation plans so that the needs of all stakeholders could be considered, while the remits remain separate. Similarly, stakeholders asked for increased communication on a decision-making rationale that would serve to help inform decision making for downstream stakeholders such as HTAs and payers.

#### Addressing Emerging Health Threats and Availability/Therapeutic Challenges

The fact that this Core Recommendation received too few comments to generate detailed summaries and was rated as one of the least important is a concerning observation given the risks that public health threats can pose, as evidenced by the COVID-19 pandemic. This lower level of interest may be due to the perception that regulators had taken sufficient preparedness measures, or that stakeholders who find this an important recommendation did not participate in the survey. It will be interesting to see, therefore, whether activity in this area will increase following the COVID-19 pandemic, and the authors hope that the comments received will be a useful resource in this regard (13).

### Core Recommendations for the Veterinary Section

Before discussing the highest scoring core recommendation for the veterinary regulatory strategy, it is important to note that the consultation took place shortly after the publication of a new legislative basis for the authorization of veterinary medicines, Regulation (EU) 2019/6 of the European Parliament and of the Council of December 11, 2018, on veterinary medicinal products and repealing Directive 2001/82/EC, which will take effect in January 2022. This important legal text requires changes to the current approach to regulating veterinary medicines. It must be assumed that at least for some stakeholder groups the preparation for implementing the new provisions will have influenced the responses.

#### Transform the Regulatory Framework for Innovative Veterinary Medicines

Stakeholders agreed on the need to transform the regulatory framework for innovative veterinary medicines. This is a growing area that challenges the authorization of veterinary medicines and requires a new framework for some novel medicines for use in animals. Indeed, innovation is one of the driving forces of the Regulation (EU) 2019/6, and its implementation will help current regulatory paradigms and guidelines adapt to innovation. For this to occur, however, regulatory expertise will also need to be fostered.

#### Reinforce and Further Embed Application of the 3Rs Principles

The comments received reinforced the 3Rs principles, which are of relevance for human and veterinary medicine. These principles state that animal testing should be replaced, reduced, and refined to minimize the pain and distress of the animals used. Novel approaches in line with these principles—e.g., microfluidics and *in silico* modeling—are the subject of ongoing research and have the potential to benefit medicine development and support early efficacy studies, as well as improving the predictive ability of testing systems (10). However, stakeholders pointed out that international harmonization is critical to ensure that these advances gain backing globally to create consistent incentives for their application.

#### Develop New Approaches to Improve the Benefit-Risk Assessment of Veterinary Medicinal Products

Improved benefit-risk methods would ensure that innovation is accommodated and better evidence is generated to underpin regulatory decisions that will benefit animals. The support expressed within the responses is therefore unsurprising. However, it was given a caveat with the need for flexibility and a risk-based approach to their application. Flexibility could prevent changes to the benefit-risk assessment becoming unfit when future innovations and evidence generation arrive. Therefore, there seems a need to consider approaches to the benefit-risk assessment that are either future-proofed or flexible.

#### Collaborate With Stakeholders to Modernize Veterinary Pharmacoepidemiology and Pharmacovigilance

The need to collaborate with stakeholders to modernize veterinary pharmacoepidemiology and pharmacovigilance was reinforced in the comments received. The move to continuous monitoring through means such as signal detection will only be a success with this collaboration. For example, underreporting of suspected adverse effects in the veterinary domain is well-documented, particularly for food-producing animals. This situation has not improved despite specialization within the veterinary profession. Stakeholder collaboration will be required to explore whether and how use can be made of new digital technologies and communication channels (e.g., social media) in increasing reporting rates and improving the communication of pharmacovigilance outputs to veterinary health professionals and the public (10). The legal basis of pharmacovigilance for veterinary medicines has changed significantly with Regulation (EU) 2019/6, which explains the stakeholder interest in this recommendation.

#### Continue to Promote the Responsible Use of Antimicrobials and Their Alternatives

The support expressed by stakeholders for the responsible use of antimicrobials and their alternatives was reinforced through calling for a holistic, one-health approach to tackling antimicrobial resistance. This is positive, as Regulation (EU) 2019/6 will introduce a series of measures to improve control of antimicrobial use in veterinary practice, including generating a list of antimicrobial substances whose use would be restricted to people. However, the stakeholders were wary of such a list, since by design this will impact the available treatment options for animals. This makes clear the importance of measures for maintaining availability of existing antimicrobials, which may include finding novel approaches to model or extrapolate data so that old but important antimicrobials can meet updated requirements and be kept on the market. In addition, regulatory support and possibly other incentives should be given to promote the development of novel antimicrobials, or alternatives that could reduce the use of antimicrobials in animals such as immunostimulants (10). EMA will provide input into these matters and continue ongoing work including the implementation of the Committee for Medicinal Products for Veterinary Use's strategy on antimicrobials and measures to limit the effects of AMR.

#### Promote and Support Development of Veterinary Vaccines

The comments received reiterated the importance of vaccination, which is a highly effective tool for promoting animal health and welfare, safe food production and public health. They also highlighted the role that vaccines play in the reduction of use of antimicrobials, thereby contributing to combating antimicrobial resistance. In this capacity, veterinary vaccines, as well as biologicals that use innovative biotechnology, form an increasing number of authorization applications submitted to the agency. They help to overcome shortages in the pipeline of novel pharmaceutically active molecules and public concern about the safety of residues in foodstuffs of animal origin, as well as being a potential route to reducing the use of antimicrobials (10).

### Strengths and Limitations

Multiple researchers were involved in the survey design and five-stage iterative process of the framework analysis. We considered that this helped provide a comparatively thorough, dependable^1^, and objective survey and analysis, and we defend our results against researchers' bias, a limitation often cited in qualitative research. In addition, several researchers independently coded the qualitative data, and the summaries were drafted by consensus. We believe that this helped ensure the credibility^2^ of the results.

By providing all analyses and responses either in an appendix or on EMA's website (13), we ensure that our findings can be assessed for confirmability^3^ by others. Another strength to note is that, with the exception of human pharmacovigilance and environmental risk assessment, the topics covered are comprehensive of European regulatory science (see Supplementary Materials). Furthermore, the structured but free-text approach to eliciting opinions gave little restriction to comments, albeit framed to the starting proposals in the strategy. Nevertheless, and despite four workshops, an interactive written forum for discussion would have allowed more iterative feedback and clarifications.

The primary weakness of the paper relates to the external validity, or transferability, of the qualitative findings and quantitative results. We did not assess how well the characteristics of our respondents match the characteristics of EMA's stakeholders. This lack of comparison precludes any conclusion regarding the transferability or external validity of our findings to stakeholders that did not participate in the survey. By providing a basic characterization of the study participants (see “Overall number of responses to human and veterinary questions”), we encourage further discussions with stakeholders and readers of this paper on transferability to evaluate for which target groups this paper provides valuable information. Another limitation is that the qualitative summaries lacked detail for recommendations where few survey responses were obtained: the summaries provided in the veterinary area and, generally, the recommendations receiving a lower ranking. The individual responses are available on EMA's website for readers to dig deeper into these areas (13). The survey was also lengthy, and the question order was not randomized, so scoring fatigue may have limited the number of responses in the later Core Recommendations.

It is important to note that responses from the survey were weighted neutrally, meaning every response was counted as one participant, regardless of the size of the stakeholder or whether there were combined responses of several individuals, e.g., whether the number of members of an association is greater than a single individual. This is due to the blind nature of the analysis and differs from EMA's usual process of unblinded analysis, where weighting is implicitly applied to stakeholder size as well as quality of input. To adjust for potential sample size bias this may create, the unblinded responses, which contain the stakeholder size and whether the response was a combined one, were fed into the finalization of the RSS. In addition, larger stakeholders were invited to the workshops to give additional feedback.

### Implementation

The stakeholder views in this paper, along with those expressed at the workshops, have been systematically incorporated into the final RSS (10). Their implementation will stretch over 5 years, through four main streams. First, they will feed into the EMRN Strategy to 2025, which is a high-level strategy, spanning a range of topics but focusing on key areas challenging the network as a whole, including pharmacovigilance and the availability of medicines. The views of the stakeholders and the analysis within this RSS document will also feed into the EMRN strategy. The second stream is via the work programs of EMA's scientific committees, working parties, and other groups (14). Third, the EMA staff will be responsible for implementing many of these actions. Finally, the agency's proposed mechanism to fund regulatory science research will engage with national funding agencies and the European Commission to propose and issue calls across the areas identified in this strategy (15).

## Conclusion

The stakeholder views outlined in this paper, and those received during the iterative, multi-stakeholder consultation process as a whole, were instrumental in building the RSS. We hope that the views received from stakeholders and analyzed in this paper will not only guide EMA and EMRN priorities, but also inform stakeholders' own positions on the important topics identified as priorities for future. We also believe the methods outlined in this paper can serve as a resource for other decision-making bodies that wish to obtain stakeholder views for informing their decisions. In short, we hope these views and analyses can act as a regulatory science resource for public good.

## Data Availability Statement

The datasets presented in this study can be found in online repositories. The names of the repository/repositories and accession number(s) can be found below: https://www.ema.europa.eu/en/about-us/how-we-work/regulatory-science-strategy#:~:text=The%20European%20Medicines%20Agency's%20(EMA,both%20human%20and%20veterinary%20medicines.

## Author Contributions

PH, RJ, RG-Q, AL, and AH led the design, data acquisition, and analysis. All authors drafted the work, read and gave final approval of the version to be published, and provided substantial contributions to the interpretation of data for the work.

## Conflict of Interest

The authors declare that the research was conducted in the absence of any commercial or financial relationships that could be construed as a potential conflict of interest.

## Footnotes

^1^Dependability in qualitative research evaluates whether the process of research is logical, traceable, and clearly documented, particularly as to the methods chosen and the decisions made by the researchers.

^2^Credibility in qualitative research refers to whether the data fit the views of the participants studied (reference in attachment).

^3^Confirmability in qualitative research evaluates the extent to which findings are qualitatively confirmable through the analysis grounded in the data and through examination of the audit trail (reference in attachment).
