# Supplementary material for: Regulatory Science to 2025: An Analysis of Stakeholder Responses to the European Medicines Agency's Strategy
Source: Front Med (Lausanne). 2020 Sep 23;7:508. doi: 10.3389/fmed.2020.00508 (PMC7540226; doi:10.3389/fmed.2020.00508)
Supplement: Supplementary file 1 [file Data_Sheet_1.docx]

Analysis and summaries of public consultation results

EMA Regulatory Science to 2025

Table of contents

[1. Responses to the Human section of the RSS 3](#_Toc41302839)

[1.1. Qualitative analysis 3](#_Toc41302840)

[2. Responses to the Veterinary section of the RSS 50](#_Toc41302841)

[2.1. Qualitative analysis 50](#_Toc41302842)

[3. Survey questions 54](#_Toc41302843)

[4. List of acronyms 71](#_Toc41302844)

1. Responses to the Human section of the RSS
   1. Qualitative analysis
      1. Summary of responses to question 3 and 6

Question 3: “What are your overall views on the RSS?”

Question 6: “Are there any significant elements missing in this strategy? Please elaborate which ones”

Individual members of the public

Comments (N=8) from stakeholders identifying as individual members of the public were heterogeneous in nature. Whereas some responses recognised that the RSS addresses relevant issues, individual comments highlighted the need: i) for a stronger participatory patient engagement in decision-making, ii) to better accommodate SMEs and start-ups, iii) for early engagement with academic institutions, iv) to increase focus on improving public health, v) for better addressing the 3Rs and how to reduce animal testing in preclinical setting, vi) to describe plans supporting vaccine development in view of high unmet need due to lack of alternatives.

Patients and consumer organisations, patient advocacy groups and non-governmental organisations

The responses received (N=38) generally portrayed the strategy as relevant and comprehensive. Many of them, however, criticised particular aspects of the recommendations and highlighted the need for various improvements. Some patient organisations echoed each other’s views and some provided very similar comments.

Some responses highlighted that the strategy should be based on a clear analysis of what works well, and then take a more proactive approach to changing what does not, so that lessons learnt are implemented in the strategy. Responses expressed appreciation of the proposed innovative aspects of the regulatory science but were concerned that this focus should not be to the detriment of regulatory process improvements (e.g. pharmacovigilance). Existing patient and public engagement was applauded, but there was widespread agreement that EMA should be more explicit and ambitious about the value and methods for systematic and meaningful patient engagement in each of the strategic goals. More specifically, replies suggested that EMA encourage the development and use of robust methodologies to collect patient data, including patient registries; ii) enhance use of patient relevant and reported outcomes including, but not limited to, quality of life data, for informing benefit-risk (B/R) assessment; and iii) make patient involvement a key element in drug development and (early) evaluation activities by setting standards and expected outcomes for all actors and activities.

Many highlighted areas for improved communication, transparency and debate about regulatory decision-making, to ensure public trust and understanding in a time of increasing scientific and regulatory complexity. Regarding communication, some also specified that the RSS lacked detail on how EMA would ensure effective, lay language communication of side effects or adverse drug events to the public (e.g. in the package leaflet). Some replies requested EMA to critically reflect on regulatory schemes, with specific focus on early access schemes with increased uncertainty (e.g. PRIME, conditional marketing authorisation); some suggested to do this via a critical reflection on the shortcomings, uncertainties and added value related to approved drugs. Several responses also stated that a critical review on the implementation of the orphan drug legislation was missing and argued that this would ensure that the legislative incentives are not abused to the detriment of patients. A number of responses expressed the view of the proposed increased interrelations between advisory and evaluation activities, there is a need to better describe how EMA would address the handling of conflicts of interest of experts within these activities. This was argued to be particularly relevant in the context of rare diseases where there is a small pool of experts. Many called for the set-up of an independent ethics committee to ensure there is no conflict of interest, in addition to a more stringent policy.

Replies also called for the EMA to make publicly available more documents describing its activities and (reasoning behind) decisions, including clinical trial and observational study data (protocol, results including individual anonymised patient data), scientific advice, committee meeting reports and management decisions. They felt the RSS should reflect how both the Agency and national competent authorities (NCAs) could ensure better reporting of clinical trial results. It was mentioned this would foster open science, enable public understanding, scrutiny and in the case of clinical trial results, independent re-analyses.

Several patient and consumer groups argued that EMA should be more stringent in its decisions and evidence requirements; the latter regarding both pre- and post-approval studies. Similarly, responses stated that EMA should help ensure clinical relevance of approved medicines, e.g. by demanding clinical trials with clinically relevant endpoints and comparators, such as current standard treatments, by including the real target population (e.g. elderly), or by critically reflecting on the added therapeutic value of medicines. Regarding clinical trials, some responses argued that whenever possible: i) EMA should require two RCTs conducted by independent organisations, ii) these should test superiority rather than non-inferiority and iii) EMA should require the investigation of shorter treatment duration during clinical trials, specifically in the case of adjuvant trials (as there was a concern that treatment duration is currently being selected based on return on investment). It was also suggested that statistical analysis of raw data be performed by EMA (“in house”) and reported transparently to allow independent re-analysis.

The majority of responses generally welcomed EMA’s intent to take a broader role both pre- and post-approval. Some also discussed the strategy as going beyond EMA’s remit in reaching downstream to HTA and Payer decision-making, with mainly positive views on this.

However, some responses expressed concern that by implementing the RSS, EMA would become a “co-developer” rather than a “gatekeeper” of medicines. Many demands were made to enforce EMA`s role as “gatekeeper”. Such responses generally felt the strategy overly favoured innovation, industry interactions and faster marketing authorisation rather than focusing on regulatory responsibilities and public health.

In this sense, there was also widespread concern at the lack of an overt focus in the strategy on pharmacovigilance and how to enhance it, given that it is a core mission of EMA. This was particularly important given the perception of a shift towards post-approval evidence generation. Several underlined the need to make the pharmacovigilance system more proactive in responding to safety concerns. It was also argued that the RSS needs to further clarify and uphold evidence requirements for post-marketing studies: more specifically, post-marketing studies and endpoints should be designed to address uncertainties at the time of marketing authorisation, and ideally within specified time limits. Stakeholders also highlighted that the trend for data collection post-marketing may cause a shift in the burden of evidence generation away from the developer and onto the healthcare system (e.g. physicians). Some responses underlined the need for increased patient involvement to improve existing pharmacovigilance and adverse event reporting.

There was also a call to make better use of pharmacovigilance and real world data (e.g. in disease registries) to: i) supplement the data for small patient population clinical trials (e.g. rare diseases, elderly, pregnant/lactating women) and ii) to actively challenge the B/R of approved medicines, leading to withdrawals of drugs that appear to be ineffective or unsafe when marketed.

It was recommended to elaborate more on data protection in the strategy and to ensure EMA aligns with EU law and policy on this and on cyber security. The importance of patients’ access to medicines was echoed, and in particular, therefore, aligning evidence generation requirements to meet demands of HTAs, payers and society.

Replies described plans for prioritisation and implementation (“deliverables”) of the strategy as missing. More specifically, in view of the ambitious nature of the RSS, the lack of mention of where the required expertise would come from and the budgetary impact of suggested measures was highlighted. Some responses highlighted that although activities in the document were segregated into silos, in practice many are interdependent and would need to be drawn together.

Healthcare professionals and healthcare professional organisations

Responses (N=19) from EU healthcare professionals and organisations varied in nature and highlighted diverse topics. Although this makes it difficult to draw out common themes, they generally praised the ambition of the strategy and found it comprehensive. Several responses stated they would like to see further collaboration with stakeholders (e.g. EU institutions) as work in some areas of the recommendations (e.g. identifying and prioritising unmet needs) is also carried out by these stakeholders and collaboration would increase feasibility and reduce waste of resources. Responses stated that the strategy should elaborate more on the topic of medicines’ supply and preventing shortages. Again, it was pointed out that the strategy is missing a strategic goal on pharmacovigilance. Regarding RWD, a need to establish and validate methods and requirements was mentioned. There was also apparent need to do more to integrate digital advances.

Academia

Academics (N=25) generally considered the strategy relevant and comprehensive. However, the challenge of implementing the RSS was also repeatedly raised, with consideration required for the changing landscapes of resources, European politics and scientific expertise (e.g. Brexit). It was suggested that including a more concrete plan of action to achieve the RSS goals would help to generate trust, given the ambitious nature of the strategy. Some replies highlighted the difficulty of singling out the most important recommendations as they considered that many of them are connected and could be grouped to maximise impact.

Several emphasised the need for facilitating greater collaboration and communication with and between stakeholders, including patients, academics and industry; this was seen as key in sharing knowledge, resources, and developing and harmonising standards, as well as to ensure the feasibility of the RSS. Collaboration was also seen as being necessary in the changing field of ATMPs.

The need to show leadership in enhancing patient-engagement was stressed. This included elaborating on patient engagement across the drug lifecycle, including EMAs processes, and developing metrics for this. The strategy should describe concretely how the patient community can better understand and contribute to the regulatory process. The value of developing a set of Patient Important Outcomes to be measured in all clinical trials in a specific disease and for use by downstream decision-makers was also raised and the example of core outcome set (COS) was provided.

Responses highlighted the need to bolster efforts on replacement, reduction and refinement (3Rs) of animal testing: incentivising and harmonising the qualification of novel methods across the human and veterinary areas, then clearly communicating their regulatory acceptability.

Responses also recognised the need to enhance regulatory preparedness in characterising and assessing nanomedicines as well as medical devices, combinations of medical devices and drugs, in vitro products and borderline products.

Two respondents stated that funding bodies should be made aware of the RSS goals, particularly the regulatory science research questions, to align funding priorities.

Other Scientific Organisations

Overall the responses (N=33) from 'scientific organisations' were positive about the strategy and its comprehensive content. However, they stressed that EMA must enhance stakeholder collaboration across the board: with HTAs and Payers, to improve evidence generation and to close the gap between approval and access; with public-private partnerships (PPPs) and SMEs, to leverage their efforts in regulatory science projects; with manufacturers and suppliers (medical product/technology), to promote interactions regarding new innovative technologies in manufacturing of medicinal products and medical devices; and with patients, taking concrete steps towards systematic patient involvement in drug development and evaluation.

They also pointed out the need to encourage data sharing across the EU network and that the EMA should focus on data sharing and data pooling. A few responses explicitly mentioned the need for EMA to align with other regulatory agencies (FDA, NCAs, PMDA) in many areas such as requirements for clinical trial design and analysis to improve the efficiency of drug development. This was found particularly important for rare diseases, vulnerable populations (e.g. neonates) and the development of ATMPs. It was noted that the strategy does not address current discussions around the lack of clinical benefit of new medicines.

More information was requested about resources and the practicalities (e.g. timelines, operational approach, opportunities for stakeholder involvement) of implementation, and the need to outline more detailed and concrete actions to achieve the goals.

EU Regulatory Partners / EU Institutions

Overall, responses (N=20) were generally positive, seeing the RSS as comprehensive and as a basis to subsequently develop the EU Network strategy. However, many saw a need to clarify how the RSS will be turned into actions and changes in day-to-day work of EMA and NCAs (IT changes, guidance development, training of staff and additional budgeting), and how other stakeholders and specifically NCAs will be involved in the strategic direction-making and implementation of the RSS (particularly as many of the elements it covers are regulated/implemented by NCAs e.g. clinical trial approval). NCAs should be adequately informed and resourced to enable such involvement, collaboration and knowledge sharing for proper implementation of the RSS. Some Member States with well-established RS programmes suggested the need to leverage existing initiatives and collaborate with them. These elaborated on the need for a common network strategy and detailed description of a mechanism that enables continued interaction between EMA and NCAs. Overall, responses found a lack of consideration of the sustainability of the current regulatory system and the need to improve it by addressing issues such as the process of delivering and updating guidance, evaluating the functioning of working parties and committees, and increasing predictability/precision in timing of submissions.

Like other stakeholder groups, several responses stated that there should be more focus on pharmacovigilance, in particular of medicines that represent innovative technologies or authorised on the basis of innovative pre-clinical models, clinical trial designs, or approval schemes.

Several replies acknowledged the importance of big data, RWD, data management and the need to foster data sharing and interoperability. A few considered that developing regulatory standards for assessment of data provided by digital technologies should be prioritised. The role of academia and non-industry stakeholders engaged in big data and RWD was specifically raised with regards to training and integrating them into the regulatory environment

Some underlined that for implementation of the RSS, the digital transformation of regulatory processes need to be prioritised and adequately resourced, including training. The RSS should also align with the telematics strategy and involvement with a greater focus on data quality and standardisation.

It was also emphasised that the RSS could benefit from explicitly describing how patient experience would be translated into patient science (e.g., standardisation of outcomes, validation of data and sources). Some also considered the proposals lacked ambition regarding open science and making data available e.g. publishing environmental risk assessment data, or anonymised safety data from Eudravigilance. On the topic of the environment, there were comments that the strategy lacks any focus on environmental sustainability (specifically the reduction of pharmaceutical emissions in the environment) or linkage to the “Strategic Approach to Pharmaceuticals in the Environment” published by EU commission.

A few responses focused on the need to ensure that patients have timely access to medicines that meet their medical need on the basis of the severity of the disease and lack of alternative therapies. They suggested that EMA increases its role in reducing time between approval and access specifically with regards to innovative products which require strong collaborations amongst NCAs, other healthcare actors and other EU national healthcare bodies.

Health technology assessment bodies

Overall, responses from HTA bodies (N=9) were very positive about the proposed collaboration and exchange of information with EU and national regulators. The majority called for further alignment on scientific advice regarding requirements for pre-approval evidence generation and patient relevant endpoints. However, a few replies were against EMA expanding its role beyond its remit; such as i) biosimilars uptake, ii) drug shortages, iii) assessment on added value and iii) data generation for downstream decisions. These suggested EMA's role to be limited to information sharing and facilitating cooperation between stakeholders (HTA/payers).

There were several calls to: i) refocus the strategy and EMAs role on public health, away from innovation; several emphasized that EMAs role should be to defend public interest and promote public health and not as a co-developer of medicines and ii) prioritise a critical reflection on how current regulatory approaches (e.g. risk assessment/pharmacovigilance, conditional MA, withdrawing MA, orphan drug designation etc.) can be adapted.

There were also calls to reinforce the quality of evidence and the stringency of requirements for clinical data. This was considered especially important for pre-approval studies since this evidence is unlikely to be generated post-approval and is essential for informing both payer/reimbursement decisions and clinical evidence-based medicine. For pre-approval studies, it was asked that EMA should (unless otherwise fully justified): i) require comparative RCTs vs standard of care, ii) ensure the real target population is fully represented in these RCTs, iii) require that one of the 2 RCTs be performed by an independent party, iv) require superiority trials rather than inferiority trials, v) consider the duration of treatment in the assessment process, vi) ensure the primary endpoint is a clinically or patient-relevant outcome. Several also highlighted the importance of improving requirements for evidence generated post-approval (RWD, clinical studies), e.g. via the development of international good practice guidelines for collection of RWD.

Some advocated limiting use of accelerated approvals and (conditional marketing authorisations) CMAs, as the responses considered that they increase uncertainty for downstream decision-makers (HTA bodies, payers, healthcare providers, patients) and pose safety risks for patients.

Payers

The five payer responses were mainly negative with regards to the perceived focus of the document on technological innovation above public health. However, they welcomed the collaborative elements of the strategy related to HTA/payer bodies.

They mentioned the need for assessments to take into account the clinical relevance and the impact of approving medicines on public health, for example diverting resources from other therapies, or time spent in hospital. They reiterated HTA bodies’ comments regarding the need to focus on B/R and be cautious and stringent as a regulator, both in pre- and in post-licensing evidence generation. Regarding RWD, concern was raised that RWD is prone to bias and not mature for regulatory uptake.

Several expressed concerns over implicit focus on fast access to innovative products (EMA potentially regarded as a medicines’ co-developer), as opposed to demonstrating clinical benefit and positive B/R balance: pre- and post-approval. Mirroring HTA body responses, payers argued against the use of CMAs and accelerated approvals and they questioned the timeliness of fulfilment of the conditions imposed on marketing authorisation holders under these regulatory schemes.

Further echoing some HTA responses, they wanted the RSS to include a critical reflection of: i) EMA’s current methods and potential adaptations (e.g. risk assessment/pharmacovigilance, withdrawing marketing authorisations MA, orphan drug designation), ii) EMAs role, which should not include the evaluation of the added value of therapies, iii) the transparency and availability of data submitted by the marketing authorisation holder to the agency, iv) EMA’s possible role in guaranteeing market launch in all European markets, for example via a ‘medicines tracker’ as suggested by HTA bodies.

Pharmaceutical industry - Individual companies

Pharmaceutical company responses (N=39) were generally positive about the document, welcoming the focus on innovation, its consultative nature and the EMA undertaking forward looking activities. They echoed each other’s feedback, adding company-specific views on certain areas. They advocated greater collaboration between stakeholders and the EMA and stressed the importance of efforts which reduce the complexity of the European system, seek global alignment, and advance patient-centred access.

The ambitious nature of the strategy and consequent need to consider the strategic prioritisation, implementation and need for additional resources, training, expertise and efficient IT structure and IT systems were also repeatedly mentioned. To be able to implement the RSS, secure resources and develop expertise, EMA should collaborate with EU regulatory partners (NCAs, EC), as well as with FDA. Implementation was also viewed as requiring concrete actions, frequent status updates and an interim evaluation.

Industry responses stated that to enable and leverage research and innovation, industry-based researchers should be acknowledged alongside researchers in academia. Furthermore, information technology companies were cited as necessary partners as they undertake research and have expertise in the fields of eHealth, big data, artificial intelligence, wearable devices etc.

Whereas several responses suggested specific areas to be prioritised, others mentioned prioritisation should take into account the interdependent nature of the Core Recommendations, as some Core Recommendations are enablers of others so delivering on the priorities may imply progressing others too. Individual companies also stressed the need to prioritise ongoing important EMA projects close to completion with longer-term ambitions, taking into consideration the changing EU landscape (e.g. Brexit, new Commission) and budgetary constraints. Regarding BREXIT, a few responses stated that the RSS should address the implications of Brexit; its potential impact and how to strategically tackle future challenges.

An objective which some responses suggested was to include the optimisation of existing IT tools and systems in order to reduce the collective administrative burden and enhance their value. Additionally, current regulatory procedures were criticised for their administrative burden and lack of speed and flexibility.

Responses underlined that the RSS lacks acknowledgement of the importance of greater alignment in regulatory science and regulatory frameworks with other international organisations (in particular FDA, ICH), including alignment on key priorities and timelines. Some suggested to develop detailed goals and recommendations to achieve global alignment on key priorities such as RWD. Regarding RWD, since this is an ambitious topic, they suggested to collaborate with FDA from the beginning to ensure global harmonisation. It was also suggested that EMA takes a lead role in developing a multi-stakeholder framework, including RWD methodology requirements, through pilots and workshops. For clinical trial innovation, pilots were also recommended as well as improving ICT infrastructure. Another opportunity was seen in harmonising clinical development plans and SA, where they wanted alignment amongst multiple stakeholders e.g. patients, NCAs, HTAs, health systems and the FDA. Throughout industry’s feedback, FDA was repeatedly cited as regulatory comparator of best practice.

A few replies highlighted that the RSS lacked actions regarding post-marketing surveillance; One example mentioned was to expand benefit-risk assessment to use new post-authorisation evidentiary sources in order to identify better safety signals for action.

The responses were positive about improving the flexibility and harmonisation of scientific advice, particularly involving stakeholders. They also expressed the importance of the proposals for modelling and simulation, clinical trial innovation, RWD, cooperation and alignment with decision makers, manufacturing, ATMPs and regulatory science expertise and research.

There were mixed opinions regarding efforts to coordinate with HTA decision-making, with some seeing it as important to streamline decision-making, however others considered it was too political and complicated.

Responses stressed the importance of making best use of technology and data to support new healthcare paradigms. One example frequently mentioned was the development of a shared digital space where real-time data is available (i.e. a cloud-based environment). It was argued this would allow all interested stakeholders to access the available data throughout the life cycle (including review and decision-making) and would create a more efficient, reliable and transparent assessment process.

Pharmaceutical industry – Trade associations

The replies from pharmaceutical trade associations (N=19) had many shared priorities, particularly in the areas of clinical trial innovation, multi-stakeholder scientific advice harmonised across the EU, real world data and manufacturing. Trade associations noted that innovation in clinical trial design and analysis as well as in use of RWD requires European and international harmonisation. Researching the value and reliability of RWD through pilots and leveraging knowledge gained by other stakeholders and international partners was considered necessary to agree on global standards and requirements. Responses suggested that scientific advice could benefit from increasing flexibility and aligning with NCAs and other stakeholders (e.g. patients, HTA bodies). Collaboration with, and leveraging of, efforts from other stakeholders/partners (academia, industry, NCAs, HMA, HTA, patients, FDA) was seen necessary for the implementation of the RSS (including harmonised scientific advice). Specifically, the importance of closely involving NCAs regarding recommendations on clinical trials and drug availability was noted as certain areas of the RSS fall within their competence. International harmonisation was noted as a missing element in certain areas such as RWD and clinical trial innovation. Responses expressed support for increasing regulatory science knowledge exchange between stakeholders.

Replies supported developments in precision medicines, biomarkers and ‘omics’ and the creation of an integrated evaluation pathway for the assessment of medical devices, in vitro diagnostics and borderline products. The importance of increasing patient engagement and patient-centred access was stressed.

There were several suggestions to optimise existing regulatory frameworks. Examples mentioned were the generic and biosimilar framework (see below) and harmonisation of bioequivalence requirements to reduce number of clinical trials and to clarify the level of evidence required for meaningful scientific advice.

Improving ICT infrastructure and implementing novel digital technologies were seen as fundamental to deliver the recommendations. Responses stressed that digital EU-harmonised IT systems would improve the efficiency of the regulatory system.

Responses highlighted that in view of the ambitious nature of the RSS, there is a need for resourced and holistic implementation and prioritisation, more so because recommendations were seen to be interrelated and interdependent. Several also highlighted the need to outline concrete steps, resources (financial and human) and deliverables and, following this consultation, industry stressed their expectations of the EMA’s 5-year implementation work plan, which will detail the actions the Agency is focusing on to achieve its regulatory science strategy. Priorities should be communicated to enable other stakeholders to also focus their resources on them. Some responses encouraged EMA to balance its delivery of near-term process improvements with long-term objectives. Some Core Recommendations are enablers rather than objectives per se. Therefore, implementation will need to be mindful of this and the order in which they are progressed needs to be considered.

Echoing comments from all other stakeholder groups, a few replies mentioned that concrete actions regarding pharmacovigilance were lacking; as by individual companies, the example of expanding benefit-risk methodologies for post-authorisation assessment was highlighted.

- - 1. Summary of responses to questions 5 and 7

Question 5: “Please identify the top three Core Recommendations (in order of importance) that you believe will deliver the most significant change in the regulatory system over the next five years and why.”

Question 7: “The following is to allow more detailed feedback on prioritisation, which will also help shape the future application of resources. Your further input is therefore highly appreciated. Please choose for each row the option which most closely reflects your opinion. For areas outside your interest or experience, please leave blank. Should you wish to comment on any of the Core Recommendations (and their underlying actions) there is an option to do so.”

Goal 1: Catalysing the integration of science and technology in medicines development

1. **Support developments in precision medicine, biomarkers and ‘omics**

*Individual member of the public*

Individual members of the public (N=2) did not mention whether they were supportive or not of the actions included in this recommendation.

*Patient or Consumer Organisation*

Two HCP organisations presented different views and suggested different actions.

*Healthcare professional organisation*

Of the four HCP organisations which responded, some were in favour of the action on the impact of emerging ‘omics’ methodologies and added that the imaging part of ‘omics (radiomics) and biomarkers should be included in the RSS as this will play an important role in drug efficacy assessment.

*European research infrastructure*

Both responses were supportive of this recommendation and EMA’s role as a regulatory body in the development of precision medicines, biomarkers and ‘omics.

*Other scientific organisation*

The majority of other scientific organisations who responded (N=5) were very supportive of the Core Recommendation on supporting developments in precision medicine, biomarkers and ‘omics, as they considered pursuing this recommendation would reinforce and support other strategic goals and Core Recommendations.

The Core Recommendation 1.2 (support translation of ATMP into patient treatments) was found to be linked to this recommendation, as biomarkers, ‘omics, and novel therapies to enhance precision medicine are fundamental to understanding how ATMPs will affect patients. Some responses stated that EMA must keep pace with scientific advances and address the integration of science and technology by ensuring that the current and future regulatory pathways are flexible enough to accommodate these evolving biomarkers and other ‘omics-based tools and technologies. Many noted that significant public health benefit could be gained by integrating knowledge and tools based on data and evidence in precision medicine, biomarkers and omics and applying it to human therapeutics.

Some highlighted that regulatory guidance should be expanded to not only cover single biomarkers but also new biomarkers under development, biomarker panels and individual biomarkers. Furthermore, regulatory advice at present was stated to be only available through EMA qualification procedures and Innovation Task Force. Some thought it would be more beneficial to have more frequent interactions within these fora. In addition, some found that the current EMA qualification procedure should be enhanced by allowing the procedure to be accelerated and have greater flexibility, or a different pathway should be developed to discuss biomarker development outside of the qualification procedure to facilitate rapid progress.

Early and proactive engagement with stakeholders at all levels (including novel biomarker developers as well as industry and academia) was stated to be key in facilitating the development of novel human therapeutics with improved benefit-risk assessment and to overcome challenges.

*Health technology assessment body*

The few comments received (N=3) focused on the need to develop new methodologies that allow for the use of scarce data for approvals of medicines for small patient groups, and the importance of biomarker validation

*Payer*

The two almost identical responses from payers stated that EMA should provide a more detailed description of the procedures for biomarker validation to be followed by applicants, and that it must be the applicants’ responsibility to carry out this validation before marketing.

*Pharmaceutical industry*

Individual company

Of the individual companies who responded (N=7), many were extremely supportive of this recommendation. Several welcomed the requirement for EMA to enhance engagement with novel biomarker developers and academics and early engagement of regulators with developers was considered to facilitate regulatory qualification. However, a few believed that the requirements for qualification need to be more practical and flexible as the existing approach presents high barriers which impedes the ability to rapidly develop innovative treatments. Some recommended that the qualification pathways of biomarkers should begin early via scientific advice procedures, including broad scientific advice. Some suggested that EMA could also offer an alternative pathway, outside the qualification process, to speed up progress. Many believe that enhancing the validation process would encourage a greater uptake and use.

It was repeatedly suggested that EMA needs to collaborate with HTAs to ensure greater alignment on data requirements, since HTAs often challenge use of biomarker endpoints as a primary source of evidence for decision making. This collaboration could be sought through a dedicated expert group and could help EMA and downstream regulators to better align their views. The current setup is not satisfactory for these innovative personalised medicines.

Further collaboration with academics, other regulators and industry is needed to address new challenges arising from the developments of novel human therapeutics and to improve benefit-risk ratios as large amounts of data become available. A few responses suggested developing a diagnostic testing infrastructure in EU.

EMA could focus resources to stimulate the development of new validated clinical endpoints as these will be essential for the progression and approval of novel therapies for serious and life-threatening diseases, ultimately improving patient access. Some contributors suggested that industry should be involved in the impact assessment of treatments on clinical outcomes measured by biomarkers.

A few responses requested EMA should focus on the following recommendations in order to realise the potential of precision/ personalised medicines to evolve into personalised healthcare:

- Create an integrated evaluation pathway for the assessment of medical devices, in vitro diagnostics and borderline products
- Diversify and integrate the provision of regulatory advice along the development continuum
- Foster innovation in clinical trials
- Develop the regulatory framework for emerging clinical data generation
- Optimise capabilities in modelling, simulation and extrapolation

Trade associations

Of the trade associations which responded (N=4), most welcomed the Core Recommendation and its underlying actions. In order to further encourage greater uptake and use and fit the new challenges arising from these new therapies, a few recommended that EMA make adaptions to the regulatory pathways by developing biomarker validation processes.

1. **Support translation of advanced therapy medicinal products (ATMPs) into patient treatments**

*Individual members of the public*

Two responses were received from Individual members of the public. These held opposing views on the level of support that should be offered to ATMP development.

*Patient or Consumer Organisation*

The three responses received proposed few additional actions. However, it was suggested that EMA should make it a priority to ensure collaboration with patients, healthcare professionals, academia and international partners in order to support translation of ATMPs into patient treatments. In addition to the development of payment models for ATMPs, EMA should highlight the issue of high prices for ATMPs which hinders accessibility for patients.

*Healthcare professionals*

Both healthcare professionals who responded considered the ATMP field an essential part of innovation and one that offers ground-breaking new treatment opportunities.

*Other scientific organisations*

The other scientific organisations who responded (N=4) largely agreed that ATMPs have the potential to change the therapeutic landscape to the benefit of patients and supported the underlying actions proposed. Some believed that EMA should engage with other European authorities (Heads of Medicines Agencies and EU-Innovation Network) and international regulatory agencies to foster global convergence on the regulatory requirements for ATMPs, as several aspects of the requirements remain the responsibility of EU national authorities. A number stated the need for appropriate regulatory tools and underlined that new or adapted regulatory paradigms should be able to deal with ATMPs.

*Health technology assessment bodies*

Responses (N=4) broadly supported the recommendation and there was a general support of most actions.

*Payers*

The two very similar responses from payers indicated that there was a need for a clear and universal definition of unmet medical need that is based on the public health perspective. It was suggested that EMA puts in place procedures to allow stakeholder involvement throughout the ATMP lifecycle but ensuring that their participation remains impartial and transparent. Furthermore, as with the HTA bodies they considered that the Agency should create relevant processes to re-evaluate or withdraw products which do not meet requirements post-approval.

*Pharmaceutical industry*

Individual companies

The recommendation and underlying actions were seen as valuable and important in the responses (N=7). A few individual companies noted that the action to identify therapies that address unmet medical needs will require collaboration with multiple stakeholders including industry in order to provide a wide range of perspectives, specifically with regards to evidence generation. It was also suggested that HTA bodies, payers and patients should be involved during the development of new treatments. It was anticipated that the EMA will play a key role in coordinating this recommendation and that EMA will further build on their previous work with other stakeholders in order to meet the needs of patients.

Furthermore, some noted the need for global convergence in the development of the ATMP regulatory framework as there are currently inconsistencies between national and European standards. Some felt that regulatory requirements should be flexible in order to be adaptable to more advanced technology and allow improvements in the manufacturing process of ATMPs.

Trade associations

Of the trade association’s responses (N=6), most supported the recommendation and some specifically focused on its underlying actions.

Several suggested that EMA should consider creating a regulatory framework supporting a multisource environment for ATMPs that will lead to a competitive market and thus more affordable therapies in the future in the EU. These regulatory pathways would be designed to stimulate evolution of the innovation lifecycle and increase patient access to such therapies. Furthermore, for innovation to be converted into patient treatments, a more unified system was seen as vital with cross-fertilisation between advancing science/clinical developments and the regulatory environment.

A few responses highlighted the need for HTA involvement with regards to data requirements to avoid the inconsistent understanding of the different requirements for market access for ATMPs. However, some believed multi-stakeholder discussions would also further improve how these products were assessed for efficacy/effectiveness compared with other treatments.

Responses indicated a need to ensure better cooperation between the European Commission and national agencies/Genetically Modified Organisms authorities in the Member States and to ensure better alignment between EMA and national regulators. Continuous and effective dialogue with stakeholders would also allow for a more efficient development of products. A few contributors suggested that a specific platform for knowledge sharing could be developed and would facilitate the development of specific expertise and increase capacity across the EU regulatory Network to assess ATMPs.

Some responses noted the interdependence with other recommendations in the strategy:

- Diversify and integrate the provision of regulatory advice along the development continuum;
- Contribute to HTA’s preparedness and downstream decision-making for innovative medicines.

Some responses also noted that ATMPs will eventually become off-patent. This would require the development of appropriate regulatory pathways for the future development and registration of off-patent ATMPs, since the regulatory paradigm is different from that of new chemical or biological entities.

1. **Promote and invest in the PRIME scheme**

*Patient or Consumer Organisation*

Amongst the three responses, it was explicitly stated that the PRIME scheme should apply only in fully justified circumstances and must ensure that patient safety is guaranteed. They considered previous experience to have shown that monitoring of post-licensing evidence generation is often problematic, resource-intensive and exposes patients to harm.

*Other scientific organisation*

With the rapid pace of scientific knowledge and process of innovation, responses (N=5) agreed with the action to leverage collaboration with stakeholders. However, contributors stated that an efficient and flexible continuum of dialogue with stakeholders (academic, sponsors and patients) could allow a more efficient and ethical way to learn and develop products, shorten timelines and ensure optimal trial design. Furthermore, global collaboration and better alignment in defining clear, assured requirements between regulators in different jurisdictions would benefit manufacturers and patients alike. In addition, resources from other regulatory agencies should be pooled together to enable rapid scientific advice and enhance global development of PRIME products, such as ATMPs.

PRIME could be further enhanced through incentives such as extension of market exclusivity for neonatal and rare paediatric diseases or support for sponsors to pursue high-risk clinical programs in rare diseases. Early interactions and more diverse communication with regulators would allow for the integration of more frequent regulatory advice to shorten the duration of the development process. To ensure that PRIME products translate into effective patient treatment in a timely manner, promotion and investment in the PRIME scheme should continue and be enhanced.

*EU Regulatory partner / EU Institution*

Contributors (N=2) agreed that the PRIME scheme allows important medicinal products to reach patients faster and supported the action to shorten the time between scientific advices, clinical trials and MAAs. However, they all also noted that shorter time frames for often complex products can pose a challenge to both industry and regulators. Responses suggested that the current regulatory system that supports the PRIME scheme needs to be further optimised in a sustainable manner with the ability to shorten the time frame while ensuring pre-licensing evidence generation plans of sufficient quality to ensure that approval can result in faster and better access for patients.

*Health technology assessment body*

No consistent views were noted among the responses (N=5) to this question. Among the individual responses, one noted, as in the previous section, that no specific actions were defined in this element of the strategy to encourage the crucial HTA involvement and ensure their evidence requirements were met during development.

*Payer*

Of the responses (N=4), some considered that the promotion of PRIME scheme was not necessary for EMA’s regulatory science strategy to 2025. They believed that shortening the time for marketing authorisation led to a greater reliance on post-marketing evidence generation, resulting in a risk shift to patients and healthcare systems. They suggested that PRIME should be limited to only selected cases of high unmet medical needs which are clearly defined in advance by stakeholders. It should also be examined whether PRIME is able to fulfil its goal of accelerating market access while improving available evidence.

Others believed that a uniform and clear definition of unmet medical needs, based on a public health perspective, was crucial and should be used as an entry criterion for the PRIME process. Like some HTA bodies, some felt that EMA needs to develop methods for an impartial and transparent participation of all stakeholders involved throughout the life cycle, including HTA and payers. There was also a need to reduce the risk of bias between the assessors and developers through their early interaction.

Several contributors noted the need for EMA to develop a process to re-evaluate and withdraw marketing authorisations when products do not meet patient needs in the long term.

*Pharmaceutical industry*

Individual company

Of the contributors (N= 8), many fully supported the recommendation and its associated actions, specifically shortening the time between scientific advice and MAA submission. A few mentioned their support for the PRIME scheme, its effectiveness and benefits. An evaluation and review of the current scheme and further enhancement of the system was welcomed. Some suggestions included: reducing administrative duplication; expanding the scheme to line extensions/ new indication based on the same criteria as for an initial first indication and with aligned opportunity for accelerated assessment; allowing more flexibility in the opportunities for scientific advice. Leveraging and enhancing collaboration with all stakeholder, including international partners, is key to further the development of PRIME.

Trade associations

Amongst the trade associations who responded (N=6), the proposal to promote and invest in PRIME is supported by nearly all as it has proved to be of real advantage for companies involved in early drug development, to improve developed plans and accelerate evaluation. However, many believe that there are opportunities to further optimise implementation of PRIME. A review of the performance of the PRIME scheme is recommended by many contributors as recent data demonstrates that EMA’s review timelines have increased and are becoming much longer compared to FDA.

Some suggested that to ensure that all applicants continue to see the benefit of using the PRIME scheme, an accelerated approach would need to be designed for PRIME products and would include:

- Shorter timeframes for eligibility and kick-off meeting
- Continuous dialogue with an EMA contact person
- More occasions to receive advice on product development

Many agreed that that involvement of HTA bodies and payers in PRIME is key to ensure that scientific advice takes into account the generation of data along the development lifecycle to satisfy the needs of downstream decision makers on reimbursement and patient access.

An important aspect of the recommendation agreed by a few responses was the need to improve communication and collaboration with stakeholders, such as patients, healthcare professionals, academia, and international partners. In particular, involvement of patients and healthcare professionals in multi-stakeholder early dialogue is indispensable.

1. **Facilitate the implementation of novel manufacturing technologies**

*Individual member of the public*

Two individual members of the public responded, suggesting the Agency focuses on specific areas.

*Healthcare professional and Healthcare professional organisations*

One healthcare professional and one HCP organisation responded. The two had divergent views on their support of the Core Recommendation.

*Academic researchers and other scientific organisations*

Other scientific organisations (N=3) and one academic researcher supported the Core Recommendation. They requested guidance and standards for novel manufacturing, in particular for decentralised ATMP production.

*EU Regulatory partner / EU Institution*

EU Regulatory partners (N=3) supported the initiative, stating the importance of novel manufacturing for improved quality, safety and efficacy.

*Payer*

Payers (N=3) supported the Core Recommendation. They agreed that that regulatory models need to be reviewed to adapt to novel manufacturing methods, medical devices and new materials. However, this must be done through putting public health first and never lowering regulatory standards.

*Pharmaceutical industry*

Individual companies and trade associations

Industry (N=10) and their trade associations (N=5) largely welcomed the initiative, although some questioned the relevance of the proposed underlying actions. Nevertheless, there was agreement that innovation in the development of pharmaceuticals may be hamstring by lack of innovation in manufacturing and CMC and that this problem could be remedied by EMA action. One suggestion was to encourage the application of modelling and simulation to the CMC context. The responses also asked to have access to early, flexible and continuous advice on manufacturing and CMC issues (e.g. EU PAT team). They requested that EMA sets up structured communication with health authorities to define regulatory and GMP gaps and the actions needed to address, whilst ensuring international harmonisation in novel manufacturing guidance. They also asked for a clear regulatory pathway for technology changes affecting a platform of products or sites, rather than just one dossier.

1. **Create an integrated evaluation pathway for the assessment of medical devices, in vitro diagnostics and borderline products**

*Individual member of the public and Patient or Consumer Organisation*

Members of the public (N=2) stressed that work was needed to reduce the fragmentation at the member state level, and that EU action can help. Patient and consumer organisations (N=2) urged a robust focus on benefit/risk assessment.

*Healthcare professional organisations*

Healthcare professional organisations (N=3) welcomed a clear regulatory pathway for borderline products and encouraged rigorous standards.

*Academic researchers and Other Scientific Organisation*

An academic researcher and another scientific organisation highlighted the uncertainty around medical devices and the regulatory borderline.

*EU Regulatory partner / EU Institution and Payer*

EU Regulatory partners (N=6) and the payers (N=2) who responded echoed the importance of an evaluation pathway for the assessment of MD, IVD and borderline products. They requested guidance and a structured cooperation between NCAs and notified bodies. They acknowledged that regulatory models need to adapt to innovation in this area. However, they cautioned that this adaptation should not reduce regulatory rigor and should be supported by relevant and constantly updated expertise.

*Pharmaceutical industry*

Individual companies and trade associations

The pharmaceutical industry (N=5) and its trade associations (N=11) stressed the importance and urgency of creating an integrated evaluation pathway for the assessment of medical devices, in-vitro diagnostics and borderline products. The new Medical Devices Regulation (MDR, Regulation (EU) 2017/745), enters into force on 26 May 2020, and lacks clarity on the regulatory processes and requirements for such products. There is a clear need for the roles and responsibilities of Notified Bodies and National Competent Authorities/EMA to be outlined. This need is particularly pressing given the increasing number of products in these categories.

The responses requested regulatory harmonisation and coordination, and a call for guidance across a large range of areas was issued, including:

- roles and responsibilities, regulatory pathway and process (for digital therapeutics and biomarkers), bridging studies and follow on test panels for companion diagnostics;
- best practices and harmonising standards in the areas of product quality and design, clinical validation, patient utilization, and regulatory approval oversight of novel digital therapeutics.

They requested that joint scientific advice, involving all relevant stakeholders be available for these products and that regulators ensure that appropriate expertise is available. Horizon scanning was welcomed, with more information on its implementation sought.

1. **Develop understanding of, and regulatory response to, nanotechnology and new materials in pharmaceuticals**

*Individual member of the public and Patient or Consumer Organisation*

Individual members of the public (N=2) and patient and consumer organisations (N=2) stressed the need for EU level action in nanotechnology. They requested that an appropriate European regulatory pathway be designed.

*Healthcare professional*

Two HCPs responded

*Academic researcher and other scientific organisations*

One academic and three other scientific organisations responded. They welcomed the initiative and outlined the current challenges facing nanomedicines, particularly for follow-on nanomedicines, or nanosimilars. These problems stem from a difficulty in characterising their physico-chemical properties, which in turn makes it difficult to understand how manufacturing or quality changes affect their safety or efficacy, and therefore, what critical quality attributes should be. To remedy this, they requested that the pharmacovigilance system allow for the differentiation between originators and follow-on products. They also suggested a multi-streamed research effort to look at correlations between in vitro, in vivo and clinical outcomes with quality attributes. This could then lead to EMA working with multiple stakeholders to produce guidance for nanosimilar or complex medicines.

*EU Regulatory partner / EU Institution*

EU regulatory partners/institutions (N=3) provided suggestions to support nanomedicines and new materials’ development and regulation.

*Payer*

The two payer responses supported the need for new regulatory models, but stressed that these need to ensure quality, efficacy and safety. Protection of public health remains paramount.

*Pharmaceutical industry*

Individual company and Trade associations

One industry and one trade association responded. They were split on whether or not this should be a priority, despite a range of suggestions being made for improving the regulatory framework for nanomedicines.

1. **Diversify and integrate the provision of regulatory advice along the development continuum**

*Patient or Consumer Organisation*

Two patient and consumer organisations emphasised that early scientific advice needs to be associated with strict conflict of interest rules and increased transparency.

*Healthcare professional organisation*

The two healthcare professional organisations which responded welcomed the opportunity to harmonise scientific advice and ensure the evidence needs of different stakeholders were met. They believed that multi-stakeholder scientific advice could serve to bring dispersed scientific advice under one umbrella and avoid discrepancies.

*Payer*

The two payer responses requested that implementation of this Core Recommendation follow the Ombudsman's recommendation on SA. While consistency was desirable, enough flexibility was needed to adapt to new developments and ensure that a final assessment was not restricted by earlier decisions.

*Pharmaceutical industry*

Individual company and Trade associations

The individual pharmaceutical companies (N=17) and their trade associations (N=7) greatly value existing scientific advice but welcomed the Core Recommendation to further diversify and integrate the provision of regulatory advice along the development continuum. They clearly laid out the drawbacks of scientific advice in Europe: the fragmented, and often, conflicting advice provided by different regulatory bodies which require separate regulatory submissions e.g. clinical trial authorisation bodies, national competent authorities, notified bodies, EMA's different advice pathways and committees, particularly PDCO, COMP, CAT, SAWP and CHMP. This heterogenous landscape makes development very burdensome in the best case and can hamper development of medicines in the worst case. They contrasted this to the FDA IND system which is simpler and more responsive to the needs of the industry.

To remedy the problems in European scientific advice, and deliver the Core Recommendation, they came up with a number of recommendations. For divergences between advisory bodies, they recommended more multi-stakeholder advice to build shared understanding and harmonise opinions, this would have the option to include HTAs, payers, NCAs, notified bodies, the clinical trial facilitation group and others. It would also include more experts from EMA and its Committees. They welcomed an enhanced EMA-FDA parallel advice.

Secondly, the timeframes of drug development are faster than EMA scientific advice, and this difference was seen as creating uncertainty, gaps or pauses in development. The increasing complexity of drug development also means that advice is required more frequently. Currently, the responses stated that EMA's SA was too slow and burdensome. To remedy this, it was suggested to create the option of more informal, rolling advice across the lifecycle, with different levels of regulatory engagement. These should include network expertise, EMA experts, working parties and groups, with the provision of preliminary feedback ahead of scientific advice discussion meeting so that the sponsor can also suggest additional topics for discussion based on this feedback. In the area of CMC, quality and manufacturing were seen as particularly in need of informal, swift input from regulators, across the lifecycle. They suggested making the support offered through PRIME available to all products, with its early appointment of a rapporteur and earlier, flexible advice.

They requested that the clinical trial information system allow better sharing of knowledge across the network to facilitate shared understanding of a given development. They also requested efforts to advance the acceptance of novel methods, such as digital endpoints, which may require expedited, multi-stakeholder advice, due to their role in clinical trials. For specific developments such as paediatrics or drug-device combinations, special treatment was requested.

The responses saw delivery of this Core Recommendation as linked to the following others:

- Reinforce patient relevance in evidence generation
- Contribute to HTA’s preparedness and downstream decision making for innovative medicines
- Promote and invest in the PRIME scheme
- Create an integrated evaluation pathway for the assessment of medical devices, in vitro diagnostics and borderline products

Goal 2: Driving collaborative evidence generation – improving the scientific quality of evaluations

1. **Leverage non-clinical models and 3Rs principles**

*Patient or Consumer Organisation*

Only one patient responded and hence cannot be summarised.

*Academic researcher, European research infrastructure and other scientific organisation*

One academic researcher, other scientific organisation and European research infrastructure responded. They supported the initiative to leverage non-clinical methods and highlighted in silico methods as needing addressing. In addition, they proposed to stipulate incentives for new 3R methods.

*EU Regulatory partner / EU Institution*

EU Regulatory partners (N=5) supported this Core Recommendation. They proposed systematic valuations of predictive values of preclinical models for drug effects in humans.

*Health technology assessment body and Payer*

Two HTA bodies and one payer responded, supporting the initiative.

*Pharmaceutical industry*

Individual company and trade association

Two individual companies and their trade associations responded. They supported the initiative and expressed willingness to collaborate.

1. **Foster innovation in clinical trials**

*Patients’ or Consumers’ Organisations*

Patients’ or Consumers’ Organisations (N=9) were consistent in advocating a focus on the strength, rigor and representativeness of randomised controlled trials (RCTs) rather than innovative clinical trial design.

They requested that EMA do the following: ensure these RCTs include representative subgroups, demand comparative RCTs where possible, require that one of the 2 RCTs for approval be done by an independent party, pool resources across Member States to allow meaningful-pragmatic RCTs responding to questions relevant to clinical practice, discourage surrogate endpoints where final outcomes are achievable within a reasonable timeframe and without harm for trial participants.

Patients’ or Consumers’ Organisations also expressed caution with regards to the use of RWD. They asked that EMA reflect on scope, checks and balances and quality criteria for RWD. EMA should be cautious about using such data to establish clinical effectiveness due to high confounding. Post-marketing evidence generation should focus on adverse drug reactions.

*Healthcare professionals’ organisations*

Four healthcare professionals (HCPs) and organisations responded. There was no consensus for a summary.

*Academic researchers, European research infrastructures, other scientific organisations and healthcare professionals*

Academia (Academic researchers N=2, European research infrastructures N=2, Other scientific organisations N=9) were generally in favour of innovation in clinical trials, particularly regarding modelling, simulation and extrapolation, and in trials on rare diseases. They felt scientific advice and in-silico trials would facilitate such innovation. In-silico trials, in particular, could help to reduce, refine, and partially replace real clinical trials. They could reduce the size and duration of trials by adding simulated patients that might fill gaps in the individual variability seen in ‘real’ patients. They might also be able to determine and remove those patients that will not respond to the candidate biomedical product or improve safety through pharmacokinetic/pharmacodynamic multimorbidity modelling. Finally, RWD was recommended as a supportive data source for both in silico and RCTs. Several responses highlighted the interdependence of this Core Recommendation with others such as:

- Support developments in precision medicine, biomarkers and ‘omics’
- Create an integrated evaluation pathway for the assessment of medical devices, in vitro diagnostics and borderline products.

*EU Regulatory partners / EU Institutions*

EU Regulatory partners (N= 6) requested that EMA monitors medicines continually, integrating additional information in clinical trials: from administration through pharmacological activity and pathophysiological modification, to impact on disease/symptoms.

*Health technology assessment bodies and Payers*

The HTA responses (N= 4) strongly supported recommendations to ensure that novel practices and procedures facilitate HTA acceptance and patient access.

Two payers considered that many modern trial designs are too flawed or at risk of bias to use in anything beyond exploratory clinical trials. They also requested that surrogate endpoints only be allowed when they have been validated as impacting clinically meaningful endpoints.

*Pharmaceutical industry*

Pharmaceutical companies (N=17) and their trade associations (N=7) supported innovation in clinical trials. They repeatedly requested that EMA organise dedicated multi-stakeholder collaborations (e.g., workshops, demonstration projects and pilot schemes) to raise awareness, share case studies, and identify best practice in innovative clinical trials.

On top of this they requested that EMA create a forum to resolve alignment issues across National Competent Authorities, ethics committees, HTA bodies and patients’ organisations when considering acceptance of complex and/or seamless clinical trials. This could be complemented by a complex clinical trial strategic initiative including multiple stakeholders, to agree standards which can be used as a basis for international harmonisation through the International Council of Harmonisation (ICH). They stressed that the EU will lose attractiveness as a place to conduct clinical research if innovative clinical trials are not fostered and EU stakeholders do not better align the clinical trial pathway.

Some specific actions came up, for example, to further develop the CT Information System (CTIS) to best accommodate complex clinical trials. The CTIS should be able to efficiently manage applications for, and the datasets arising from, complex clinical trials. Earlier timing of advice would facilitate parallel scientific advice from EMA/FDA. Responses proposed that EMA consider harmonising and developing guidance in biomarkers and endpoints, particularly digital biomarkers and patient reported outcomes, and looking into how the orphan medicines regulation will adapt to tissue agnostic indications.

The responses linked this Core Recommendation with the implementation of these others:

- Develop the regulatory framework for emerging clinical data generation
- Support developments in precision medicine, biomarkers and ‘omics
- Create an integrated evaluation pathway for the assessment of medical devices, in vitro diagnostics and borderline products
- Reinforce patient relevance in evidence generation
- Optimise capabilities in modelling, simulation and extrapolation
- Promote use of high-quality real-world data (RWD) in decision making

1. **Develop the regulatory framework for emerging clinical data generation**

*Patient or Consumer Organisation*

Patient and consumer organisations (N=4) were positive about developing a regulatory framework for emerging clinical data generation. They believed digital data has a lot of potential, particularly in clinical trials. A regulatory framework is needed, however, to clarify data privacy and security, as well as to promote data accessibility for further research.

*Healthcare professional organisation*

Healthcare professionals (N=3) thought the creation of a regulatory framework for emerging digital data generation was appropriate.

*European research infrastructure and other scientific organisation*

A European research infrastructure and other scientific organisations (N=5) were positive about a framework for clinical data. However, they felt there was a need to consider its relevance carefully and ensure the capability to analyse this data

*EU Regulatory partner / EU Institution*

EU Regulatory partners (N=4) saw clinical care data as an opportunity to both inform regulatory decision making and to educate patients and HCPs. However, they noted the importance of ensuring that data is relevant and expressed a need to be aware of possible wider effects.

*Health technology assessment body and payers*

HTA bodies (N=3) and payers (N=2) stressed the need for clarity in several areas before methods are developed to allow regulatory decision-making with these novel data sources: the circumstances, products, purposes and advantages of this data, and its quality and standardisation. This is also indicated in the HMA/EMA Task Force on Big Data.

*Pharmaceutical industry*

Individual pharmaceutical companies (N=11) and their trade associations (N=7) were aligned in their support for the development of a regulatory framework for emerging clinical data, pre and post-approval. Some said that because of technical innovation, particularly in ICT and RWD, it is inevitable that this data will be increasingly incorporated into regulatory decision-making and regulators should embrace this.

They requested multistakeholder dialogue in the construction of such a framework, as well as international collaboration with the FDA. This framework should advance the acceptability of digital endpoints and biomarkers, particularly those derived from RWD. It should also be coupled with innovative ways to seek advice from regulators.

1. **Expand benefit-risk assessment and communication**

*Patients’ or Consumers’ Organisations*

Responses from patients and consumer organisation (N=11) reflected strong support for EMA to improve benefit-risk decisions and the way they are communicated; this should remain at the core of the EMA strategy. Regarding benefit-risk decision making, several stressed that EMA should carefully consider the use of accelerated and conditional approvals; the use of these approval types was described as justified in some situations, but needed to be the exception as postponing reassurance about clinical value led to concern about putting patient safety at risk. Several felt that EMA should request comparative RCTs versus standard therapy, using patient-relevant outcome endpoints, whenever possible. This would reassure patients, HTA and payers that a new treatment works better in comparison to alternative options (if any).

Several responses mentioned that benefit-risk assessment should include the evaluation of PROs, patient reported outcome measures, patient preferences and individual patient data to reflect patient’s actual needs and expectations. Several also stressed that EMA should ensure that submitted data answers clinically relevant questions and that regulatory decisions are guided by clearly defined, unmet public health needs. Responses highlighted that EMA should request high-quality post-marketing studies to confirm benefit and resolve uncertainties raised during the initial authorisation, which was seen as especially important when medicines are authorised on less comprehensive data. Some responses also expressed the view that post-marketing evidence that does not confirm benefit should inform the withdrawal of medicines. Similarly, several stressed that EMA should ensure pharmacovigilance activities remain a priority, again, especially in view of medicines arriving on the market before comprehensive data on its safety and efficacy can be gathered.

Regarding benefit-risk communication, EMA should ensure clear, sufficient and transparent communication of the benefits and risks, specifically of products approved via accelerated procedures and conditional marketing authorisation; patients, HCPs and prescribers should be fully aware of the benefits, risks and how they compare in order to make informed treatment decisions.

*Other scientific organisations*

All three responses were very supportive and suggested some specific actions. They emphasised the need to build on existing good practice guidance (such as that issued by ISPOR, the FDA and the results of IMI PREFER).

*EU Regulatory partners / EU Institutions*

All responses (N=10) stressed the importance of these actions, stating that benefit risk assessment is the core competency in regulation. Improving the consistency, transparency and predictability of benefit-risk decisions was found to be necessary to ensure that they do not become less meaningful for subsequent decision makers and patients. Many recommended establishing a process for continuous monitoring of benefit-risk after initial approval incorporating RWE, thereby moving away from single time point approval.

*Health technology assessment bodies*

Of the five responses, many welcomed better communication and/or collaboration with payers and specifically welcomed deepened discussions on unmet medical need, severity of disease, existing treatment options, suitable comparators and outcomes comparison vs. placebo/active-control (including size in effectiveness in absolute terms) and patient perspectives.

*Payers*

Payers (N=5) largely echoed HTA bodies` feedback. All payers strongly supported better communication and/or collaboration amongst payers and HTAs. They specifically welcomed deepened discussions on unmet medical need, severity of disease, existing treatment options, suitable comparators and outcomes comparison vs. placebo/active-control (including size in effectiveness in absolute terms) and patient perspectives.

Regarding communication, several stressed that EMA should publicly explain its decisions and provide insights into the benefit-risk balance and warning against possible harms, so that patients and down-stream decision makers are clearly informed about the reasons behind decisions and side effects. Two stated the need: i) for more detailed descriptions of remaining uncertainties of the benefit-risk assessment and ii) to clarify that HTA bodies/payers and EMA have different responsibilities and methodological requirements.

Regarding patient preferences, a number stated that the incorporation of patient preferences should happen in a methodologically sound, transparent and impartial way with clear rules for conflict of interest. Some responses mentioned that actions regarding preferences should consider the methodological challenges of eliciting patient preferences: for example, preference studies are too often misleading as preferences change with experience with illness and become less precise with increasing complexity of decisions, and such studies tend to not elucidate the whole picture.

*Pharmaceutical industry*

*Individual companies*

Individual company responses (N=5) overall supported the actions related towards expanding benefit-risk assessment and communication via the incorporation of patient preference data. They expressed support for improving communication, particularly to HTA bodies. However, two responses diverged as to whether the capability to analyse Individual Patient Data was the best use of regulatory resources.

*Trade associations*

Overall, trade associations (N=5) supported the actions related to expanding benefit-risk assessment and communication. Two stressed the importance of having the support and alignment among all concerned stakeholders on actions related to methods for structuring benefit-risk assessment, communication and patient preferences.

1. **Invest in special populations initiatives**

*Patient or Consumer Organisation*

Responses (N=7) reflected strong support for this recommendation. Several responses encouraged EMA to ensure that all different patient populations are considered in the RSS. Specifically, some pointed out that elderly with multiple morbidities are often a neglected patient population in clinical development and evaluation; responses argued these special populations should be better represented in clinical trials, benefit-risk assessment and communication. A number specified how RWD/RWE could be used to counteract the knowledge gap in these populations (see actions below).

*Other scientific organisation*

Scientific organisations (N=5) strongly supported increased attention towards special populations. Some argued that EMA should consider how to incentivise sponsors to develop medicines in special populations, to counter a current lack of financial incentives for industry. Some stated that EMA should support and engage in multi-disciplinary approaches such as public-private partnerships and consortia; these were described as offering an effective mechanism for consolidating resources to advance efforts in special populations, but needed funding.

Several scientific organisations specified how RWD/RWE as well as simulation and modelling tools could be used to address the knowledge gap in special populations. Two also noted that EMA should pay attention to the development of “proxy PROs” (i.e. observer-reported outcome measures) that are completed on behalf of study participants who are unable to communicate their experiences, such as parents acting on behalf of neonates. These responses stated that EMA should also increase efforts to further the science about mechanisms and natural history of many neonatal conditions. They further suggested that EMA should work together with other regulators to ensure rigorous and stringent regulatory science while meeting important therapeutic needs in this area in a timely manner.

*EU Regulatory partner / EU Institution*

No consistent themes emerged among the heterogeneous responses (N=3).

*Health technology assessment body*

No consistent themes emerged among the heterogeneous responses (N=2).

*Payer*

The two payers answering this question acknowledged the necessity of efforts to address special populations, including the value of supplementing clinical trial data with clinical care data. However, they both felt that EMA should consider that the proposed adaptive approaches for iterative development result in a risk shift from pre-marketing to post-marketing, and that this may result in a larger population at risk instead of the intended risk minimisation. Both also underlined that the use of clinical care data should not replace the need for clinical trials.

Comments from the two payers also reflected hesitance towards modelling and simulation approaches; they both stressed that these approaches should not apply to all products as they argued that such models, as applied to paediatric populations, already have demonstrated limitations. The two payers further stated that extending modelling and simulation to areas other than paediatric populations, such as to biosimilar development, is difficult to justify and needs to be corroborated with significant evidence (“striking and representative examples”). Like the HTAs, the two payers asked that it be specified when modelling and simulation approaches would be used and, when they should replace clinical trials.

*Pharmaceutical industry*

Individual companies

Individual companies (N=5) acknowledged the importance of this recommendation and agreed that an adequate representation of certain patient groups, including children, pregnant women and older adults in clinical research is a current challenge. Some also reported that narrow eligibility criteria add to this issue. Several asked EMA to continue its current efforts (e.g. the European Commission’s joint evaluation of paediatric and orphan regulations, IMI conception) focussing on this recommendation. Echoing answers from the above stakeholder groups, several argued that this recommendation was linked to implementing other recommendations in this strategy, e.g. modelling and simulation, adapted clinical design, real world data, use of wearables, registries to generate data from these patients.

Trade associations

Like individual companies, several trade associations (N=4) saw this recommendation as an important objective already being pursued by EMA, and expected EMA to continue its ongoing efforts (e.g. EC joint evaluation of paediatric and orphan regulations, IMI conception). Echoing other stakeholder groups, some also argued that specific populations such as elderly, pregnant women and rare disease patients face high unmet needs and deserve EMA’s focus.

1. **Optimise capabilities in modelling, simulation and extrapolation**

*Individual member of the public, patient or consumer organisation and a healthcare professional organisation*

Very few responses were received from a member of the public, a patient or consumer organisation and a healthcare professional. International harmonisation of methods and standards was highlighted as important.

*Academic researcher, European research infrastructure and other scientific organisation*

Academic researchers (N=5), a European Research infrastructure (N=1) and Other Scientific Organisations (N=5), supported the initiative. Some went so far as to call for a modelling and simulation revolution as has been seen in other industries e.g. aerospace and energy, and to even replace clinical trials. They stressed the importance of modelling, simulation and extrapolation to advancing the 3R agenda and for studying special populations and rare diseases. Modelling simulation and extrapolation were thought to require information sharing amongst stakeholders and its delivery was seen as interlinked with AI, 3Rs and innovation in clinical trials. Education and training were also noted as important in this area.

They were particularly favourable towards in silico modelling, calling for a specific goal within the strategy to be dedicated to it. In silico modelling was seen missing in the strategy document, and important to pharmacovigilance where it can be used to assess single ADRs, for example. Nevertheless, they recognised that despite the benefits, the challenges and safety of in silico modelling needs to be carefully thought through.

*EU Regulatory partner / EU Institution*

EU Regulatory partners (N=5) suggested that the evidence to implement this Core Recommendation. should be collected from existing EU projects and then applied, across the totality of the drug lifecycle.

*Health technology assessment body and payer*

The HTA (N=3) and Payer (N=2) responses agreed on the potential of MS&E. However, Payers were concerned that it would result in the shift of evidence generation from pre-approval clinical trials, to conditional approval and then post-approval real-world data collection. They felt, if MS&E were used inappropriately this shift could pose a risk to a greater population than in clinical trials. Therefore, they suggested the restriction of its use to specific instances. They further called to define the specific cases where M&S could be applicable, as it would be difficult to justify in some cases (e.g. biosimilar development)

*Pharmaceutical industry*

Individual company and trade associations

The industry responses (Individual company N=7, Trade associations N=5) were unanimous in their support for modelling, simulation and extrapolation. They viewed it as useful in advancing medicine development through preventing the unnecessary generation of data, principally avoidable clinical data. They provided examples of the potential of MS&E across the lifecycle, particularly in the CMC area: stability and degradation modelling, manufacturing process modelling for establishing manufacturing approaches, safety evaluation of intermediates and impurities, PK and dissolution modelling for bioequivalence, advancing extrapolation from adult to paediatric populations and post-approval changes based on models built on real-world data. Increased European Network expertise, as well as EU and international regulatory acceptance of these methods was seen as necessary to delivery of these benefits.

1. **Exploit digital technology and artificial intelligence in decision making**

*Individual member of the public and patient or consumer organisation*

The responses (individual member of the public N=1, patient or consumer organisation N=2) expressed caution regarding the use of AI in medicines development and suggested the Agency also take a cautious approach, ensuring its use is properly validated in comparison to existing methods.

*European research infrastructure and other scientific organisation*

These responses (scientific organisations N=3 and a research infrastructure) all welcomed the Core Recommendations but again suggested caution in EMA's application of AI. They requested that EMA produce guidance on AI's applications in medicine development.

*EU Regulatory partner / EU Institution and health technology assessment body*

EU Regulatory partner / EU Institution (N=4) and HTA bodies (N=2) supported the Core Recommendation, stating that it is a topic of increasing importance and therefore it should be monitored. However, despite the majority supporting the underlying action of EMA and the Network applying AI it to their own processes, there were questions as to whether it was appropriate to take a leading role.

*Pharmaceutical industry*

Individual companies (N=9) and trade associations (N=4) welcomed the Core Recommendation. They believed AI use in industry will increase over the next five years and so requested the EMA and the Network to increase its capabilities in AI and narrow the skills gap. Although they welcomed the outcome of the HMA/EMA Task Force on Big Data, they requested more transparency on EMAs thinking in this area through guidance documents and position papers. This guidance should include privacy and data protection issues. They also requested more flexible interaction with the EMA on plans to use digital tools, including for digital endpoints.

New digital data tools allow for a multiplicity of hypothesis generation. A new regulatory framework for studies with multiple outcomes was therefore requested e.g. for post licensing evidence generation. They also wished to see further global regulatory harmonisation in this area.

Responses saw the area as intrinsically linked to other Core Recommendations including those relating to RWD and big data.

Goal 3: Advancing patient-centred access to medicines in partnership with healthcare systems

1. **Contribute to HTA’s preparedness and downstream decision making for innovative medicines**

*Patients’ or Consumers’ Organisations*

Of the ten responses, many agreed that EMA should engage in early discussions with HTA bodies to align (clinical) evidence requirements to close (clinical) evidence gap between HTA and regulatory requirements; several stated that a lack of alignment currently impedes or slows down patient access. They considered this required close collaboration with payers as well as HTA bodies, and the involvement of patients and healthcare professionals. While recognising the different roles of regulators, HTA bodies and payers, a few responses proposed regulatory requirements should be adapted to meet HTA, payer and society requirements, e.g. by making it a regulatory requirement that added therapeutic value be demonstrated, with regulatory guidelines mandating the submission of comparative trial data against standard treatment.

A number of responses highlighted actions for cooperation with HTA bodies, e.g. by inviting HTA experts to CHMP discussions, and the need to anticipate divergences/reimbursement challenges when regulatory concepts do not fit in the reimbursement setting. Regulatory concepts for discussion mentioned in this context were: surrogate endpoints (when the relation between the endpoint and clinical outcome has not been completely established), conditional approval, the population to benefit, significant benefit for orphan medicinal products (mentioned multiple times) and unmet need.

Various responses highlighted that parallel EMA/HTA scientific advice should be strengthened to reduce the risk of inadequate information provided to EMA/HTA at time of evaluation. The European Network for Health Technology Assessment (EUnetHTA) could be used as a platform to exchange information between CHMP and HTA and HTA assessors allowed to have this information in parallel to CHMP evaluation.

*Healthcare professionals’ organisation*

Healthcare professionals’ organisations (N=4) highlighted that EMA should enhance discussions with HTAs regarding HTA guidance and methodologies for evidence generation and review. Some also mentioned that EMA could contribute to identification of priorities for HTAs.

An additional suggestion was that a robust and effective framework was needed for collaborative EU-level HTA assessment in order to streamline regulatory procedures, avoid duplication, shorten time for decision-making, and make the best use of public and private human and financial resources.

*Academic researcher*

Only 2 academics responded to this question. Their responses showed strong support for the proposed actions aiming to strengthen collaboration and alignment of evidence requirements between EMA and HTA bodies. The two researchers proposed various detailed actions towards this goal, notably:

- Contribute to the development a of core outcome sets (COS) together with HTA bodies for use “throughout ecosystem” by regulatory and HTA/reimbursement assessments and decisions; it was proposed that research using these outcomes can be compared, combined and that all studies provide usable information;
- Collaborate with HTA bodies on post-authorisation evidence requirements and introduce EU clinical registries post-authorisation in addition to existing managed entry agreements
- Anticipate that registries will require significant investment in registry design, operating data systems, and training and licencing; the cost of running the registries should be factored in HTA evaluations and discussed on the distribution of costs between the payer and manufacturers.
- HTA bodies could stipulate a resource impact assessment applying the annuity and payment by performance models. This criterion would serve as a tool to predict future expenditure and identify the best reimbursement model early on.

*Healthcare professionals*

The responses (N=2) suggested to align this priority with the adoption and implementation of the legislative proposal on HTA collaboration.

*Other scientific organisations*

Responses (N=3) pointed towards actions to collaborate with and leverage knowledge and experience from HTA bodies. Many noted the value of using an EU-based approach e.g. via EUnetHTA.

*Health technology assessment bodies*

Responses (N=5) supported actions to ensure collaboration between regulatory agencies and HTA bodies.

*Payers*

Payers (N=4) asked that EMA ensure that requirements for HTA/payer processes are already integrated in the pre-authorisation phase; trial designs should reflect the requirements of HTA assessments. Although incorporation of evidence needed by payers and HTA into developments plans was described to be indispensable, and some stated that fulfilment of HTA requirements should be essential for achieving marketing authorisation, others commented that HTA and regulators have different responsibilities and therefore rightfully ask different questions.

Similarly, differences between HTA and EMA assessments were seen as justified and not hindering better cooperation. However, responses asked to better explain these differences in the public domain. Several also asked EMA to clarify what «contributing to HTA priority setting» is supposed to mean. It was highlighted that target parameters should be defined when monitoring the impact of decision-maker engagement. Furthermore, several underlined that while discussion often focuses on access alone, in reality, the triangle of access, affordability and added benefit was stated to be relevant.

*Pharmaceutical industry*

Individual companies

Individual company responses (N=9) all supported continuing collaboration with HTA bodies. Streamlining evidence requirements between regulators and HTA bodies was seen as necessary for ensuring timely access to medicines. Many stated that EMA should ensure broader stakeholder agreement and alignment early in medicines development on the data and evidence to be generated in order not to delay regulatory approval and patient access. Increased focus on opportunities for early dialogue/parallel consultation with all stakeholders was also welcomed. Two responses were more cautious.

Trade associations

Responses (N=4) described that actions should aim to ensure evidence pertinent to regulator, HTA, payer needs, and patients is defined early in medicines development and by incorporating input early and from all stakeholders (including HTA/payers and patients) in medicines development and evidence requirements. This was especially described to be necessary for ATMPs and other areas where innovation puts pressure on the EU system e.g. personalised medicines and medicines for rare diseases. Multiple responses also underlined the necessity of actions needed towards increasing transparency; EMA should make publicly available documents explaining why and how decisions during the approval process were made, e.g. why the agency accepted the trial design, the endpoints for approval, why a given duration of trial was acceptable. This would assist HTAs/payers reviewing submissions at a later date. Two participants described that EMA should help ensure that any limitations in data presented at marketing authorisation are recognised early together with proposals to mitigate any limitations to enable access. This was stated to be important specifically regarding rare diseases that often have small and heterogeneous clinical trial populations; also, here, EMA should ensure input from all stakeholders is incorporated to determine how limited data in some patient populations can be managed to improve patient access.

1. **Bridge from evaluation to access through collaboration with payers**

*Patients’ or Consumers’ Organisations*

Of the responses (N=8), many patient and consumer organisations were positive about adapting regulatory requirements for pre-market evidence so to meet the demands of HTA, payers and society.

*Healthcare professional and healthcare professionals’ organisation*

The 2 suggestions received from HCP organisations were to promote a transparent mechanism that allows payers to recognise the value of new therapies (it was noted these are often not recognised by payers, therefore not reimbursed or used), and to clarify the treatment-eligible patient population included in the labelling, and its scientific rationale.

*Other scientific organisations*

Of the responses (N=7), some expressed support for the continued development of collaboration with HTA bodies, payers (and other stakeholders) across the medicines life cycle. Others, however, felt that EMA should consider the distinct roles of HTA and payers; payers consider not only the HTA evaluation, but also national, economic, political and other public policy considerations (e.g. health priorities) in making their decision. Whereas the added value of EMA-HTA collaboration was said to be clear as it focuses on data assessment, in which the expertise of EMA was welcome, the benefit of EMA-payer collaboration was seen as more limited (e.g. to horizon scanning).

*EU Regulatory partners / EU Institutions*

The two responses suggested specific actions related to the need for EMA exchange more information with HTA/payers to increase their timely preparedness for evaluating medicines for reimbursement.

*Payers*

Payers (N=4) supported the action of creating a single platform for interaction on evidence generation plans so these could satisfy EMA and payer decision-making; the needs of payers had to be reflected early on in the approval process. The EMRN was asked to reflect on establishing a permanent working structure between EMA and payers with relevant objectives, planning and responsibilities.

*Pharmaceutical industry*

Individual companies

Mirroring comments from other stakeholder groups, many of the seven responses from the pharmaceutical industry supported cooperation, including a single platform to enable one evidence generation plan and sharing the rationale for authorising a particular patient population. Responses supported actions to ensure development plans consider all elements necessary to not only demonstrate efficacy and safety but also comply with downstream requirements. However, echoing comments from scientific organisations, many considered collaboration with payers to be more complex than collaboration with HTA; although payer decisions are informed by HTA assessment, the criteria for payer decisions were described to be very different to the clinical assessment undertaken by the EMA.

It was felt that EMA-payer collaboration would be limited by the complexity of the payer infrastructure across Europe; streamlining with national payer decisions could potentially lead to complexity and delay in the regulatory system. Similarly, several responses suggested that initiatives related to payer decision-making should be undertaken by other agencies at the EC and national level as opposed to EMA; it was reiterated that EMA should consider that these actions extend the remit of EMA beyond scientific evaluation into political decision making which was described as a member state government competence.

Trade associations

Of the five responses, many stressed that EMA should leverage payer collaboration to gain insight into their perspectives on unmet needs and priorities; early engagement in turn helps to prepare payers for potential major impacts from breakthrough innovation.

However, in line with comments from other stakeholder groups, several stated that EMA should consider the need to maintain the distinctiveness of regulatory processes and pricing determinations, and for regulators to keep their scientific focus; initiatives to address pricing/reimbursement decision-making should be undertaken by other agencies at the European Commission and national levels.

1. **Reinforce patient relevance in evidence generation**

*Patients’ or Consumers’ Organisations*

There was a unanimous call for greater and systematic patient engagement. With responses (N=12) highlighting the added insights patients bring through living with the disease and taking the medicines. This involvement was requested to span the drug development lifecycle, including clinical trial design with meaningful endpoints such as always incorporating quality of life (QoL) outcomes and PROs, as well as in the development of new and existing guidelines, where again, PROs should be incorporated. It was stressed that these methodologies should be scientifically robust. They also requested EMA to develop a regulatory framework for digital clinical data generation and promote the use of high-quality RWD that includes patient data. Patients highlighted that implementing these measures would aid downstream decision makers such as HTAs; HCPs and, ultimately, patients.

*Healthcare professionals’ organisation*

Healthcare professionals (N=6) viewed patient involvement as a priority, including in evidence generation. They advocated for ensuring endpoints are patient relevant.

*Academic researcher, European research infrastructure and other scientific organisation*

Academic researchers (N=2), a European Research Infrastructures and other scientific organisations (N=4) expressed a strong desire to reinforce patient engagement throughout the lifecycle of medicine development. In particular, they favoured including PROs in evidence generation.

*Health technology assessment bodies and Payers*

HTAs (N=3) and Payers (N=2) welcomed the proposal for systematic inclusion of PROs, and a health-related quality of life PRO measure (HRQoL) to implement in trials and bridge the gap with comparative assessment, so long it is done with a common understanding between regulators, HTAs and Payers. Two suggested reviewing existing HRQoL measures before developing a new one and urged mindfulness regarding conflict of interest in patient engagement.

*Pharmaceutical industry*

Individual companies

Individual companies (N=9) welcomed reinforcing patient engagement, seeing it as a reflection of their own efforts to do the same. They welcomed the inclusion of PROs into the benefit-risk assessment and requested that such data should be included in the labelling. They also requested that the rigor and methods of inclusion of PROs should be collaborative, transparent and harmonised across decision-makers. The framework for digital data generation was seen as an enabler for this Core Recommendation.

Trade associations

Trade associations (N=4) advocated the Agency go further in-patient input, particularly for PROs. They suggested a systematic, whole-lifecycle approach, with alignment across stakeholders, European wide and globally. In developing tools for gathering patient input, they recommend that a collaborative, multi-stakeholder approach, with clearly defined requirements and guidelines.

1. **Promote use of high-quality real-world data (RWD) in decision making**

*Patients’ or Consumers’ Organisations*

Patients’ or Consumers’ Organisations (N=4) broadly acknowledged the added value of real-world data but sounded a note of caution about its use. They wanted clarity as to what could be considered high-quality real-world data and when its use would be acceptable, advocating that it be seen as complementary to clinical trials.

*Healthcare professionals’ organisation*

Healthcare professionals (N=4) viewed RWD as important for medicines evaluation, particularly post-approval. They stressed the need for an appropriate regulatory framework and platform to support collection and analysis of robust and relevant data, and to ensure appropriate governance and compliance with data protection requirements

*Other scientific organisations*

Overall scientific organisations (N= 10) were positive towards RWD. They recognised its value in both pre and post-approval settings for more closely reflecting real-life and enabling the continuous review of the efficacy and safety of approved products. They requested that EMA produce guidance on what and when RWD is acceptable. They also requested that EMA demand transparency in observational studies.

*EU Regulatory partners / EU Institutions*

EU regulatory partners (N= 5) supported the use of RWD, so long as proper evaluation of its use was undertaken, and robust methodologies were developed. RWD should be able to be used for the full range of regulatory procedures and assessments, pre- and post-marketing.

*Health technology assessment bodies and payer*

HTA responses (N=6) and payers (N=2) had mixed views on RWD. Most welcomed its use post-approval, however, there was considerable doubt about its suitability for evidence generation pre-approval. They requested clarity over the regulatory acceptability of RWD methods and how these would impact on marketing authorisations: it should be made clear when evidence generation can be moved to the post-marketing phase and what the justification would be. They also requested the following be addressed:

- data standardisation
- data quality
- registration in publicly accessible databases
- reproducibility
- data ownership
- validated statistical analyses
- transparency on conflicts of interests of interested parties
- data protection

Two responses requested that EMA refrain from using the term ‘real world data’, preferring ‘observational data’ and stating that RCTs should remain the gold standard.

*Pharmaceutical industry*

Individual companies (N=23) and trade associations (N=6) were near unanimous in their support for promoting high-quality RWD in decision-making. They noted the growing potential of RWD, driven by the digitisation of healthcare information and new analytical methods such as AI and modelling.

The responses explained that RWD will streamline evidence generation and assessment, particularly in rare diseases. They requested that EMA launch a strategic initiative to integrate RWD into medicines development. The initiative would involve pilots, capability building exercises, stakeholder engagement via workshops and guidance. It could include both retrospective and prospective case studies and lead to the development of a regulatory training curriculum for RWD to build knowledge and capacity to regulate.

This initiative should provide clarity on the scope and regulatory uses of RWD and involve all relevant stakeholders, including at an international level. They requested the initiative clarify regulatory acceptability of RWD in areas such as label changes and the collection, quality, validation, transparency, security and privacy (including GDPR), analysis, financing, governance and audit of sources of RWD.

There were also suggestions to develop or ensure the use of a common platform for RWD and for EMA, EC and HMA to link electronic health records into a resource. This initiative should build on ongoing work internationally, particularly from the FDA, and on EMAs own work on patient registries: the HMA/EMA Task Force on Big Data and the EMA’s recent publication on “Use of patient disease registries for regulatory purposes – methodological and operational considerations” and include publicly available conclusions based on CCI. As an additional outcome, it would build regulatory experience in the area and so engender trust in RWD, and this would then permit international harmonisation.

The responses suggested that the recommendation was interlinked with delivering the following Core Recommendations:

- Develop network competence and specialist collaborations to engage with big data
- Contribute to HTA’s preparedness and downstream decision making for innovative medicines
- Reinforce patient relevance in evidence generation
- Exploit digital technology and artificial intelligence in decision making
- Foster innovation in clinical trials

1. **Develop network competence and specialist collaborations to engage with big data**

*Patient or Consumer Organisation*

The two patient or consumer organisations supported the initiative and wanted to see the use of data to create more patient-centred treatment.

*Healthcare professional and healthcare professional organisation*

The responses (one healthcare professional and three healthcare professional organisations) noted that RWD can provide supplementary data in the context of the marketing authorisation process and post-market surveillance activities. They supported the use of big data but highlighted the need to put in place a framework to define data quality. Like the patient and consumer organisations, they stressed the utmost importance of guaranteeing the confidentiality and privacy of patient data, and that this therefore requires an appropriate data governance model.

*Other scientific organisation*

Other scientific organisations (N=3) welcomed the initiative but provided specific suggestions and warnings on its use and the makeup of network collaborations.

*EU Regulatory partner / EU Institution*

EU regulatory partners (N=5) supported the big data recommendation and asked for more concrete steps. They welcomed the recognition of the need for expertise and guidance development. They also agreed that this should be done through new connections to stakeholders involved in big data, but clearly stated that this initiative should involve the network as a whole.

*Payer*

The two payers who responded focused on RWD. They requested that the term ‘RWD’ be replaced with ‘observational data’ which they considered more representative of the data and less promotional. They suggested caution in the application of RWD, particularly pre-approval, and emphasised that in their view RCT must always remain the gold standard. They also requested clarity on why and where RWD can be used in the lifecycle of a medicine together with assurances on data standardisation, quality, registration in publicly accessible databases, reproducibility, validated statistical analyses and transparency regarding conflicts of interest. Finally, they noted that data protection and ownership need to be clarified.

*Pharmaceutical industry*

Those members of Industry (N=4) and their trade associations (N=3) responding to this point supported the Core Recommendation, in particular the need for EMA to invest in skills, capacity and networks of big data. They requested that the implementation include the outcomes of the HMA/EMA Task Force on Big Data and align with other international regulators. In implementing this, they suggested adopting a common data platform and identifying criteria for RWD for decision making.

1. **Deliver improved product information in electronic format (ePI)**

*Patient or Consumer Organisation*

Patient and consumer organisations (N=4) supported the Core Recommendation, not only for facilitating electronic dissemination but also improving the content of the PIL. Particularly enabling it to be more up-to-date, accessible, tailored and interactive. However, there was a suggestion of a bottom up review of the PIL to increase patient involvement in labelling. Specifically, through more patients commenting at an earlier stage and more flexibility for incorporating these comments into the leaflet, as well as implementing systematic user testing.

*EU Regulatory partner / EU Institution*

The four EU regulatory partners who responded were supportive of the Core Recommendation. However, they asked that it goes further in adding functionality for new uses.

*Payer*

Only one Payer responded and hence cannot be summarised.

*Pharmaceutical industry*

Individual companies and trade associations

Individual companies (N=5) and their trade associations (N=4) welcomed the Core Recommendation. They saw ePI as benefiting industry, regulators, patients, carers and HCPs. they requested clarity on the framework for governance and to ensure access through standardised interface. They were divided on whether paper-based formats should be phased out. This is due to the burden they place on industry, with requirements and implementation differing between MS. This heterogeneity is also exacerbating supply issues, for example preventing cross border supply.

1. **Promote the availability and support uptake of biosimilars in healthcare systems**

*European research infrastructure*

The contributor believed that patient organisations should be included in the development of strategic communication to reinforce trust in biosimilars, as an extension of Responsible Research and Innovation (RRI) policies, instead of designing them “for” the patient organisation.

*Other scientific organisation*

The two responses were heterogenous, but overall were not positive towards promotion of biosimilars.

*EU Regulatory partner / EU Institution*

EU regulatory partners (N=3) generally endorsed the recommendation, particularly with regards to communication to healthcare providers and patient organisations to reinforce trust and confidence in biosimilars.

*Health technology assessment body*

Both participants seemed supportive of the recommendation and actions. One felt that the availability and uptake of biosimilars was crucial for cost-containment. They welcomed the development of guidelines on exchangeability of biosimilars and appreciated clarification on the differences, even within different batches of the same brand medicine. The other contributor believed that the paper form of the package leaflet needs to remain in addition to the digital format to ensure that patients have access to necessary information on biosimilars.

*Payer*

Both payers fully supported the recommendation and stated the importance of biosimilars in reducing the costs of medicines whilst ensure safe access to patients. They agreed with the action to develop strategic communication while ensuring biosimilar authorisation is kept at a high standard. They mentioned that guidelines on the exchangeability of biosimilars are welcome.

*Pharmaceutical industry*

Individual company

Of the three pharmaceutical companies who responded, many did not consider this recommendation as a regulatory scientific topic and stated that this recommendation furthermore does not fall within EMA’s remit. This recommendation should be undertaken by National agencies. This recommendation was seen as a low priority.

Trade associations

There were mixed views on this recommendation amongst trade associations (N=3). Most contributors proposed to further develop the biosimilar framework based on scientifically appropriate approval standards with efficiency gains and robust pharmacovigilance measures that put patient safety first. However, it was also considered that this recommendation is not a regulatory science topic.

1. **Further develop external engagement and communications to promote trust and confidence in the EU regulatory system**

*Patient or Consumer Organisation*

Pursuing this recommendation was seen as necessary by all responses (N=5). The key factors identified in several responses as being needed to maintain European citizens’ faith in the regulatory system were: i) the independence of the EMA from commercial interests and ii) more public understanding via transparency and better external communication and information.

Regarding the first point, two responses asked EMA to consider strengthening conflict of interest rules, including for external experts, particularly in view of proposals to increase scientific advice and early relations with drug developers. Many responses noted that the perception of the Agency’s independence and integrity are as important as the reality itself and that to this end, the Agency’s must work proactively to dispel any fears about regulatory capture.

Regarding the latter point, multiple responses argued that the EMA needs to continue its efforts to make data from pre and post-authorisation clinical studies publicly available, including the timely reporting of summary results of all trials in the EU Clinical Trials Register by sponsors. Two responses further argued that EMA should use post-authorisation data to promptly recall the marketing authorisation when expectations were not met. The same responses argued that EMA needs to dispel any mistrust due to links with the pharmaceutical industry by punishing companies who do not bring relevant evidence or who do not report post-authorisation studies on time.

Several points were made concerning the need to ensure medicines information was as clear and understandable as possible for non-technical readers, with a particular focus on the need to improve the package leaflet for patients – it was suggested this should be an EMA priority, and that the Agency must ensure that the development of electronic product information does not come at the expense of improvements in the layout and content of the existing document. Improved user testing incorporating more end-user/patient feedback was seen as key.

*Other scientific organisation*

The two responses took opposing views; hence a summary is not possible.

*EU Regulatory partner / EU Institution*

Three responses were heterogeneous in specific actions proposed, though all showed support for enhancing external communication.

*Health technology assessment body and payers*

Partly duplicating comments, the responses (HTA N=3; payers N=4) strongly agreed with the need to strengthen trust and confidence in the EU regulatory system, underlining that trust is best built on reliability and transparency. To increase trust among stakeholders, they both argued against the observed trend to over-utilise fast track approvals, as they found these approvals inherently increase uncertainties.

*Pharmaceutical industry*

Individual company

All five responses acknowledged the importance of trust and communication. Many argued however that this recommendation should not be viewed as stand-alone activity but rather as a key enabler to deliver other recommendations and the strategy in general. As with other stakeholder groups, two considered there to already be high trust in the regulatory system. Two noted the importance of raising awareness of the activities and role of the EMA via more communication with the public and patients to increase their understanding and trust in the regulatory process.

Trade associations

Like responses from individual companies, many of the four responses noted that actions to increase this should be undertaken in relation to other recommendations, rather than as a separate specific activity. Nevertheless, they underlined the importance of trust and communication.

Goal 4: Addressing emerging health threats and availability/therapeutic challenges

1. **Implement EMA’s health threats plan, ring-fence resources and refine preparedness approaches**

This Core Recommendation received too few comments to generate detailed summaries.

*Payer*

The two Payers supported the initiative.

*Pharmaceutical industry*

The two industry responses and one trade association provided different suggestions for EMAs health threats activities.

1. **Continue to support development of new antibacterial agents and their alternatives**

*Patient or Consumer Organisation*

Due to increasing resistance to antibacterial products and alternatives, a few patient and consumer organisation responses (N=4) agreed that new antibacterial agents are needed and that the business model needs to change or a new one needs to be created. There was strong support of evolving regulatory guidance on the development of antibiotics and improved information on evidentiary requirements.

*Healthcare professional organisation and healthcare professionals*

Both responding HCP organisations supported the action to evolve regulatory guidance and find alternative approaches for both new antibacterial drug development and treatment of infections as a step forward in the fight against AMR.

*Other scientific organisation*

The majority of the responses (N=8) strongly agreed that the development of alternative approaches to new antibacterial drug development was an urgent priority which would require flexible regulatory frameworks. A few examples of different surveillance systems were suggested as approaches to combat AMR that need to be implemented by governments, such as antibiotic stewardship programs and sentinel surveillance systems.

*Payer*

In view of the increasing antimicrobial resistance, the development of new antimicrobial agents was seen as a key action by the payers who responded. However, both considered that the advance of new business models and vaccination decisions falls outside the remit of EMA.

*Pharmaceutical industry*

Individual company

The companies (N=3) considered development of new antimicrobials as a high priority for them and welcomed the proposal for action to combat AMR.

Trade associations

In order to address AMR, all trade associations (N=4) agreed with all the underlying actions proposed in the RSS to 2025. Most welcomed proposals to support the development of new medicines to combat AMR and to work with HTA bodies to define and explain the relevance of evidence requirements for new antibacterial medicines. Furthermore, a majority noted the importance for development of better diagnostic tools such as Point-of-Care (PoC) diagnostics and suggested to also include self-tests in order improve stewardship and limit diseases.

1. **Promote global cooperation to anticipate and address supply problems**

*Individual member of the public*

Only one individual responded, hence a summary could not be made.

*Patient or Consumer Organisation*

Of the five responses from Patient and Consumer Organisations, most reiterated that every Member State is faced with increasing shortages of medicines which harms patient access. The majority of responses also stated that EMA should play a more active role in addressing supply problems in collaboration with Member States, patient and consumer organisations, healthcare professionals and industry. A few contributions urged EMA-HMA to continue promoting a harmonised approach among Member States on how medicine shortages should be communicated within and between the countries.

*Healthcare professional and Healthcare professional organisation*

The two responses from Healthcare professionals (HCP) and HCP organisations welcomed EMA’s intention to work on the promotion of global cooperation to anticipate and address supply challenges. They made clear that the problems caused by medicines shortages is a topic of considerable concern to authorities, patient and consumer groups, healthcare providers and the pharmaceutical industry itself; shortages have far reaching consequences for European health systems.

*Other scientific organisation*

Amongst the three responses, it was agreed that promoting global cooperation to address supply problems and shortages is key, particularly in relation to divergent regulatory approaches. EMA was encouraged to foster harmonisation in regulatory science initiatives and approaches between major health authorities and alleviate supply issues resulting from different languages. In particular, EMA should explore opportunities for harmonising global standards relating to the traceability of medicines in order to avoid any unintended, negative impacts on the supply chain, whilst preventing falsified, substandard, and adulterated products from entering the supply chain.

*EU Regulatory partner / EU Institution*

In addition to manufacturing and distribution issues, most of the four responses agreed that another key reason for shortages, and lack of patient access, is the decision by companies to not market their products in certain EU Member States. Currently, there is no regulatory tool to handle this issue and this creates healthcare inequalities amongst EU countries. There is also a need to enhance collaboration with WHO and through the widening of Mutual Recognition Agreements (MRAs), not only in the area of supply disruptions due to manufacturing quality issues, but also other areas such as commercial issues.

*Payer*

Both responses stated that more information would be desirable on how regulatory decisions could influence products being marketed and the extent to which manufacturing capacity issues result in shortages.

*Pharmaceutical industry*

Individual company

Of the seven responses from individual companies, most acknowledge the complexity associated with the causes of shortages, which go beyond manufacturing aspects, and are based within a global supply chain. They were also supportive of the underlying actions outlined. Several mentioned the need to ensure harmonisation with regulatory bodies at a global level (including WHO and FDA) as this was considered essential to the success of these measures. Examples mentioned included common standards for manufacturing requirements, and increased confidence in international standards of ensuring GMP. Separately, a few participants specified that country specific labelling and packaging can impact packaging lines and jeopardize supply chain integrity. It was suggested to link this recommendation to electronic product information ePI and further increase flexible electronic and digital labelling in the supply chain to better address the causes for unavailability of medicines.

Trade associations

All of the 8 responses from trade associations strongly supported EMA’s objectives to increase the availability of medicinal products in the EU and address the complex, global causes for unavailability. The majority of trade associations agreed with the underlying actions to enhance collaboration with WHO in the area of supply disruption and promote greater knowledge exchange with international stakeholders on shortages due to manufacturing quality issues. A strong dialogue and concerted efforts with all key stakeholders (including EC, EMA, HMA and WHO) was considered a requisite to finding solutions, including steps towards harmonising and streamlining global regulatory standards, and the extension of mutual recognition agreement (MRAs) between EU and non-EU countries to cover all aspects of inspections (GMP, GCP and quality testing). It was further recommended that optimisations of the current regulatory framework for the lifecycle management of medicinal products would improve availability of medicines; The current framework needs to develop to better incorporate scientific and technological advancement and ensure operational efficiency. Such changes have the potential to cut manufacturing delays and lessen supply issues.

One key area which was suggested to be linked to this recommendation, is the replacement of paper with electronic product information (ePI), as this offers a flexibility within the supply chain and therefore could reduce the causes of unavailability of medicines. The recommendation to facilitate novel manufacturing technologies could also be associated to this recommendation as this could increase capacity, particularly for vaccines.

There was strong support to the deliverables of the HMA/EMA Task Force on availability of medicines. This pilot phase was considered as vital to enable industry to adapt internal systems to guarantee compliance and for both regulators and industry to test the requirements highlighted in the guidance and be able to propose area for improvements, where necessary.

More transparent mitigation plans for regulators and MAHs were seen as requisites for firm and risk-based decisions when urgent actions are required in the supply chain, such as non-compliance of manufacturer.

Finally, potential regulatory barriers that could contribute to shortages should be avoided; Regulatory science should contribute to a more scientific/risk-based approach to Environmental Risk Assessment (ERA), instead of applicability to all products, independently of their impact on the environment.

1. **Support innovative approaches to the development, approval and post-authorisation monitoring of vaccines**

*Patient or Consumer Organisation*

In nearly identical responses, both patient/consumer organisations felt an action should be added on improving communication and transparency on the safety and efficiency of vaccines in order to improve public trust in the system.

*Healthcare professional organisation*

Both HCP organisations agreed with the action to tackle vaccine hesitancy. They believe that HCPs and their organisations need to be involved, together with public authorities, in order to deliver facts based on scientific evidence and increase public awareness of the benefits of immunisation and address the mistrust.

*Other scientific organisations*

The four responses gave support to some elements of this recommendation.

*Payer*

Both Payers agreed that development of new means to tackle microbial infections was essential in view of the increase in AMR and was key to future healthcare provision. However, they stated that business models and vaccination decisions fell outside of EMA’s remit.

*Pharmaceutical industry*

Individual company

Nearly all four individual companies believed that EMA was well-positioned to support innovative approaches to development, approval and post-authorisation monitoring of vaccines. Many believed that the actions listed would advance innovative development in vaccines. Furthermore, they fully supported EMA’s role in proactively engaging with key stakeholders to tackle vaccine hesitancy. In order to rebuild public trust, some also recommended to:

- develop local networks for dissemination of information
- create communication tools/strategies which can be deployed across a range of channels

Closer engagement with other international regulatory bodies such as WHO, FDA, should be sought to promote international alignment. A revision of the current EU variation should be looked into in order to keep up with the fast-paced clinical landscape.

Trade associations

Many of the trade associations (N=9) supported the recommendation and its underlying actions and agreed that EMA has a critical role to play in enabling new vaccines to developed and accessible for patients in need. Establishing an EU-platform for benefit-risk monitoring of vaccines post-approval was seen as a key action and it was suggested that an assessment of the IMI’s ADVANCE and DRIVE programmes could support this effort.

Many stated that industry should be included as a key stakeholder in proactive communication to tackle vaccine hesitancy and links to recommendation 3.8 on promoting trust and confidence should be implemented. Furthermore, cooperation between regional and national surveillance networks was crucial to generate accurate and science-based information on the benefit-risk of vaccines for the public.

Engagement with public health authorities and NITAGs (National Immunisation Technical Advisory Groups) to better inform vaccine decisions was highlighted as another important priority. Involvement of HTA bodies should be considered as equally important to better inform decision making. Furthermore, as parallel CHMP/HTA scientific advices has been shown to be useful, the possibility of involving NITAGs in this should be further explored. Promoting innovative clinical trial design allowing to demonstrate positive benefit/risk with a reduced number of subjects in phase 3 is key to deliver new vaccines quicker to the patients.

1. **Support the development and implementation of a repurposing framework**

*Other scientific organisation*

Most of the four responses were positive regarding pursuing this recommendation.

Two organisations argued that incentives must be found for sponsors to pursue label expansions and two recommended exploring how RWD can be used to enable this recommendation (see actions proposed below).

*Payer*

The two responses were similar to those from the HTA body, the two payers responding to this question noting that repurposing is a promising field for further support and referring to ongoing discussions on this topic in STAMP. Again, both stressed the importance of avoiding higher prices as a result of repurposing (see additional comments below).

Both further stressed that any potential legislative changes associated with the implementation of a repurposing framework should not lead to different data standards for repurposing versus applications for extensions of indications under the usual regulatory path: the quality of observational data supporting repurposing might be mixed.

Finally, like the HTA response, both argued that a better definition of EMA’s role in this topic should be provided.

*Pharmaceutical industry*

Individual company

Individual companies (N=4) had split views regarding this recommendation; two were very positive and argued it would lead to efficient drug development (e.g. lower costs, re-use of data, accelerating drug development) that focused on unmet needs. In contrast, two others were very critical and considered that repurposing was already possible in the existing regulatory framework via the development and subsequent approval of medicines for new indications addressing an unmet need; it was unclear what additional actions needed to be addressed by this recommendation.

Some responses highlighted that actions related to RWE could be used to enable drug repurposing via identification of new indications and two responses referred to the same RWE study by Brigham & Women’s Hospital (BWH) that demonstrated CV risk reduction in patients unable to take ACE inhibitors as a good example of using RWE to identify new indications.

Trade associations

The three trade associations gave heterogenous responses; hence they could not be summarised.

Goal 5: Enabling and leveraging research and innovation in regulatory science

1. **Develop network-led partnerships with academia to undertake fundamental research in strategic areas of regulatory science**

*Individual member of the public and patient or consumer organisation*

Responses (Individual member of the public, N=1, patient or consumer organisation, N=2) supported the initiative and requested patient involvement and recognition in research.

*Healthcare professional organisation*

One healthcare professional responded and encouraged the network to support independent research and development of links between academic researchers and healthcare professionals, who are best placed to provide RWD, in particular in post-market surveillance.

*Academic researcher and other scientific organisation*

Academics (N=3) and other scientific organisations (N=9) strongly supported the strategic goal. They viewed it as necessary to ensure evidence-based decision-making. Several responses requested that these partnerships be multi-disciplinary and include all stakeholder groups, including patients and HCPs. Public-private partnerships (PPPs) were suggested as a method to do this, particularly if they are involved in pre-competitive research. Responses said that these partnerships should prioritise existing research networks. They also asked for them to be broad in their scope: not only conducting clinical research but involved in training, improving the quality of expert scientific advice, and data sharing. They also saw such partnerships as useful for increasing regulatory knowledge across stakeholders.

*Health technology assessment body and payer*

Responses (one HTA and two payers) called for transparency around any such initiative with a clearer definition of the funders; they also requested that HCPs, payers and patients be involved. Like academia and scientific organisations, they felt the initiative should include clinical research.

*Pharmaceutical industry*

All but one of the companies (N=6) and their trade associations (N=4) were supportive of this recommendation. They requested that it include all stakeholder groups including industry and students in the partnerships, so that partnerships do not become a silo between regulators and academia. (The divergent respondent felt that the recommendation was already covered by other proposals in the strategy.)

1. **Leverage collaborations between academia and network scientists to address rapidly emerging regulatory science research questions**

*Patient or Consumer Organisation*

The two responses were relatively supportive of the recommendation.

*Healthcare professional organisation*

Two HCP organisations agreed with the recommendations and the importance of increasing scientists` roles in the regulatory science framework. One suggested also involving learned societies as they are able to support EMA in disseminating information and provide a platform for discussion with relevant medical experts.

*Academic researcher and other scientific organisations*

Responses (five academic researchers and five other scientific organisations), largely agreed with the recommendation and highlighted the importance of its underlying actions. Some responses thought that the recommendation and its underlying actions would help develop the academic community and EU network scientists engaged in regulatory science and innovation. It was also felt this would significantly and rapidly increase the regulatory knowledge base. Increasing and evolving new regulatory science education initiatives to educate the next cohort of regulatory science researchers and further develop present regulatory scientists was seen as essential

A single contribution which was repeated by 3 academic researchers and 2 other scientific organisations focused only on in-silico trials and proposed a few actions within this domain:

- EMA must play a central role in dedicated research on in-silico trials and regulatory science may be required, possibly through Horizon 2020
- As in-silico trials use will increase in the future, global alignment amongst international regulators should be pursued.
- As there are currently a lack of specialist skilled in the “technologization” of the regulatory process of in-silico trials, there will be a need to ensure adequate training opportunities for network scientist and academic in the future

*European research infrastructure*

This recommendation and its actions were fully supported by European research infrastructures (N=3), particularly the actions with regards to actively engaging in training early-career researchers in regulatory science.

*Other scientific organisation*

Responses (N=3) largely agreed with the recommendation and highlighted the importance of its underlying actions. Increasing and evolving new regulatory science education initiatives to educate the next cohort of regulatory science researchers and further develop present regulatory scientists was seen as essential.

*EU Regulatory partner / EU Institution*

All three responses agreed with the recommendation and its underlying actions, emphasising their importance in developing the skills and tools needed for the network to respond to regulatory questions and strengthen regulatory knowledge within the EU. This could facilitate the translation of tangible research into new products. Collaboration between academia and regulatory network scientist was seen as crucial in order to ensure alignment in future strategies and objectives within regulatory science research.

*Pharmaceutical industry*

Individual company

Nearly all seven individual companies agreed that EMA should leverage collaborations between academia and network scientists to address rapidly emerging regulatory science research questions and several mentioned there is a good track record of such collaborations within Europe, such as ECRIN. In addition, some responses suggested that beyond academia, EMA should also consider engaging with sponsors, data vendors, patients, professional and clinical societies, but also regulators and government agencies in a cross-institution setting for the advancement of regulatory science. This would allow further knowledge sharing amongst stakeholders.

Several welcomed the focus on ring-fencing investment for up-and-coming scientific challenges but suggested that industry researchers should also be involved as they can support EMA and academia with emerging innovations.

Trade associations

All four trade associations supported the recommendations and welcomed the actions linked. Echoing comments from individual companies, many believed that involving only network scientists and academia to provide translation from applied research into new drug products and regulatory tools would be too narrow, and that industry research should also be involved to support EMA and academia.

1. **Identify and enable access to the best expertise across Europe and internationally**

*Individual member of the public*

The four responses reflected general support for this recommendation. Two highlighted that patients can also be considered experts and could be explicitly thought of in this recommendation.

*Patient or Consumer Organisation*

Different aspects were highlighted by the three responses.

*Pharmaceutical industry*

Trade associations

Of the two responses, no particular additional actions were suggested, however, they were supportive.

1. **Disseminate and exchange knowledge, expertise and innovation across the network and to its stakeholders**

*Individual member of the public*

Both members of the public stated that patients should be part of any decision-making structure.

*Other scientific organisation*

At a time of rapid advances, some of the seven responses suggested that a form of integrated knowledge transfer was required in order to close the gap between scientific, regulatory, practical and industrial practice. This could be achieved through better dialogue amongst developers, regulators, manufacturers and specifically academia as academics are often not aware of the regulatory requirements e.g. GMP, validation etc.

A few responses recommended that a comprehensive and dedicated approach to sharing data across the network should be included in the deliverables. Multi-stakeholder partnerships (RSNN or CORS) could provide a powerful platform to actively engage in data-sharing and could encourage open exchange of data and knowledge. A top priority for EMA should be to clearly define, support and instruct knowledge holders on how to meaningfully share their data and information.

*EU Regulatory partner / EU Institution*

Most of the 6 responses considered that educating and involving healthcare professionals is essential in the dissemination and exchange of knowledge of new drugs developed. This would reduce the gap between regulators, scientific and healthcare communities which is currently increasing due to more complex products and regulation, and would help ground regulatory guidelines in practicalities. Furthermore, medical professionals will need to contribute more to post-registration evidence and thus regulators will need to ensure sufficient training is available.

*Pharmaceutical Industry*

Individual company

The three contributors largely agreed with the recommendation and suggested the industry should be involved in the exchange of knowledge and sharing of expertise.

Trade associations

The three trade associations were broadly supportive of the recommendation to disseminate and share knowledge across the network.

1. Responses to the Veterinary section of the RSS
   1. Qualitative analysis
      1. Summary of responses to question 3 and 6

Question 3: “What are your overall views on the RSS?”

Question 6: “Are there any significant elements missing in this strategy? Please elaborate which ones”

Farming and animal owner organisations

Two responses recommend adding the following elements to the strategy:

- ethical and welfare improvements in research
- create a low resource way to monitor and disseminate knowledge
- create a low resource way to communicate and act on pharmacovigilance data
- companies or national governments could contribute to developing novel patient side tests for antibiotics

Veterinarian

There were five responses. Critical comments received were that the strategy focused too much on innovative therapies, rather than prevention, and did not incorporate sufficiently the 2019 regulations on veterinary medicines medicated feedstuffs. As in comments from some other stakeholder groups, the lack of a ‘One Health’ approach was noted.

Veterinarian organisations

Overall, the six responses welcomed the strategy but highlighted various opportunities for improvement. Some mentioned the need for more cross-fertilisation between human and veterinary issues and the need for the strategy to reflect this.

Learned society, academia and European research infrastructures and 'other scientific organisations'

Learned society (N=2), academia (N=2), European research infrastructures (N=3) and 'other scientific organisations' (N=3) were generally positive towards the strategy. Some advocated for improved alignment between the strategies for human and veterinary medicines (e.g. via using results from horizon scanning for medicines for veterinary medicines or by reducing animal use in quality control and safety testing in human medicinal product development). Several pointed out missing elements and points of attention.

EU regulatory partner/EU institution

There were six responses. The need for a greater One Health approach, in particular in relation to pharmaceuticals in the environment, was highlighted. References were made of the need for the strategy linking into the European Commission’s ‘Strategic Approach to Pharmaceuticals in the Environment’ (COM(2019) 128).

Pharmaceutical industry (SMEs, trade associations)

Overall responses from SMEs (N=1) and trade associations (N=4) welcomed the strategy and the drive to modernise and improve regulatory science. The draft was seen as a good foundation but needed further elaboration to achieve the final strategy.

- - 1. Summary of responses to questions 5 and 7

Question 5: “Please identify the top three Core Recommendations (in order of importance) that you believe will deliver the most significant change in the regulatory system over the next five years and why.”

Question 7: “The following is to allow more detailed feedback on prioritisation, which will also help shape the future application of resources. Your further input is therefore highly appreciated. Please choose for each row the option which most closely reflects your opinion. For areas outside your interest or experience, please leave blank. Should you wish to comment on any of the Core Recommendations (and their underlying actions) there is an option to do so.”

Due to the limited number of responses to the veterinary consultation, the summaries have been created per Core Recommendation, rather than per stakeholder group.

Goal 1: Catalysing the integration of science and technology in medicines development

1. **Transform the regulatory framework for innovative veterinary medicines**

The responses (1 Individual member of the public, 1 Veterinarian, 1 Farming and animal owner organisation, 2 Healthcare professionals’ organisations, 2 Regulatory partners / EU Institutions, 2 Other scientific organisations, 1 SME and 4 Trade associations) were supportive of the Core Recommendation. Respondents saw opportunities for immunotherapies. However, they cautioned that the veterinary sector is different from the human medicines sector, and these differences need to be considered. They were also at pains to emphasise the need for a flexible and rapid system to avoid innovation being dependent on changes in the core regulations.

1. **Reinforce and further embed application of the 3Rs principles**

The responses (1 Academic researcher, 2 Veterinarians, 2 EU Regulatory partners / EU Institutions, 2 Other scientific organisations and 1 Trade association) were generally supportive of the 3R principles: replacement, reduction and refinement of animal testing. They stressed the need to internationally harmonise standards.

1. **Facilitate implementation of novel manufacturing models**

The responses (1 Veterinarian, 1 Other scientific organisation and 3 Trade associations) saw it as important, particularly for innovative manufacturing technology and quality assurance through e.g. batch release

Goal 2: Driving collaborative evidence generation - improving the scientific quality of evaluations

1. **Update Environmental Risk Assessments in line with the latest scientific knowledge**

The responses (1 Veterinarian, 2 EU Regulatory partner / EU Institution and 2 Trade associations) had divergent views, but expressed the need for proportionality in ERA.

1. **Apply the latest scientific principles to the assessment of the safety of residues of veterinary medicines**

The responses (2 trade associations, and 1 veterinarian) were supportive of the recommendation.

1. **Collaborate with stakeholders to modernise veterinary pharmacoepidemiology and pharmacovigilance**

The responses (1 Individual member of the public, 1 Veterinarian, 3 EU Regulatory partners / EU Institutions and 2 Trade associations) mainly viewed the modernisation of veterinary pharmacoepidemiology and pharmacovigilance as important, welcoming a new system for pharmacovigilance.

1. **Develop new and improved communication and engagement channels and methods to reach out to stakeholders**

The responses (1 Veterinarian and 2 Trade associations) supported the Core Recommendation and requested that improved communication should be well thought through to avoid misinterpretation.

1. **Develop new approaches to improve the benefit-risk assessment of veterinary medicinal products**

The responses (1 Veterinarian, 2 Healthcare professionals’ organisations, 4 EU Regulatory partners / EU Institutions, 1 SME and 2 Trade associations) generally supported the Core Recommendation, particularly when considering veterinary vaccines and residues. Responses also requested that new methods for benefit-risk assessment should be flexible and should avoid being too risk averse.

Goal 3: Addressing emerging health threats and availability/therapeutic challenges

The responses (1 Veterinarian, 4 Healthcare professional organisations, 3 EU Regulatory partner / EU Institution and 3 Trade associations) were generally supportive of the Core Recommendation and taking a strong one health approach. They requested the recommendation include examples of alternatives to antimicrobials. However, they were wary of maintaining enough treatment availability.

1. **Continue to promote the responsible use of antimicrobials and their alternatives**

The responses (1 Veterinarian, 4 Healthcare professionals’ organisations, 3 EU Regulatory partners / EU Institutions and 3 Trade associations) were generally supportive of the Core Recommendation and taking a strong One Health approach, which is the principle which recognises that human and animal health are interconnected, that diseases are transmitted from humans to animals and vice versa and must therefore be tackled in both (12). It also encompasses the environment, another link between humans and animals and likewise a potential source of new resistant microorganisms. They requested the recommendation include examples of alternatives to antimicrobials. However, they cautioned that enough treatment options should remain available.

1. **Coordinate Network activities to improve data collection on antimicrobial use in animals**

The responses were (1 Veterinarian and 1 EU Regulatory partner / EU Institutions and hence cannot be summarised.

1. **Engage with stakeholders to minimise the risks of antiparasitic resistance**

The responses (1 Veterinarian and 1 Trade association) had divergence in their perception of the importance of the recommendation, and to which animal categories it should be focussed towards: a restricted population or all species.

1. **Promote and support development of veterinary vaccines**

The responses (1 EU Regulatory partner / EU Institution, 1 Other scientific organisation and 3 Trade associations) supported the Core Recommendation, in particular highlighting its importance for reducing use of antimicrobials in animals.

Goal 4: Enabling and leveraging research and innovation in regulatory science

1. **Develop network-led partnerships with academia to undertake fundamental research in strategic areas of regulatory science**

The responses (1 healthcare professional organisation, 2 other scientific organisations, 1 Veterinarian and 1 Trade association) gave strong support to the Core Recommendation but were wary on the need to ensure this research can be applied towards animal benefit.

1. **Leverage collaborations between academia and network scientists to address rapidly emerging regulatory science research questions**

The responses (1 healthcare professional organisation, 1 academic researcher, 1 EU Regulatory partner / EU Institution and 1 Trade association), supported the Core Recommendation, requesting it include multi-stakeholder collaboration.

1. **Identify and enable access to the best expertise across Europe and internationally**

The responses (1 EU Regulatory partner / EU Institution, 1 Veterinarian, 1 SME and 1 trade association) considered this very important now and for the future.

1. **Disseminate and exchange knowledge, expertise and innovation across the network and to its stakeholders**

The responses (2 EU Regulatory partner /EU Institution, 1 Veterinarian and 1 trade association) considered this important, and suggested expansion to a multi-disciplinary range of stakeholders.

1. Survey questions

Public consultation on EMA Regulatory Science to 2025

| Please note that the Word forms are an aid for respondents who wish to collaborate and agree on the responses before filling out the online questionnaire. They are not to be submitted to EMA as a response to the questionnaire.  All submissions must be made via the online questionnaire. |
| --- |

Fields marked with * are mandatory.

- Name

- Email


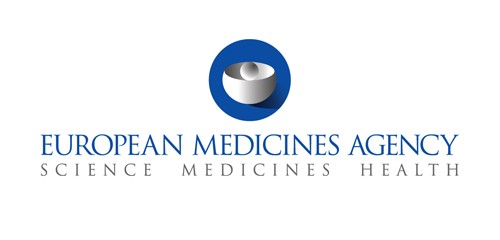


**Introduction**

### The purpose of this public consultation is to seek views from EMA’s stakeholders, partners and the general public on EMA’s proposed strategy on Regulatory Science to 2025 and whether it meets stakeholders’ needs. By highlighting where stakeholders see the need as greatest, you have the opportunity to jointly shape a vision for regulatory science that will in turn feed into the wider EU network strategy in the period 2020-25.

The views being sought on the proposed strategy refer both to the extent and nature of the broader strategic goals and core recommendations. We also seek your views on whether the specific underlying actions proposed are the most appropriate to achieve these goals.

The questionnaire will remain open until June 30, 2019. In case of any queries, please [contact: RegulatoryScience2025@ema.europa.eu.](mailto:RegulatoryScience2025@ema.europa.eu)

**Completing the questionnaire**

### This questionnaire should be completed once you have read the draft strategy document. The survey is divided into two areas: proposals for human regulatory science and proposals for veterinary regulatory science. You are invited to complete the section which is most relevant to your area of interest or both areas as you prefer.

We thank you for taking the time to provide your input; your responses will help to shape and prioritise our future actions in the field of regulatory science.

**Data Protection**

### By participating in this survey, your submission will be assessed by EMA. EMA collects and stores your personal data for the purpose of this survey and, in the interest of transparency, your submission will be made publicly available.

For more information about the processing of personal data by EMA, please read the [privacy](https://www.ema.europa.eu/en/about-us/legal/privacy-statement)

[statement](https://www.ema.europa.eu/en/about-us/legal/privacy-statement).

**Questionnaire**

**Question 1: What stakeholder, partner or group do you represent?**

### Individual member of the public*

Patient or Consumer Organisation

Healthcare professional organization

Learned society

Farming and animal owner organisation

Academic researcher

Healthcare professional

Veterinarian

European research infrastructure

Research funder

Other scientific organisation

EU Regulatory partner / EU Institution

Health technology assessment body

Payer

Pharmaceutical industry**

Non-EU regulator / Non-EU regulatory body

Other

*** *Please indicate the capacity in which you are responding:***

*between 1 and 3 choices*

Citizen

### Patient

### Carer

Animal owner

Farmer

**** *Please specify:***

*between 1 and 1 choices*

Individual company

Trade association

SME

***Please specify: Press/media/NGO/Not-for profit organisation/other scientific organisations/policy maker, etc.***

***Name of organisation (if applicable):***

**Question 2: Which part of the proposed strategy document are you commenting upon?**

### Both

**Question 3 (human and veterinary): What are your overall views about the strategy proposed in EMA’s Regulatory Science to 2025?**

*Please note you will be asked to comment on the core recommendations and underlying actions in the subsequent questions.*

*Please remember to specify if a particular comment relates specifically to the human or veterinary part.*

**Question 4 (human and veterinary): Do you consider the strategic goals appropriate?**

Strategic goal 1: Catalysing the integration of science and technology in medicines development **(h & v)**

Yes

No

### Comments on strategic goal 1 (h & v):

*Please note you will be asked to comment on the core recommendations and underlying actions in the subsequent questions.*

*Please remember to specify if a particular comment relates specifically to the human or veterinary part.*

Strategic goal 2: Driving collaborative evidence generation – improving the scientific quality of evaluations **(h & v)**

Yes

No

Comments on strategic goal 2 (h & v):

*Please note you will be asked to comment on the core recommendations and underlying actions in the subsequent questions.*

*Please remember to specify if a particular comment relates specifically to the human or veterinary part.*

Strategic goal 3: Advancing patient-centred access to medicines in partnership with healthcare systems **(h-only)**

Yes

No

### Comments on strategic goal 3 (h):

*Please note you will be asked to comment on the core recommendations and underlying actions in the subsequent questions*.

*Please remember to specify if a particular comment relates specifically to the human or veterinary part.*

Strategic goal 4 (human) / 3 (veterinary): Addressing emerging health threats and availability/therapeutic challenges **(h & v)**

Yes

No

### Comments on strategic goal 4 (h) / 3 (v):

*Please note you will be asked to comment on the core recommendations and underlying actions in the subsequent questions*.

*Please remember to specify if a particular comment relates specifically to the human or veterinary part.*

Strategic goal 5 (human) / 4 (veterinary): Enabling and leveraging research and innovation in regulatory science **(h & v)**

Yes

No

### Comments on strategic goal 5 (h) / 4 (v):

*Please note you will be asked to comment on the core recommendations and underlying actions in the subsequent questions.*

*Please remember to specify if a particular comment relates specifically to the human or veterinary part*.

**Question 5 (human): Please identify the top three core recommendations (in order of importance) that you believe will deliver the most significant change in the regulatory system over the next five years and why.**

First choice (h)

1. Support developments in precision medicine, biomarkers and ‘omics’

2. Support translation of Advanced Therapy Medicinal Products cell, genes and tissue-based products into patient treatments

3. Promote and invest in the Priority Medicines scheme (PRIME)

4. Facilitate the implementation of novel manufacturing technologies

5. Create an integrated evaluation pathway for the assessment of medical devices, in vitro diagnostics and borderline products

6. Develop understanding of and regulatory response to nanotechnology and new materials’ utilisation in pharmaceuticals

7. Diversify and integrate the provision of regulatory advice along the development continuum

8. Leverage novel non-clinical models and 3Rs

9. Foster innovation in clinical trials

10. Develop the regulatory framework for emerging digital clinical data generation

11. Expand benefit-risk assessment and communication

12. Invest in special populations initiatives

13. Optimise capabilities in modelling and simulation and extrapolation

14. Exploit digital technology and artificial intelligence in decision-making

15. Contribute to HTAs’ preparedness and downstream decision-making for innovative medicines

16. Bridge from evaluation to access through collaboration with Payers

17. Reinforce patient relevance in evidence generation

18. Promote use of high-quality real world data (RWD) in decision-making

19. Develop network competence and specialist collaborations to engage with big data

20. Deliver real-time electronic Product Information (ePI)

21. Promote the availability and uptake of biosimilars in healthcare systems

22. Further develop external communications to promote trust and confidence in the EU regulatory system

23. Implement EMA’s health threats plan, ring-fence resources and refine preparedness approaches

24. Continue to support development of new antimicrobials and their alternatives

25. Promote global cooperation to anticipate and address supply challenges

26. Support innovative approaches to the development and post-authorisation monitoring of vaccines

27. Support the development and implementation of a repurposing framework

28. Develop network-led partnerships with academia to undertake fundamental research in strategic areas of regulatory science

29. Leverage collaborations between academia and network scientists to address rapidly emerging regulatory science research questions

30. Identify and enable access to the best expertise across Europe and internationally

31. Disseminate and share knowledge, expertise and innovation across the regulatory network and to its stakeholders

### 1st choice (h): please comment on your choice, the underlying actions proposed and identify any additional actions you think might be needed to effect these changes.

Second choice (h)

1. Support developments in precision medicine, biomarkers and ‘omics’

2. Support translation of Advanced Therapy Medicinal Products cell, genes and tissue-based products into patient treatments

3. Promote and invest in the Priority Medicines scheme (PRIME)

4. Facilitate the implementation of novel manufacturing technologies

5. Create an integrated evaluation pathway for the assessment of medical devices, in vitro diagnostics and borderline products

6. Develop understanding of and regulatory response to nanotechnology and new materials’ utilisation in pharmaceuticals

7. Diversify and integrate the provision of regulatory advice along the development continuum

8. Leverage novel non-clinical models and 3Rs

9. Foster innovation in clinical trials

10. Develop the regulatory framework for emerging digital clinical data generation

11. Expand benefit-risk assessment and communication

12. Invest in special populations initiatives

13. Optimise capabilities in modelling and simulation and extrapolation

14. Exploit digital technology and artificial intelligence in decision-making

15. Contribute to HTAs’ preparedness and downstream decision-making for innovative medicines

16. Bridge from evaluation to access through collaboration with Payers

17. Reinforce patient relevance in evidence generation

18. Promote use of high-quality real world data (RWD) in decision-making

19. Develop network competence and specialist collaborations to engage with big data

20. Deliver real-time electronic Product Information (ePI)

21. Promote the availability and uptake of biosimilars in healthcare systems

22. Further develop external communications to promote trust and confidence in the EU regulatory system

23. Implement EMA’s health threats plan, ring-fence resources and refine preparedness approaches

24. Continue to support development of new antimicrobials and their alternatives

25. Promote global cooperation to anticipate and address supply challenges

26. Support innovative approaches to the development and post-authorisation monitoring of vaccines

27. Support the development and implementation of a repurposing framework

28. Develop network-led partnerships with academia to undertake fundamental research in strategic areas of regulatory science

29. Leverage collaborations between academia and network scientists to address rapidly emerging regulatory science research questions

30. Identify and enable access to the best expertise across Europe and internationally

31. Disseminate and share knowledge, expertise and innovation across the regulatory network and to its stakeholders

### 2nd choice (h): please comment on your choice, the underlying actions proposed and identify any additional actions you think might be needed to effect these changes.

Third choice (h)

1. Support developments in precision medicine, biomarkers and ‘omics’

2. Support translation of Advanced Therapy Medicinal Products cell, genes and tissue-based products into patient treatments

3. Promote and invest in the Priority Medicines scheme (PRIME)

4. Facilitate the implementation of novel manufacturing technologies

5. Create an integrated evaluation pathway for the assessment of medical devices, in vitro diagnostics and borderline products

6. Develop understanding of and regulatory response to nanotechnology and new materials’ utilisation in pharmaceuticals

7. Diversify and integrate the provision of regulatory advice along the development continuum

8. Leverage novel non-clinical models and 3Rs

9. Foster innovation in clinical trials

10. Develop the regulatory framework for emerging digital clinical data generation

11. Expand benefit-risk assessment and communication

12. Invest in special populations initiatives

13. Optimise capabilities in modelling and simulation and extrapolation

14. Exploit digital technology and artificial intelligence in decision-making

15. Contribute to HTAs’ preparedness and downstream decision-making for innovative medicines

16. Bridge from evaluation to access through collaboration with Payers

17. Reinforce patient relevance in evidence generation

18. Promote use of high-quality real world data (RWD) in decision-making

19. Develop network competence and specialist collaborations to engage with big data

20. Deliver real-time electronic Product Information (ePI)

21. Promote the availability and uptake of biosimilars in healthcare systems

22. Further develop external communications to promote trust and confidence in the EU regulatory system

23. Implement EMA’s health threats plan, ring-fence resources and refine preparedness approaches

24. Continue to support development of new antimicrobials and their alternatives

25. Promote global cooperation to anticipate and address supply challenges

26. Support innovative approaches to the development and post-authorisation monitoring of vaccines

27. Support the development and implementation of a repurposing framework

28. Develop network-led partnerships with academia to undertake fundamental research in strategic areas of regulatory science

29. Leverage collaborations between academia and network scientists to address rapidly emerging regulatory science research questions

30. Identify and enable access to the best expertise across Europe and internationally

31. Disseminate and share knowledge, expertise and innovation across the regulatory network and to its stakeholders

### 3rd choice (h): please comment on your choice, the underlying actions proposed and identify any additional actions you think might be needed to effect these changes.

**Question 5 (veterinary): Please identify the top three core recommendations (in order of importance) that you believe will deliver the most significant change in the regulatory system over the next five years and why.**

First choice (v)

Please note that veterinary goals start at no.32

32. Transform the regulatory framework for innovative veterinary medicines

33. Reinforce and further embed application of the 3Rs principles

34. Facilitate implementation of novel manufacturing models

35. Update Environmental Risk Assessments in line with the latest scientific knowledge

36. Apply the latest scientific principles to the assessment of the safety of residues of veterinary medicines

37. Collaborate with stakeholders to modernise veterinary pharmacoepidemiology and pharmacovigilance

38. Develop new and improved communication and engagement channels and methods to reach out to stakeholders

39. Develop new approaches to improve the benefit-risk assessment of veterinary medicinal products

40. Continue to promote the responsible use of antimicrobials and their alternatives

41. Coordinate Network activities to improve data collection on antimicrobial use in animals

42. Engage with stakeholders to minimise the risks of antiparasitic resistance

43. Promote and support development of veterinary vaccines

44. Develop network-led partnerships with academia to undertake fundamental research in strategic areas of regulatory science

45. Leverage collaborations between academia and network scientists to address rapidly emerging regulatory science research questions

46. Identify and enable access to the best expertise across Europe and internationally

47. Disseminate and exchange knowledge, expertise and innovation across the network and to its stakeholders

### 1st choice (v): please comment on your choice, the underlying actions proposed and identify any additional actions you think might be needed to effect these changes.

Second choice (v)

Please note that veterinary goals start at no.32

32. Transform the regulatory framework for innovative veterinary medicines

33. Reinforce and further embed application of the 3Rs principles

34. Facilitate implementation of novel manufacturing models

35. Update Environmental Risk Assessments in line with the latest scientific knowledge

36. Apply the latest scientific principles to the assessment of the safety of residues of veterinary medicines

37. Collaborate with stakeholders to modernise veterinary pharmacoepidemiology and pharmacovigilance

38. Develop new and improved communication and engagement channels and methods to reach out to stakeholders

39. Develop new approaches to improve the benefit-risk assessment of veterinary medicinal products

40. Continue to promote the responsible use of antimicrobials and their alternatives

41. Coordinate Network activities to improve data collection on antimicrobial use in animals

42. Engage with stakeholders to minimise the risks of antiparasitic resistance

43. Promote and support development of veterinary vaccines

44. Develop network-led partnerships with academia to undertake fundamental research in strategic areas of regulatory science

45. Leverage collaborations between academia and network scientists to address rapidly emerging regulatory science research questions

46. Identify and enable access to the best expertise across Europe and internationally

47. Disseminate and exchange knowledge, expertise and innovation across the network and to its stakeholders

### 2nd choice (v): please comment on your choice, the underlying actions proposed and identify any additional actions you think might be needed to effect these changes.

Third choice (v)

Please note that veterinary goals start at no.32

32. Transform the regulatory framework for innovative veterinary medicines

33. Reinforce and further embed application of the 3Rs principles

34. Facilitate implementation of novel manufacturing models

35. Update Environmental Risk Assessments in line with the latest scientific knowledge

36. Apply the latest scientific principles to the assessment of the safety of residues of veterinary medicines

37. Collaborate with stakeholders to modernise veterinary pharmacoepidemiology and pharmacovigilance

38. Develop new and improved communication and engagement channels and methods to reach out to stakeholders

39. Develop new approaches to improve the benefit-risk assessment of veterinary medicinal products

40. Continue to promote the responsible use of antimicrobials and their alternatives

41. Coordinate Network activities to improve data collection on antimicrobial use in animals

42. Engage with stakeholders to minimise the risks of antiparasitic resistance

43. Promote and support development of veterinary vaccines

44. Develop network-led partnerships with academia to undertake fundamental research in strategic areas of regulatory science

45. Leverage collaborations between academia and network scientists to address rapidly emerging regulatory science research questions

46. Identify and enable access to the best expertise across Europe and internationally

47. Disseminate and exchange knowledge, expertise and innovation across the network and to its stakeholders

### 3rd choice (v): please comment on your choice, the underlying actions proposed and identify any additional actions you think might be needed to effect these changes.

**Question 6 (human and veterinary): Are there any significant elements missing in this strategy. Please elaborate which ones (h & v)**

*Please remember to specify if a particular comment relates specifically to the human or veterinary part.*

**Question 7 (human): The following is to allow more detailed feedback on prioritisation, which will also help shape the future application of resources. Your further input is therefore highly appreciated. Please choose for each row the option which most closely reflects your opinion. For areas outside your interest or experience, please leave blank.**

*Should you wish to comment on any of the core recommendations (and their underlying actions) there is an option to do so.*

**Strategic goal 1: Catalysing the integration of science and technology in medicines development (h)**

|  | Very important | Important | Moderately important | Less important | Not important |
| --- | --- | --- | --- | --- | --- |
| 1. Support developments in precision medicine, biomarkers and ‘omics’ |  |  |  |  |  |
| 2. Support translation of Advanced Therapy Medicinal Products cell, genes and tissue-based products into patient treatments |  |  |  |  |  |
| 3. Promote and invest in the Priority Medicines scheme (PRIME) |  |  |  |  |  |
| 4. Facilitate the implementation of novel manufacturing technologies |  |  |  |  |  |
| 5. Create an integrated evaluation pathway for the assessment of medical devices, in vitro diagnostics and borderline products |  |  |  |  |  |
| 6. Develop understanding of and regulatory response to nanotechnology and new materials’ utilisation in pharmaceuticals |  |  |  |  |  |
| 7. Diversify and integrate the provision of regulatory advice along the development continuum |  |  |  |  |  |

Please feel free to comment on any of the above core recommendations or their underlying actions. **Kindly indicate the number of the recommendation** you are commenting on:

**Strategic goal 2: Driving collaborative evidence generation – improving the scientific quality of evaluations (h)**

|  | Very important | Important | Moderately important | Less important | Not important |
| --- | --- | --- | --- | --- | --- |
| 8. Leverage novel non- clinical models and 3Rs |  |  |  |  |  |
| 9. Foster innovation in clinical trials |  |  |  |  |  |
| 10. Develop the regulatory framework for emerging digital clinical data generation |  |  |  |  |  |
| 11. Expand benefit- risk assessment and communication |  |  |  |  |  |
| 12. Invest in special populations initiatives |  |  |  |  |  |
| 13. Optimise capabilities in modelling and simulation and extrapolation |  |  |  |  |  |
| 14. Exploit digital technology and artificial intelligence in decision-making |  |  |  |  |  |

Please feel free to comment on any of the above core recommendations or their underlying actions. **Kindly indicate the number of the recommendation you are commenting on**:

**Strategic goal 3: Advancing patient-centred access to medicines in partnership with healthcare systems (h)**

|  | Very important | Important | Moderately important | Less important | Not important |
| --- | --- | --- | --- | --- | --- |
| 15. Contribute to HTAs’ preparedness and downstream decision-making for innovative medicines |  |  |  |  |  |
| 16. Bridge from evaluation to access through collaboration with Payers |  |  |  |  |  |
| 17. Reinforce patient relevance in evidence generation |  |  |  |  |  |
| 18. Promote use of high-quality real world data (RWD) in decision- making |  |  |  |  |  |
| 19. Develop network |  |  |  |  |  |
| competence and specialist collaborations to engage with big data |  |  |  |  |  |
| 20. Deliver real-time electronic Product Information (ePI) |  |  |  |  |  |
| 21. Promote the availability and uptake of biosimilars in healthcare systems |  |  |  |  |  |
| 22. Further develop external communications to promote trust and confidence in the EU regulatory system |  |  |  |  |  |

Please feel free to comment on any of the above core recommendations or their underlying actions. **Kindly indicate the number of the recommendation you are commenting on**:

**Strategic goal 4: Addressing emerging health threats and availability/therapeutic challenges (h)**

|  | Very important | Important | Moderately important | Less important | Not important |
| --- | --- | --- | --- | --- | --- |
| 23. Implement EMA’s health threats plan, ring- fence resources and refine preparedness approaches |  |  |  |  |  |
| 24. Continue to support development of new antimicrobials and their alternatives |  |  |  |  |  |
| 25. Promote global cooperation to anticipate and address supply challenges |  |  |  |  |  |
| 26. Support innovative approaches to the development and post- authorisation monitoring of vaccines |  |  |  |  |  |
| 27. Support the development and implementation of a repurposing framework |  |  |  |  |  |

Please feel free to comment on any of the above core recommendations or their underlying actions. **Kindly indicate the number of the recommendation you are commenting on**:

**Strategic goal 5: Enabling and leveraging research and innovation in regulatory science (h)**

|  | Very important | Important | Moderately important | Less important | Not important |
| --- | --- | --- | --- | --- | --- |
| 28. Develop network- led partnerships with academia to undertake fundamental research in strategic areas of regulatory science |  |  |  |  |  |
| 29. Leverage collaborations between academia and network scientists to address rapidly emerging regulatory science research questions |  |  |  |  |  |
| 30. Identify and enable access to the best expertise across |  |  |  |  |  |
| 31. Disseminate and share knowledge, expertise and innovation across the  regulatory network and to its stakeholders |  |  |  |  |  |

Please feel free to comment on any of the above core recommendations or their underlying actions. **Kindly indicate the number of the recommendation you are commenting on**:

**Question 7 (veterinary): The following is to allow more detailed feedback on prioritisation, which will also help shape the future application of resources. Your further input is therefore highly appreciated. Please choose for each row the option which most closely reflects your opinion. For areas outside your interest or experience, please leave blank.**

*Should you wish to comment on any of the core recommendations (and their underlying actions) there is an option to do so.*

**Strategic goal 1: Catalysing the integration of science and technology in medicines development (v)**

|  | Very important | Important | Moderately important | Less important | Not important |
| --- | --- | --- | --- | --- | --- |
| 32. Transform the regulatory framework  for innovative veterinary medicines |  |  |  |  |  |
| 33. Reinforce and further embed application of the 3Rs principles |  |  |  |  |  |
| 34. Facilitate implementation of novel manufacturing models |  |  |  |  |  |

Please feel free to comment on any of the above core recommendations or their underlying actions. **Kindly indicate the number of the recommendation you are commenting on**:

**Strategic goal 2: Driving collaborative evidence generation – improving the scientific quality of evaluations (v)**

|  | Very important | Important | Moderately important | Less important | Not important |
| --- | --- | --- | --- | --- | --- |
| 35. Update Environmental Risk Assessments in line with the latest scientific knowledge |  |  |  |  |  |
| 36. Apply the latest scientific principles to the assessment of the safety of residues of veterinary medicines |  |  |  |  |  |
| 37. Collaborate with stakeholders to modernise veterinary pharmacoepidemiology and pharmacovigilance |  |  |  |  |  |
| 38. Develop new and improved communication and engagement channels and methods to reach out to stakeholders |  |  |  |  |  |
| 39. Develop new approaches to improve the benefit-risk assessment of veterinary medicinal products |  |  |  |  |  |

Please feel free to comment on any of the above core recommendations or their underlying actions. **Kindly indicate the number of the recommendation you are commenting on**:

**Strategic goal 3: Addressing emerging health threats and availability/therapeutic challenges (v)**

|  | Very important | Important | Moderately important | Less important | Not important |
| --- | --- | --- | --- | --- | --- |
| 40. Continue to promote the responsible use of antimicrobials and their alternatives |  |  |  |  |  |
| 41. Coordinate Network activities to improve data collection on antimicrobial use in animals |  |  |  |  |  |
| 42. Engage with stakeholders to minimise the risks of antiparasitic resistance |  |  |  |  |  |
| 43. Promote and support development of veterinary vaccines |  |  |  |  |  |

Please feel free to comment on any of the above core recommendations or their underlying actions. **Kindly indicate the number of the recommendation you are commenting on**:

**Strategic goal 4: Enabling and leveraging research and innovation in regulatory science (v)**

|  | Very important | Important | Moderately important | Less important | Not important |
| --- | --- | --- | --- | --- | --- |
| 44. Develop network- led partnerships with academia to undertake fundamental research in strategic areas of regulatory science |  |  |  |  |  |
| 45. Leverage collaborations between academia and network scientists to address rapidly emerging regulatory science research questions |  |  |  |  |  |
| 46. Identify and enable access to the best expertise across Europe and internationally |  |  |  |  |  |
| 47. Disseminate and share knowledge, expertise and innovation across the regulatory network and to its stakeholders |  |  |  |  |  |

Please feel free to comment on any of the above core recommendations or their underlying actions. **Kindly indicate the number of the recommendation you are commenting on**:

### Thank you very much for completing the survey. We value your opinion and encourage you to inform others who you know would be interested.

1. List of acronyms

AI - Artificial Intelligence

AMR - Antimicrobial Resistance

ATMP - Advanced Therapy Medicinal Product

BR – Benefit-risk

CODEX - Codex Alimentarius

CQA - Critical Quality Attribute

CVMP - Committee for Veterinary Medicinal Products (EMA)

DG RTD - European Commission Directorate General for Research and Innovation

DG AGRI - European Commission Directorate General for Agriculture and Rural Development

DG JRC - European Commission Directorate General Joint Research Centre

DG SANTE - European Commission Directorate General for Health and Food Safety

EC - European Commission

ECHA - European Chemicals Agency

EEA – European Economic Area

EFSA - European Food Safety Authority

EMA - European Medicines Agency

EMRN - European Medicines Regulatory Network, the EU network

ePI - electronic Product Information

ERA - Environmental Risk Assessment

FDA - Food and Drug Administration (USA)

FIM - First-In-Man

GCP - Good Clinical Practice

GMP - Good Manufacturing Practice

HMA - Heads of Medicine Agencies

HTA - Health Technology Assessment body

ICDRA - International Coalition of Drug Regulatory Authorities

ICH - International Conference on Harmonisation of Technical Requirements for Registration of Pharmaceuticals for Human Use

ICMRA - International Coalition of Medicines Regulatory Authorities

IMI - Innovative Medicines Initiative

IVDR – EU In Vitro Diagnostic Regulation (2017/746)

JEFCA - Joint FAO/WHO Expert Committee on Food Additives

MAA - Marketing Authorisation Application

MDR – EU Medical Device Regulation (2017/745)

MRL - Maximum Residue Limit

NAMs - New Approach Methodologies

NITAG - National Immunisation Technical Advisory Group

OECD - Organisation for Economic Co-operation and Development

OIE - World Organisation for Animal Health

PI - Product Information

PIC/S - Pharmaceutical Inspection Collaboration Scheme

PK/PD - Pharmacokinetics/Pharmacodynamics

PMDA - Pharmaceuticals and Medical Devices Agency (Japan)

PRIME - Priority Medicines Scheme

PRO - Patient-Reported Outcome

PROM - Patient-Reported Outcome Measure

RWD - Real World Data

SciCoBo - Scientific Coordination Board

SEND - Standard for Exchange of Nonclinical Data

SME - Small or Medium-sized Enterprise

STAMP - Commission Group on Safe and Timely Access to Medicines for Patients

VICH - Veterinary International Conference on Harmonization

WHO - World Health Organization
